# Automated Fidelity Monitoring of Lay-Delivered Mental Health Interventions Using Large Language Models: Development and Pilot Validation of shamiriAI in Kenya

**DOI:** 10.2196/95063

**Published:** 2026-07-23

**Authors:** Shadrack Lilan, Brandon Mochama, Tom Osborn, Wendy Mmbone, Rachael Kilonzo, Faith Kamau, Rahim Daya, Christine Wasanga

**Affiliations:** 1 Shamiri Institute, 13th Floor, CMS Africa, Chania Avenue, Nairobi, 00505, Kenya, +254 (0) 112540760; 2 Department of Psychology, Kenyatta University, Nairobi, Nairobi County, Kenya

**Keywords:** fidelity monitoring, task-shifting, automatic speech recognition, large language models, clinical supervision, adolescent mental health, multilingual, low- and middle-income countries

## Abstract

**Background:**

Task-shifting can help close the mental health treatment gap in low- and middle-income countries, but its effectiveness depends on ongoing supervision, which is hard to scale. AI tools that process session recordings and generate structured fidelity feedback could offer a scalable alternative; yet, to our knowledge, none have been developed or validated for lay-delivered, multilingual, group-format interventions in low-resource settings.

**Objective:**

We developed and pilot-validated shamiriAI (ShamiriAI Institute), an automated fidelity-monitoring tool for lay-delivered mental health interventions, embedded within the Shamiri school-based program in Kenya.

**Methods:**

Across 6 secondary schools in Ngong Hub, Kajiado County, Kenya (May-September 2025), shamiriAI processed session audio from 47 lay providers through a 5-stage pipeline: ingestion, multilingual automatic speech recognition (ASR) with prosodic feature extraction, personally identifiable information scrubbing, large language model–based fidelity inference, and supervisor reports. The following two aims were assessed: (1) ASR performance on a held-out test set of manually transcribed sessions and (2) interrater reliability between shamiriAI and independent human supervisor ratings across 52 sessions (38 AI-augmented and 14 standard) on 6 domains (Required Contents, Specifics, Thoroughness, Clarity, Skill, and Purity; 1‐7 scale). Reliability used intraclass correlation coefficients, Bland-Altman analysis, adjacent-agreement rates, paired *t* tests with Holm-Bonferroni correction, and Gwet AC2 (ordinal weights) across 3 formulations of the human reference.

**Results:**

The ASR model achieved a character error rate of 0.19, word error rate of 0.34, and cosine semantic similarity of 0.77, indicating strong meaning preservation in code-switched speech. AI fidelity scores were systematically lower than the human composite overall (mean 5.14, SD 0.77 vs mean 5.93, SD 0.57 ); Δ=−0.79; d=−1.16; *P*<.001). Primary intraclass correlation coefficients ranged from −0.06 to 0.20 across the 6 domains, and AC2 sensitivity analyses (against each individual rater and the rounded composite) corroborated this dimension-level ordering. Three patterns emerged: large systematic underrating on holistic dimensions (Required Contents: d=−3.48; Clarity d=−1.56); bidirectional medium-effect bias on facilitation dimensions (Thoroughness d=−0.99; Skill d=+0.87); and substantial agreement on Specifics, Skill, and Purity (Gwet AC2 0.69‐0.76 against the rounded composite), relative to a human-human AC2 ceiling of 0.42‐0.60 estimated in the same dataset. Exploratory, underpowered subgroup and per-arm checks found no preliminary evidence of bias by lay-provider sex or age band or of arm-level differences.

**Conclusions:**

Within this 52-session pilot, shamiriAI shows technically feasible multilingual ASR and a coherent, dimension-dependent reliability profile. Specifics, Purity, and Skill already reach substantial agreement, while underperformance on holistic dimensions (Required Contents and Clarity) reflects diagnosable misalignments in rubric interpretation and prompt design, specifying a concrete agenda for shamiriAI (version 2; Shamiri Institute). Whether AI-augmented supervision improves provider skill or student mental health outcomes will be tested in a planned cluster-randomized noninferiority trial.

## Introduction

### Background

We are in the middle of a global mental health crisis among young people [1-3]. Depression, anxiety, and related disorders are among the leading causes of illness and disability in people aged 10 to 24 [4], and their prevalence has risen sharply over the past 3 decades [2]. An estimated 279 million young people aged 10-24 years live with a mental health disorder [2,5]. Most receive no help. The treatment gap—the proportion of people who need care but do not receive it—is 40%-60% globally and exceeds 80% in low-resource settings, where the majority of the world’s young people live [6,7].

The core structural problem driving this gap is a global scarcity of providers [1,8,9]. The global median is 13.5 specialized mental health workers per 100,000 people [8]; the ratio falls to just 1.1‐2.4 per 100,000 in low- and middle-income countries, against 67.2 in high-income countries (HICs) [8,9]. Kenya, for instance, where 1 in 3 adolescents experience a mental health problem [10-13], has about 2 specialists per 100,000 people, most of them concentrated in urban centers [9,14]. Closing the treatment gap through specialist-led care alone is not feasible in the near term [15,16].

Task-shifting—training non-specialists to deliver structured, evidence-based interventions under supervision—has emerged as the leading strategy for expanding the workforce and closing the treatment gap [17-19]. Randomized trials across sub-Saharan Africa and South Asia demonstrate that lay providers—including community health workers [20], trained youth [21], and even grandmothers [22]—can deliver effective treatments for depression, anxiety, and trauma, producing clinically meaningful improvements that are sometimes comparable to those achieved by professionals [18,19,23].

The Shamiri Model in Kenya illustrates what task-shifting looks like in practice. Shamiri is a brief, 4-week group intervention for secondary school students built around strengths-based psychological principles—growth mindset, gratitude, and values affirmation [21,24]. It operates through a 3-tier delivery model. In the first tier, lay providers aged 18-22 years are trained to deliver weekly group sessions to cohorts of 6 to 15 students [21,25]. Clinical supervisors with backgrounds in psychology or social work form the second tier, overseeing lay providers and providing direct one-on-one support where needed [21,25]. Complex or severe cases are escalated to licensed clinicians in the third tier [26]. The structure maximizes reach by placing trained lay providers at the point of care; the model operates at the school level—where adolescents spend most of their time—without drawing on scarce specialists for routine cases [26,27]. Randomized controlled trials have demonstrated that the model significantly reduces depression and anxiety symptoms in secondary school students, with effects sustained at least 7 months postintervention [21,28,29].

Task-shifting has demonstrated that an expanded workforce can deliver effective care [18,23,30]. The harder problem is maintaining quality across that workforce as programs grow. Training a cohort of 20 lay providers is manageable; supervising hundreds of them, across dozens of sites, delivering sessions week after week, is a fundamentally different challenge—and one that existing models have not solved [25,26].

The answer, in every evidence-based framework for task-shifting, is supervision [17,25,31]. Supervision is the mechanism through which lay providers consolidate skills, receive corrective feedback, maintain fidelity to protocol, manage clinical risk, and sustain the psychological safety required to work with vulnerable youth [17,32-34]. Without it, skill drift is invisible—sessions degrade in quality, required elements go undelivered, and program managers have no way of knowing [17,25,33,34].

But high-quality supervision is difficult to scale. Traditional supervision models require trained professionals to manually review session recordings, meet individually or in groups with providers, and deliver timely, specific feedback [25,26,33]. As programs grow, supervision becomes a bottleneck. Supervisors can review only a fraction of sessions, feedback arrives late, and important behavioral information—tone, pacing, and interpersonal dynamics—is lost [25,35]. Supervision remains a central constraint on the quality and scalability of task-shifted mental health care.

Two advances offer a route forward. The first is the maturation of task-shifting models themselves. Models like Shamiri have shown that lay-delivered interventions can be implemented at scale with structured training and supervision systems—creating the operational infrastructure into which quality-assurance tools can be embedded [18,19,23,26,30]. The second is the emergence of AI tools capable of automating the observational and feedback functions of supervision. Advances in automatic speech recognition (ASR), natural language processing, and large language models (LLMs) have made it technically feasible to process session audio, estimate provider behaviors, score fidelity to clinical protocols, and generate structured feedback reports—potentially reducing the manual-review bottleneck through systematic, scalable monitoring [36-39]. Research teams in HICs have developed AI systems that achieve human-level reliability on session-quality ratings for individual cognitive behavioral therapy (CBT) in English [36,37,39], and recent work demonstrates that LLMs can score therapeutic constructs from transcribed sessions with strong psychometric properties and valid associations with outcomes [40]. The trajectory suggests AI-assisted fidelity monitoring is becoming viable—but viable under a specific set of conditions: individual therapy, adult populations, professional providers, and English-language settings in HICs.

Whether AI fidelity monitoring can be extended to the conditions that define most of the world’s task-shifted mental health care is entirely unestablished. Group delivery, lay providers, multilingual code-switched speech, minimal training data, and resource-constrained deployment contexts are not peripheral variations—they are the structural features of the settings where supervision is most needed and least available. To our knowledge, no automated fidelity-monitoring system has been developed for, or validated in, this combination of lay-delivered, multilingual, group-format care. The gap spans every axis that matters, including delivery format, workforce type, linguistic environment, and implementation constraints.

### Objectives

To address these gaps, we developed shamiriAI—an AI system designed to support fidelity monitoring and supervision for lay-delivered mental health interventions in multilingual, low-resource settings. shamiriAI processes raw session audio, performs multilingual ASR across English, Kiswahili, and Sheng, extracts prosodic features capturing group dynamics and facilitator behavior, and generates structured written feedback reports for clinical supervisors. It operates in an AI-in-the-loop configuration: supervisors remain the primary agents of support and clinical judgment; AI-generated feedback extends their observational reach, providing structured input on sessions that would otherwise go unreviewed.

This study reports the foundational validation work required before AI-augmented supervision can be meaningfully tested for its effect on provider skill or student outcomes. Before asking whether the system works clinically, 2 prior questions must be answered: can it accurately transcribe sessions in this linguistic environment, and are its fidelity assessments sufficiently aligned with expert human judgment to constitute credible supervisory inputs?

The objectives of this pilot study were twofold. The first was to evaluate the performance of shamiriAI’s multilingual ASR pipeline on a held-out test set of manually transcribed sessions, using metrics appropriate for code-switched, agglutinative speech. The second was to assess interrater reliability between shamiriAI-generated fidelity ratings and independent human supervisor ratings across 6 fidelity dimensions, examining the degree of agreement, the direction and magnitude of systematic bias, and the specific dimensions for which the AI most and least closely approximated expert human judgment.

These aims are explicitly foundational. They establish whether shamiriAI produces transcripts and fidelity ratings with sufficient accuracy to support future development—the essential first step toward an AI quality assurance infrastructure capable of supporting the supervision of lay providers at population scale across sub-Saharan Africa.

## Methods

### Study Design and Setting

We conducted a pilot validation study of shamiriAI, an AI-based supervision support tool, embedded within routine delivery of the Shamiri intervention across 6 secondary schools in Ngong Hub, Kajiado County, Kenya, between May and September 2025. The study evaluated the following two pilot aims: (1) the technical performance of shamiriAI’s ASR pipeline on a held-out test set of manually transcribed sessions, and (2) interrater reliability between shamiriAI-generated fidelity ratings and independent human supervisor ratings across 52 recorded sessions. These aims concern the technical properties of the system, not between-arm comparisons.

Lay providers (Fellows) were randomized to supervision arms at the start of the study. The sessions later recorded for validation were sampled independently at each site by lottery, without stratification by arm. Because assignment preceded session sampling, the validation set is imbalanced across arms (38 AI-augmented and 14 standard); the sampling mechanism is detailed under the Participants subheading.

The pilot was part of a larger 5-part implementation study examining optimization strategies for the Shamiri Model [41,42]. The complete protocol is available in Supplement A in Multimedia Appendix 1.

### Participants

Lay providers (Shamiri Fellows) are Kenyan young adults aged 18‐24 years recruited and trained to deliver the Shamiri intervention (refer to the Procedures subheading). All 64 Shamiri Fellows assigned to Ngong Hub were eligible; sex and age were collected via self-report. The 47 Fellows who participated in the parallel A/B test were randomized at project start to AI-augmented (n=34) or standard (n=13) supervision.

Audio recording for fidelity validation was a separate procedure. At each school session, the Hub Coordinator wrote the names of all lay providers present on slips of paper and drew 3 without replacement for recording, with no stratification by arm. This produced 52 recorded sessions, distributed unevenly across arms (38 AI-augmented and 14 standard) and across providers, reflecting natural variation in attendance and recording opportunity rather than a prespecified arm-balanced design.

Lay providers led group sessions for students aged 12‐21 years participating in the routine Shamiri intervention ([21,24,28]; Multimedia Appendix 1: Supplement A). Students were assigned to groups using a ballot-box procedure.

### About the Shamiri Intervention

Shamiri is a brief, school-based group intervention targeting adolescent depression and anxiety [21,24,28,29]. Its components derive from the science of strengths-based interventions (overlapping with “wise” interventions [43,44]): brief, simple techniques targeting specific psychological processes [45,46]—growth mindset [47,48], gratitude [49,50], and values affirmation [51,52]. Complete protocols are published elsewhere [24-26]; refer to Multimedia Appendix 1: Supplement A.

### Procedures

#### Lay Provider Recruitment, Training, and Supervision

##### Eligibility and Recruitment

Lay providers met the following four criteria: (1) at least aged 18 years; (2) completed secondary school in Kenya with English as the language of instruction; (3) able to read intervention protocols in English; and (4) available for all scheduled sessions [25,28]. We recruited participants openly through WhatsApp (Meta) groups, university forums, and online job boards. Candidates completed online applications and structured 30-minute interviews assessing interest, relevant experience, personal characteristics conducive to group leadership, and responses to hypothetical implementation scenarios [25,28]. Of the applicant pool, 64 were assigned to the Ngong Hub and were eligible for this study.

##### Training

Training followed the standard Shamiri protocol [25,27] over 2 days and covering counseling techniques, group leadership, emergency and risk management, intervention didactics, and extensive role-playing, including approximately 6 hours of practice leading sessions while receiving real-time feedback from supervisors and peers [25]. All lay providers were evaluated using a standardized rubric assessing content accuracy, timing, and subjective quality. Those who scored below standard received additional practice and follow-up assessment before leading groups. The protocol has been published elsewhere [25] and has been used across multiple Shamiri trials and implementations [21,22,28,29].

##### Supervision

Each lay provider was assigned to a clinical supervisor and attended weekly 1-hour supervision sessions structured around reflection, feedback, and peer learning [25,26] (refer to Multimedia Appendix 1: Supplement B).

### Clinical Supervisor Recruitment, Training, and Supervision

Supervisors recruited, trained, and supervised lay providers while ensuring fidelity, managed clinically elevated cases, and triaged emergencies to the Clinical Network of expert psychiatrists and psychologists [25-27]. All held at least a bachelor’s degree in clinical or counseling psychology and were registered with the Kenya Counselors and Psychologists Board. Each supervised approximately 10 lay providers (1:10). Recruitment used a written interview and 2 case interviews with the Shamiri Clinical Team [25,26]. Supervisors then completed approximately 3 months of training covering the intervention manual and session-by-session protocols; clinical supervision techniques, including structured feedback delivery, fidelity rating using the 6-domain instrument, and calibration exercises; group facilitation and peer-counseling skills; identification and triage of elevated cases through the Clinical Network; ethical conduct and professional boundaries; and emergency risk management via the validated Shamiri Risk Management Protocol [25-27]. The Shamiri Risk Management Protocol uses a 3-tier model (lay provider, caseworker or supervisor, and clinical expert) with standardized procedures for recognizing distress, initiating referrals, managing sensitive disclosures, and maintaining boundaries [26]. Supervisors were also trained in data entry and quality control using internal software [53].

### Study Conditions

#### Standard Supervision

All lay providers received weekly supervision from clinical supervisors [25,26]. Supervisors held weekly one-to-one or small-group sessions (~60 min) reviewing experiences and challenges, discussing selected sessions based on self-report and available notes, reinforcing adherence to the manual, and coaching on group management, risk management, and self-care [25,26]. Supervisors occasionally requested audio recordings, but systematic listening to and rating of entire sessions was not feasible given time constraints.

#### AI-Augmented Supervision (ShamiriAI)

Supervisors continued all standard activities but additionally received structured shamiriAI feedback reports for recorded sessions, which they reviewed before or during supervision to guide discussions. Lay providers were not directly exposed to the AI system; all AI-generated feedback was delivered to supervisors, who relayed it during supervision.

### Audio Recording Procedures

Lay providers recorded group sessions using study-supplied digital voice recorders, which captured wide-angle audio in open or semiopen spaces (eg, classrooms, halls, and outdoor areas), producing variable background noise. Recording coverage varied across sessions due to device failures, environmental noise, and school-specific restrictions. We included recordings from all 52 delivered sessions for which a recording was available (100% of recorded sessions). In the AI-augmented arm, Hub Coordinators uploaded files to secure cloud storage after each session; in the standard arm, recordings were archived for potential later analysis but were not processed by shamiriAI during the trial.

### Development of shamiriAI

#### Overview

shamiriAI enhances fidelity monitoring and supervision of lay-delivered group interventions. It processes raw session audio and produces structured feedback reports for clinical supervisors, covering both content- and process-related aspects of delivery. It operates in a code-switched multilingual environment (English, Kiswahili, and Sheng) characteristic of Kenyan secondary schools. It is a clinician-facing decision-support tool in which supervisors remain the primary decision-makers, and AI-generated feedback is one structured input alongside their direct clinical knowledge.

shamiriAI follows a linear 5-stage pipeline: (1) ingestion and preprocessing, (2) core processing (ASR and prosodic feature extraction, performed in parallel), (3) postprocessing and personally identifiable information (PII) scrubbing, (4) LLM-based feedback inference, and (5) delivery. Table 1 summarizes each stage. All processing occurred on secure servers with restricted access.

**Table 1.** Overview of the 5-stage shamiriAI pipeline. Stages 2a and 2b run in parallel on preprocessed audio. The pipeline ingests raw session audio and produces a structured fidelity feedback report for clinical supervisors.

Stage	Inputs	Outputs	Main processes	Key tools
1. Ingestion and preprocessing	Session audio (.wav, .mp3)	Preprocessed audio segments	Format normalization (16 kHz, mono); denoising and spectral gating; VAD^a^	librosa, pydub, webrtcvad
2a. Automatic speech recognition	Preprocessed audio segments	Timestamped transcripts with speaker IDs	Fine-tuned Whisper (small, ~242M params); multilingual automatic speech recognition (English-Kiswahili-Sheng); speaker diarization (local labels); hallucination detection via VAD metadata	Whisper (fine-tuned), pyannote.audio
2b. Prosodic feature extraction	Preprocessed audio segments	Session- and segment-level feature vectors	Spectral features (centroid, bandwidth, contrast); speech-segment analysis (turns, gaps, and exchange ratio); voice diversity (pitch, energy variation); temporal dynamics and voice-quality indicators	librosa, parselmouth, python
3. Postprocessing and PII^b^ scrubbing	Raw transcripts from 2a	Privacy-protected transcripts	NER^c^ for PII^b^ detection; masking of names, schools, locations; secure storage of unredacted audio or transcripts	NuNerZero (numind/NuNerZero); dslim/bert-base-NER^d^ (fallback)
4. LLM^e^-based feedback inference	Redacted transcripts + prosodic features	Structured feedback reports	Structured system prompt with intervention context; integration of transcript and prosodic summaries; fidelity assessment across 6 domains; iterative prompt refinement via clinical review	Gemini 2.5 Pro; custom prompt templates
5. Feedback delivery	Structured feedback from Stage 4	Supervisor-facing PDF reports	PDF generation with session metadata; quantitative prosodic summaries; narrative feedback (strengths, improvements, and suggestions); secure delivery via email/shared folder	ReportLab; secure cloud storage

a VAD: voice activity detection.

b PII: personally identifiable information.

c ER: entity recognition.

d NER: named entity recognition.

e LLM: large language model.

#### Data Sources

The primary inputs were group-session audio recordings (.wav or .mp3) recorded across multiple Shamiri Hub sites at three native sampling rates: 22,050 Hz (43.2%), 44,100 Hz (22.7%), and 48,000 Hz (34.1%). All audio was loaded for feature extraction at its native sampling rate without resampling; consequently, spectral features, mel-frequency cepstral coefficients (MFCCs), and pitch-tracking outputs were computed at the native rate per file and were not directly comparable across sessions recorded at different rates without normalization. ASR training and evaluation used a curated corpus of historical Shamiri recordings from 2023 to 2024, comprising approximately 10 hours across multiple sessions, with reference transcripts prepared by bilingual annotators familiar with Kenyan adolescent speech, capturing English, Kiswahili, and Sheng as spoken. The corpus was split into training (8 h), validation (1 h), and held-out test (1 h) sets. All ASR performance metrics reported in the Results section were based exclusively on the held-out test set. The 52 fidelity-validation sessions comprised 19 Session 1 (Growth Mindset I), 15 Session 2 (Growth Mindset II), 12 Session 3 (Gratitude), and 6 Session 4 (Values Affirmation) recordings.

#### Stage 1: Ingestion and Preprocessing

Hub Coordinators uploaded recordings to secure cloud storage after each session, and a scheduled server-based pipeline retrieved new files. Preprocessing comprised (1) format normalization to 16-kHz mono for consistent downstream input, (2) denoising via spectral gating and noise reduction to mitigate background noise in open environments, and (3) voice activity detection (VAD) to segment audio into speech and nonspeech regions, reducing computation and capturing conversational structure.

#### Stage 2: Core Processing

Stage 2 consisted of the following two major branches executed in parallel: (1) multilingual ASR with speaker diarization and (2) prosodic feature extraction.

##### Stage 2a. Multilingual ASR

###### Model Selection and Architecture

We used an open-source Whisper-based ASR model as the transcription backbone [54], selected for three reasons: strong multilingual performance, including Kiswahili; open-source licensing; and on-premise deployability, which allowed us to avoid transmitting sensitive school recordings to third-party servers. We started from the Whisper Small checkpoint (~242 million parameters), which balances accuracy and computational efficiency for our deployment context.

###### Fine-Tuning

The base model was fine-tuned on the 8-hour training split to improve performance on code-switched Kenyan adolescent speech using a learning rate of 1×10⁻⁵, batch size of 16 per device, and 5000 training steps, with data augmentation (time masking and additive noise) to improve robustness to recording-quality variation. A separate diarization module identified speaker change points and assigned local labels (eg, “Speaker 1” and “Speaker 2”), enabling per-speaker analytics such as talk-time distribution. VAD metadata were attached to each diarized segment to flag silence and identify potential hallucinations (output produced despite no detected voice activity).

###### Computational Resources

Fine-tuning ran on a single NVIDIA Tesla T4 GPU (16 GB) using Google Colab in approximately 11 hours; the checkpoint was finalized on May 26, 2025. Inference over the held-out evaluation set completed in approximately 68 minutes on the same GPU. All other local processing, including audio preprocessing, prosodic feature extraction, and named entity recognition (NER)-based PII redaction, ran on a consumer laptop without dedicated GPU. LLM fidelity scoring used the hosted Google Gemini 2.5 Pro API (gemini-2.5-pro) and required no local GPU. All model development and inference were conducted between May 26, 2025 (fine-tuning completion) and July 15, 2025 (final inference run).

###### Transcription Metrics

Because sessions involve code-switching and agglutinative morphology, particularly in Kiswahili, we evaluated ASR using word error rate (WER), character error rate (CER), and semantic similarity (cosine similarity between multilingual sentence embeddings) [55,56] (refer to Measures for definitions).

###### Zero-Shot Baseline

To contextualize the fine-tuned Whisper Small model, we report zero-shot baseline figures from the published literature rather than running a within-corpus zero-shot evaluation. Zero-shot Whisper Medium performance on Kiswahili is not directly reported in the original release [54] but scales predictably with per-language pretraining volume. Published Swahili fine-tuning work reports zero-shot WER of 0.51‐0.60 on read speech before domain adaptation [57], and code-switched WER is expected to be substantially higher [58].

###### Speaker Diarization

Diarization used pyannote/speaker-diarization-3.1 (pyannote.ai) [59,60], a fully automatic neural pipeline requiring no manual VAD, speaker count, or dataset-specific fine-tuning. It ingests mono 16-kHz audio, downmixing and resampling as needed. No domain adaptation or hyperparameter tuning was applied. Absent manually annotated reference diarizations for the 52 sessions, session-level diarization error rate (DER) was not computed on the Shamiri corpus. Published benchmark DER values for this pipeline across nine standard corpora (AISHELL-4, AliMeeting, AMI, AVA-AVD, DIHARD 3, MSDWild, REPERE, and VoxConverse) range from 7.8% to 50%, depending on recording conditions, with multispeaker meeting benchmarks (AMI and DIHARD 3) suggesting a plausible expected DER of 18%‐22%.

### Stage 2b: Prosodic and Conversational Feature Extraction

In parallel with ASR, we extracted prosodic and conversational features using deterministic signal-processing techniques in librosa and related libraries. These features capture dimensions of group dynamics and facilitator behavior not reflected in transcribed text and were supplied as supplementary inputs to LLM inference [61-63].

#### Spectral Features

Spectral centroid (frequency “center of mass”; vocal brightness/energy), spectral bandwidth (spread of the frequency distribution; articulation clarity), and spectral contrast (expressiveness/vocal variation) [61,64].

#### Speech-Segment Features

Number of speech segments (turns), mean and SD of segment durations, mean and SD of interspeaker gaps, and quick-exchange ratio (proportion of gaps below a short-gap threshold), which served as proxies for interaction quality, equitable participation, pacing, and flow [61,62].

#### Voice Diversity

Pitch diversity (variation in fundamental frequency across speakers and time) and energy diversity (variation in vocal intensity); higher values may indicate distributed rather than facilitator-dominated participation [65].

#### Conversation-Flow Indicators

Speech density (speech vs. silence within sliding windows), MFCCs (timbral characteristics), and rhythm features (tempo and periodicity), which relate to session intensity and pacing [66,67].

#### Temporal Dynamics

Trajectories of pitch and energy across the session and shifts in turn-taking over time, characterizing the therapeutic arc (opening, working phase, and closure) and moments of notable change in group energy [63,64].

#### Voice-Quality Indicators

Zero-crossing rate, harmonic-to-noise ratio, and approximations of jitter and shimmer, which may reflect vocal tension or emotional states signaling group discomfort or facilitator stress [68,69].

All features were computed at both segment and session level and normalized across sessions to support within- and between-lay provider comparisons.

### Stage 3: Postprocessing and PII Scrubbing

Before transcripts reached the LLM, PII scrubbing used NuNerZero (numind/NuNerZero; NuMind), a zero-shot named entity recognition model based on the GLiNER architecture [70]. NuNerZero is a compact bidirectional token classifier trained on the NuNER (version 2.0) dataset, selected for its zero-shot capability (ie, requiring no labeled target-domain data) and support for arbitrary entity-label prompting at inference without fine-tuning. Three entity types (person, location, and organization) were targeted, and detected entities were replaced in place with typed placeholders ([REDACTED_PERSON], [REDACTED_LOCATION], and [REDACTED_ORGANIZATION]). Redaction was applied to ASR transcripts before any content reached LLM inference; audio and unredacted transcripts were retained on secure servers with restricted access. An English BERT (Bidirectional Encoder Representations from Transformers)–based NER model (dslim/bert-base-NER) was implemented as a fallback but not used in production. Formal precision and recall for the redaction pipeline were not computed in this pilot study.

### Stage 4: LLM-Based Feedback Inference

Structured feedback reports were generated using Google Gemini 2.5 Pro (gemini-2.5-pro), selected after benchmarking against several contemporary LLMs, including GPT-based models, because of its superior performance on code-switched transcripts. The model was invoked once per session, receiving the diarized transcript and session-level audio features as structured JSON. Generation hyperparameters were fixed identically across all 52 sessions (Multimedia Appendix 1: Supplement C8): temperature of 1.0, top-p of 0.95, top-k of 40, maximum output of 65,536 tokens, and a dynamic thinking budget. The model returned a strictly typed JSON object validated against a Pydantic schema, which enforced integer scores on the 1‐7 scale for each rubric dimension and prevented continuous or out-of-range values. No postprocessing or regex extraction was applied; scores were obtained directly from the validated object.

Because no random seed was fixed, generation was stochastic, though score-level variance across calls was bounded by the discrete 1‐7 schema. To confirm this empirically, we ran a test-retest analysis on all 52 sessions by running the production prompt 3 times per session and computing the intraclass correlation coefficient (ICC [3,1]) across runs for each dimension. Test-retest ICC values ranged from 0.44 (Q6: Protocol Boundaries) to 0.74 (Q5: Clarity and Accessibility), with an overall composite ICC of 0.71, comparable to the human-human interrater reliability observed in this dataset (refer to Multimedia Appendix 1: Supplement C8.3 for full methods and results).

The structured system prompt (Multimedia Appendix 1: Supplement C9) provided (1) background on the Shamiri intervention, its session-by-session structure, and supervision goals; (2) the six fidelity domains (Required Contents, Specifics, Thoroughness, Clarity, Skill, and Purity), including the scoring rubric and behavioral anchors; (3) the redacted transcript and a session-level audio-features object; and (4) a request for structured feedback, including numerical ratings per domain, specific strengths, areas for improvement with transcript evidence, and suggested focus areas for the upcoming supervision session.

Prosodic features were passed as a raw JSON object with original numeric values (eg, spectral_centroid_mean: 2847.3; speech_gap_mean: 1.42); no bucketing, z-scoring, or label substitution was applied before prompt construction. The prompt provided an interpretation guide mapping each technical field to a plain-language description (eg, quick_exchange_ratio → “conversation flow and turn-taking patterns”; pitch_diversity → “emotional expressiveness and vocal variation”), instructing the model to use these descriptions rather than raw field names in its output. Per-segment prosodic features were embedded within each transcript turn object, keeping the acoustic signal colocated with the corresponding speech text in the model context. Whether prosodic features measurably improve fidelity scoring beyond transcript content alone was not isolated in this pilot and remains to be established.

The system prompt was developed iteratively across 3 versions using a development corpus of earlier Shamiri Hub recordings [21,28,29], distinct from and nonoverlapping with the 52 validation sessions. Refinements were driven by qualitative review of model outputs on this corpus and by progressively closer alignment with the Shamiri Intervention Protocol rubric. The final prompt was locked before assembly and scoring of the validation dataset; no validation session was reviewed, scored, or consulted during prompt development. The 52 sessions therefore constituted a clean held-out evaluation of the locked prompt, with no leakage between development and validation. Prompt-development artifacts were committed to version control together at project handoff rather than incrementally; therefore, file-level timestamps do not individually reflect chronological order, and the provenance account rests on author attestation rather than automated artifact evidence.

### Stage 5: Delivery of Feedback to Supervisors

For each session, shamiriAI generated a PDF report containing session metadata (lay provider ID, school, session number, date, and duration), a quantitative summary of key prosodic indicators (eg, talk-time distribution, speech density, and quick-exchange ratio), numerical ratings per fidelity domain, and a narrative section structured around strengths, areas for improvement, and suggested focus areas. Reports were delivered by secure email or through a shared folder and reviewed during weekly supervision. Supervisors were encouraged to interpret AI-generated ratings and feedback alongside their own knowledge of lay providers and clinical judgment.

### Deployment and Integration

During the study, transcription and feedback generation were orchestrated via a Python pipeline, with human operators managing uploads and report distribution. Longer-term plans include integration into the Shamiri digital infrastructure (shamiriOS; Shamiri Institute) for automated ingestion, background processing, and in-platform feedback dashboards [53].

### Measures

#### Transcription Performance

The primary source for ASR evaluation was a held-out test set of 10 sessions (~1 h) with manually prepared reference transcripts. Transcripts were prepared by bilingual annotators familiar with Kenyan adolescent speech, capturing code-switching among English, Kiswahili, and Sheng. Annotators followed a standardized protocol and resolved disagreements by consensus.

CER measures the Levenshtein edit distance at the character level, normalized by the total number of reference characters. CER was the primary error-rate metric because it is less sensitive to morphological variation than WER. For example, a single incorrect Kiswahili suffix counts as one character error but a full word error under WER, artificially inflating the latter [55,56,71].

WER is the standard benchmark, calculations as (insertions + deletions + substitutions) ÷ total reference words, and was reported as a secondary metric for comparability with published systems, with the caveat that it overpenalizes morphological variation in code-switched speech [56].

Semantic similarity was assessed via cosine similarity between sentence-level embeddings from a multilingual model (LaBSE), capturing whether meaning was preserved despite orthographic variation [72]. Recall-Oriented Understudy for Gisting Evaluation–Longest Common Subsequence, as defined as the longest common subsequence between hypothesis and reference texts, was also computed [73,74]. String similarity metrics, including Jaccard (token overlap) and Jaro-Winkler (character-level near-miss matching), were computed per segment and summarized as means and SDs. These metrics are well suited to the near-misspellings common in Sheng, where spelling is informal and variable [56].

CER and semantic similarity were treated as the primary indicators and were used to monitor improvements during development. WER was reported for comparability. All metrics were computed using the held-out test set only.

#### Fidelity Rating

Intervention fidelity was rated using a structured 6-domain instrument developed and used in prior Shamiri trials and routine quality monitoring [21,25,28,29]. Each domain was rated on a 1‐7 scale (1=poor and 7=excellent):

- Required Contents: the degree to which mandatory session elements (ie, those specified as nonnegotiable in the protocol, including the confidentiality explanation with the appropriate exception clause and required therapeutic techniques) were present and correctly executed.
- Specifics: adherence to the content prescribed for that session number (ie, correct topic, activities, and booklet pages), independent of how thoroughly or skillfully it was delivered.
- Thoroughness: depth and completeness of coverage, including whether the fellow allowed adequate time for reflection and discussion rather than rushing.
- Clarity: whether content was communicated clearly, accessibly, and age-appropriately for secondary school students, including effective language mixing (English and Kiswahili or Sheng), analogies, and examples.
- Skill: the fellow’s use of protocol-specified facilitation techniques, including open-ended questioning, validation, rephrasing in students’ own words, connecting student experiences, and managing group dynamics.
- Purity: the absence of content outside the Shamiri protocol, including off-topic discussion, unsolicited personal disclosures, and concepts not included in the manual.

For each of the 52 sessions, shamiriAI generated automated scores across all 6 domains via its LLM pipeline (Stage 4). Separately, 2 independent human raters rated each session. To prevent contamination, all human ratings were conducted by trained Shamiri supervisors drawn exclusively from hubs other than Ngong Hub; none had been assigned to the AI-augmented arm or had received or reviewed any shamiriAI feedback before or during rating. Raters were blind to AI scores and to each other’s ratings. Each session’s 2 human scores were averaged into a composite, which served as the human reference standard in all primary reliability analyses.

### Statistical Analyses

#### Overview

Two sets of analyses aligned with the 2 pilot aims. Primary analyses were conducted in Python (version 3.12, Python Software Foundation; *pandas*, *SciPy*, and *pingouin*); Gwet AC2 with ordinal weights was computed via the *irrCAC* package, with categories inferred from observed values per the R *irrCAC* default to ensure cross-platform reproducibility. All analysis code is publicly available on the Open Science Framework. All 52 sessions had complete AI and human composite ratings across all 6 dimensions; no imputation or case exclusion was required.

#### Transcription Performance

ASR was evaluated on the held-out test set using the multimetric approach described in the Measures section above (under Transcription Metrics). Segment-level metrics (Jaccard and Jaro-Winkler) are reported as means and SDs across segments. CER and semantic similarity are reported as primary indicators, with WER reported as a secondary comparability metric.

#### Interrater Reliability

Primary analyses compared AI ratings against the human composite by computing the following for each dimension:

1. ICC: a 2-way random-effects, single-measures, absolute-agreement ICC estimated with pingouin [75], treating both raters as random effects to permit generalization beyond the specific raters. ICCs are reported with 95% CIs and interpreted using established benchmarks: poor (<0.50), moderate (0.50‐0.75), good (0.75‐0.90), excellent (>0.90) [76,77].
2. Agreement categories: per dimension, the percentage of sessions in exact agreement (difference=0), adjacent agreement (|difference|≤1), and discrepant (|difference|>1).
3. Bland-Altman analysis: per dimension, plots of the difference (AI minus human composite) against the mean of the 2 methods, with mean difference (systematic bias) and 95% limits of agreement (SD 1.96), allowing visual inspection of directional bias and ranges of stronger or weaker agreement [78].
4. Dimension-level bias: for each of the 6 dimensions and an Average Fidelity composite (mean of all 6 per session), we report means and SDs separately for AI and human composite. To test systematic differences, we conducted 2-tailed paired-sample *t* tests (*α*=.05) comparing AI against the human composite on each dimension and on Average Fidelity (7 tests), with Holm-Bonferroni correction across the 7 comparisons [79]. We report mean differences (AI minus human composite), 95% CIs, *t* statistics, Holm-corrected *P* values, and Cohen *d*. Cohen *d* used the pooled SD of both methods rather than the SD of paired differences to express systematic bias relative to the natural variance of each measurement approach [80]. Positive differences indicate that AI rated higher than the human composite; negative differences indicate lower ratings.

#### Sensitivity Analyses

##### Gwet AC2

The shamiriAI model returns integer 1‐7 scores per dimension via schema-enforced output (Stage 4), while the human reference is the continuous mean of 2 raters. Our primary analysis uses the ICC (2-way random-effects, single-measures, absolute agreement) against the continuous human composite (Formulation B) consistent with conventional practice for agreement against continuous reference data. To address the asymmetry between the AI’s discrete output and the continuous human reference, we added 2 sensitivity analyses using Gwet AC2 with ordinal weights (appropriate for ordinal ratings and robust to ceiling-concentrated distributions):

- Formulation A: AI vs each individual human rater—Gwet AC2 between AI integer scores and each rater’s integer scores, preserving the discrete structure of both sources and avoiding compositing of the human reference.
- Formulation C: AI vs rounded human composite—Gwet AC2 between AI integer scores and the human composite rounded to the nearest integer, matching the AI’s discrete scale to a discretized reference.

Dimension-level findings are interpreted as robust to the discrete-vs-continuous asymmetry if the ordering and sign of agreement are consistent between Formulation B (primary) and Formulations A and C (sensitivity). Full results across all 3 formulations appear in Multimedia Appendix 1: Supplement E2.

##### Demographic Subgroup Analyses

To examine whether AI-human bias varied by lay-provider demographics, we stratified the per-session AI-minus-human-composite difference by sex (female/male) and fellow age band (median split at 20 years). Welch 2-sample *t* tests on the difference were conducted separately for each dimension, and Gwet AC2 (ordinal weights, against the rounded human composite) was computed within each stratum to characterize within-stratum agreement. These tests were exploratory, were not multiplicity corrected, and are reported descriptively rather than as confirmatory tests.

##### Per-Arm Robustness Check

Because the 52 sessions were unevenly distributed between the AI-augmented (n=38) and standard (n=14) arms, we compared AI rating means, human composite means, and per-arm Gwet AC2 between arms for each dimension. Welch 2-sample *t* tests assessed between-arm differences in AI and human composite ratings; per-arm AC2 (ordinal weights, against the rounded human composite) characterized whether the dimension-level agreement pattern held within each arm. As with the subgroup analyses, these are exploratory and reported descriptively.

### Ethical Considerations

The study was approved by the Daystar University Institutional Scientific and Ethics Review Committee (DUISERC; approval DU-ISERC/10/06/2025/000015E; June 10, 2025) and licensed by the National Commission for Science, Technology and Innovation (NACOSTI; license NACOSTI/P/25/415077; issued February 11, 2025; valid through February 11, 2026). The trial was registered with the Pan African Clinical Trials Registry (PACTR202508900479778) on August 1, 2025. Audio recording at Ngong Hub began in May 2025, while routine program delivery operated under 2 existing approvals: the Kenyatta University Ethics Review Committee renewal (KUERC; application PKU/2627/E1752; renewed November 12, 2024; valid through November 12, 2025) for the broader “Testing Pathways to Scale for a Cost-Effective and Evidence-Based Mental Health Innovation” study, and the NACOSTI license (NACOSTI/P/25/415077) issued February 11, 2025 [41,42]. The A/B-test–specific DUISERC approval was received on June 10, 2025, and the first shamiriAI feedback reports were delivered to supervisors on June 20, 2025, after that approval was in place. PACTR registration was completed on August 1, 2025, after enrollment commenced; we acknowledge this as retrospective registration, which occurred because of in-house administrative transitions during a period of staff change. All data collection and AI-augmented supervision activities were conducted under existing DUISERC and KUERC approvals throughout. Written informed consent was obtained from all adult lay providers and adult student participants before involvement. For minors, written assent was obtained from the student, alongside parental or guardian consent secured through school administration, in accordance with DUISERC, KUERC, and NACOSTI protocols. Participants were informed that involvement was voluntary and that they could withdraw at any time without consequence. Lay providers were compensated at the standard Shamiri Institute rate of KES 1500 (approximately US $12) per 1-hour session delivered, consistent with their employment terms [25]. Student participants received no compensation. Audio recordings and unredacted transcripts were stored on secure servers with restricted access. Before any AI inference, personally identifiable information (PII), including names, school identifiers, and location references, was detected and masked using a named entity recognition pipeline and replaced with typed placeholders ([REDACTED_PERSON], [REDACTED_LOCATION], and [REDACTED_ORGANIZATION]). Only deidentified, redacted transcripts were used in LLM-based fidelity inference (refer to Stage 3 and Multimedia Appendix 1: Supplement C7). No participants are identified in any image or figure in this paper.

## Results

### Sample Characteristics

**Figure 1.**
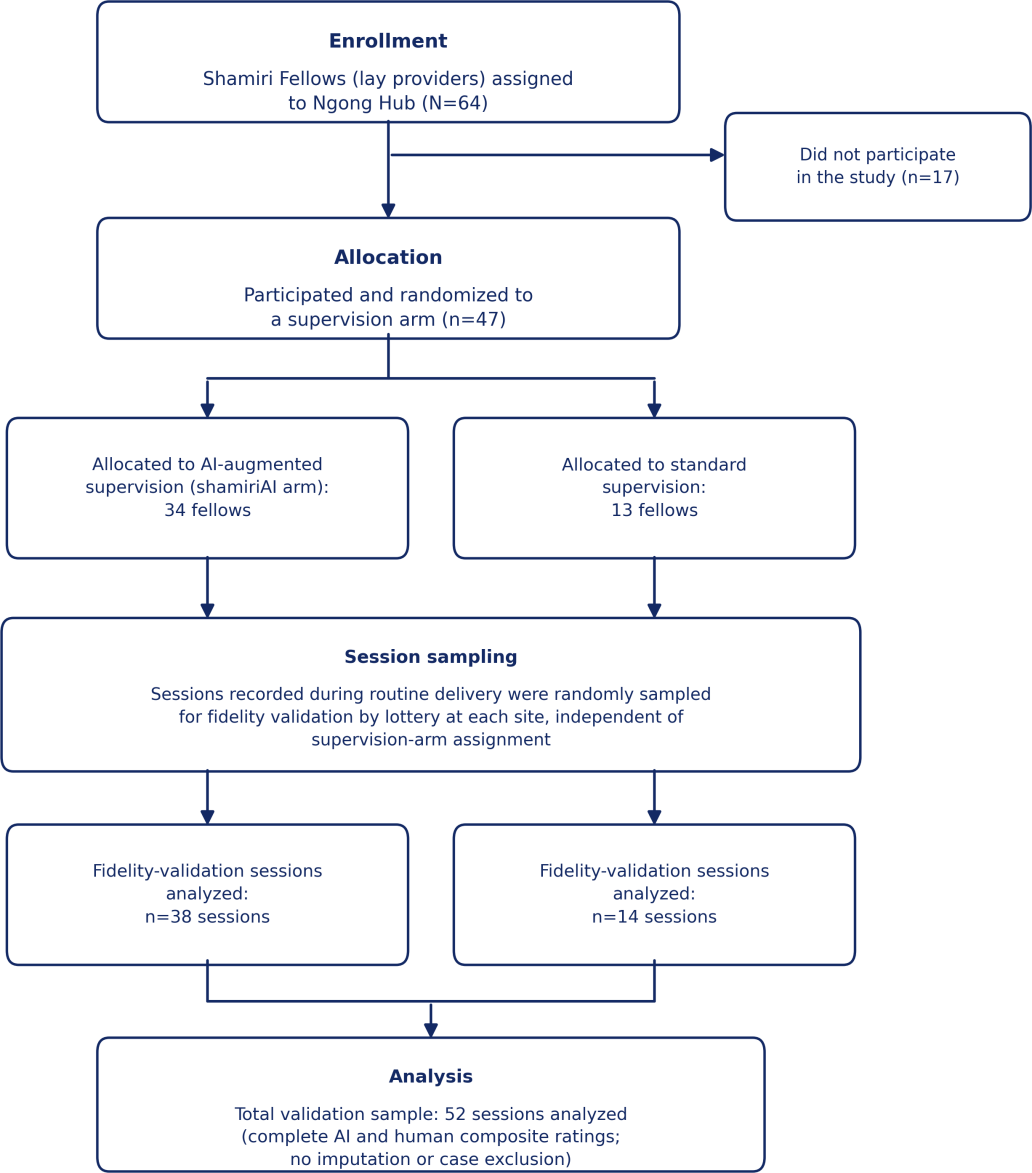
CONSORT (Consolidated Standards of Reporting Trials) diagram showing participant and session flow for the shamiriAI fidelity-validation pilot (conducted across 6 secondary schools at Ngong Hub, Kajiado County, Kenya, from May to September 2025). Lay providers were randomized to supervision arms; sessions were sampled independently of arm assignment for fidelity validation.

Of the 64 Shamiri Fellows assigned to Ngong Hub, 47 participated. Thirty-four were randomized to the AI-augmented supervision arm and 13 to standard supervision (Figure 1; Multimedia Appendix 1: Supplement D1). Most were female (36/47, 76.6%). Fellows ranged in age from 18 to 23 years (mean 19.83, SD 1.65). Across fellows, 52 recorded sessions were included in the fidelity analysis; most fellows contributed 1 or 2 recordings, and most rated sessions were from the AI-augmented arm (38/52, 73%). Seven supervisors participated; most were female (5/7, 71%), ranging in age from 23 to 30 years (mean 25.57, SD 2.23). Five (71%) supervisors were assigned to the AI-augmented arm and two (29%) to standard supervision. Sample characteristics are reported in Multimedia Appendix 1: Supplement D2.

### Aim 1: Transcription Performance

The fine-tuned Whisper model (small checkpoint;~242 million parameters) was evaluated on a held-out test set of 10 sessions with manually prepared reference transcripts. The model achieved a CER of 0.19 and a WER of 0.34. Cosine semantic similarity (LaBSE-based) was 0.77, and Recall-Oriented Understudy for Gisting Evaluation–Longest Common Subsequence was 0.60. Segment-level Jaccard similarity averaged 0.72 (SD 0.26) and Jaro-Winkler similarity averaged 0.78 (SD 0.21). Full ASR metrics are provided in Table 2.

**Table 2.** Automatic speech recognition performance metrics for the fine-tuned Whisper model on the held-out test set (n=10 sessions).

Metric	Value
Error-rate metrics	
Character error rate^a^	0.19
Word error rate^b^	0.34
Semantic similarity metrics	
Cosine semantic similarity (LaBSE)^c^	0.77
ROUGE-L^d^	0.60
String similarity metrics (segment level)	
Jaccard similarity	0.72
Jaro-Winkler similarity	0.78

a Levenshtein edit distance at character level, normalized by total reference characters.

b (insertions + deletions + substitutions) ÷ total reference words.

c LaBSE: Language-agnostic BERT (Bidirectional Encoder Representations from Transformers) Sentence Embedding; cosine similarity computed between sentence-level embeddings of hypothesis and reference transcripts.

d ROUGE-L: Recall-Oriented Understudy for Gisting Evaluation–Longest Common Subsequence variant.

### Aim 2: Fidelity Rating

#### Overview

Fifty-two sessions were rated by both shamiriAI and 2 independent human raters drawn from trained Shamiri supervisors at hubs other than Ngong (refer to the Fidelity Rating subheading in the Methods section). Sessions spanned all four Shamiri session types across six schools: Session 1 (Growth Mindset I; n=19), Session 2 (Growth Mindset II; n=15), Session 3 (Gratitude; n=12), and Session 4 (Values Affirmation; n=6). All 52 sessions had complete AI and human scores across all 6 fidelity dimensions; no ratings were missing.

**Figure 2.**
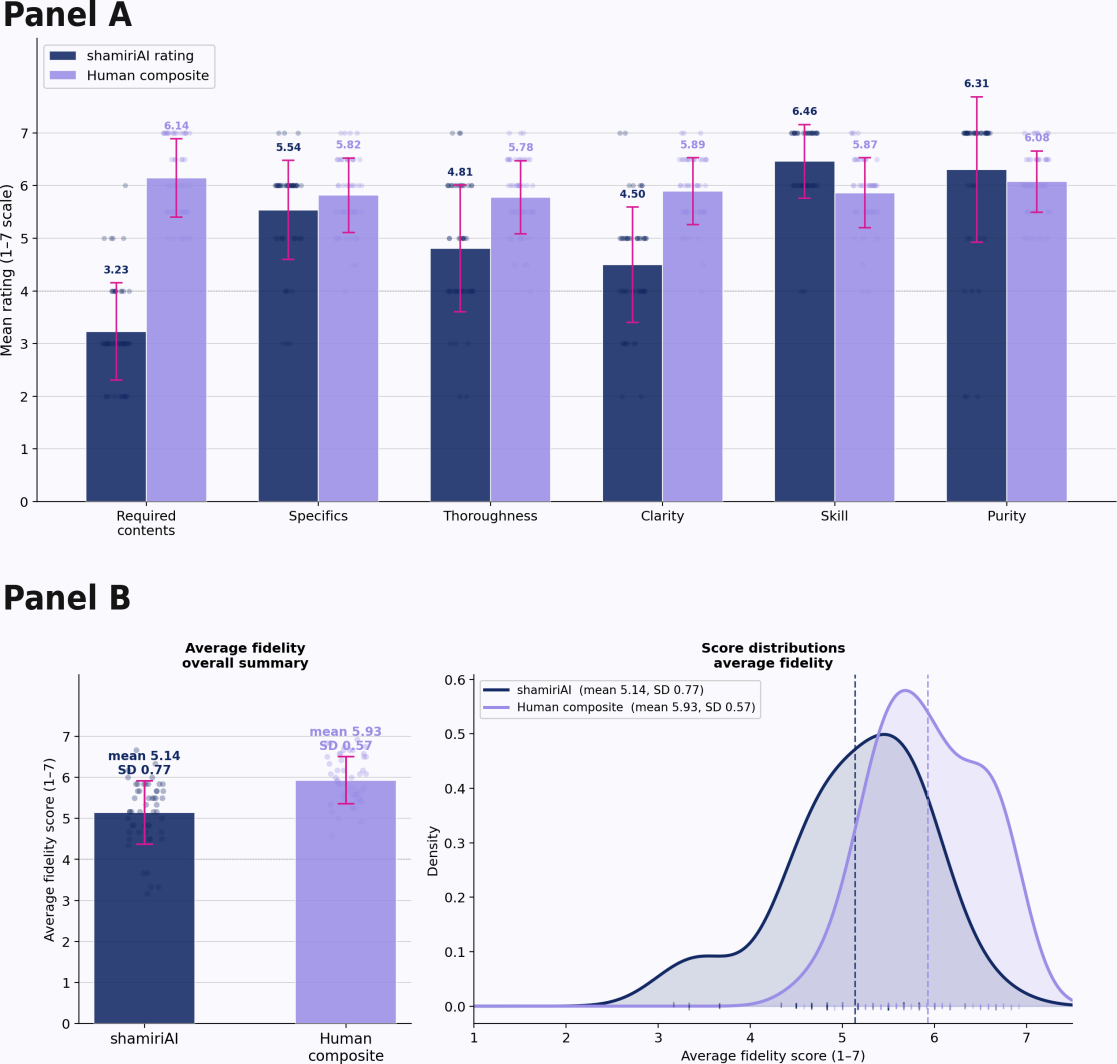
Distribution of shamiriAI vs human-composite mean fidelity ratings across the 6 rubric dimensions (Panel A) and the average fidelity composite (Panel B) (N=52 sessions). Ratings were on a 1‐7 scale (1=poor, 7=excellent); error bars represent ±1 SEM. AI: shamiriAI large language model–generated scores; human composite: mean of 2 independent human supervisor scores.

Table 3 provides descriptive statistics for AI and human composite ratings across all 6 dimensions and the Average Fidelity composite (Panel A in Figure 2). Human composite ratings ranged from mean 5.78 (SD 0.70) for Thoroughness to mean 6.14 (SD 0.74) for Required Contents, with SDs ranging from 0.58 to 0.74 across dimensions. AI ratings spanned a wider range, from mean 3.23 (SD 0.92) for Required Contents to mean 6.46 (SD 0.70) for Skill.

**Table 3.** Descriptive statistics, intraclass correlation coefficients, and agreement rates for AI vs human composite fidelity ratings (N=52).

Dimension	AI ratings^a^, mean (SD)	AI ratings, range	Human composite^b^, mean (SD)	Human composite, range	ICC^c^ (95% CI)	Agreement (%), exact^d^	Agreement (%), cumulative agreement^e^	Agreement (%), discrepant^f^
Required Contents	3.23 (0.92)	2‐6	6.14 (0.74)	5‐7	−0.00 (−0.03 to 0.05)	3.8	5.8	94.2
Specifics	5.54 (0.94)	3‐7	5.82 (0.71)	4‐7	0.03 (−0.23 to 0.29)	13.5	78.8	21.2
Thoroughness	4.81 (1.21)	2‐7	5.78 (0.70)	4‐7	−0.06 (−0.23 to 0.15)	11.5	40.4	59.6
Clarity	4.50 (1.09)	2‐7	5.89 (0.64)	4‐7	0.14 (−0.08 to 0.39)	3.8	40.4	59.6
Skill	6.46 (0.70)	4‐7	5.87 (0.67)	4‐7	0.12 (−0.09 to 0.33)	25.0	71.2	28.8
Purity	6.31 (1.38)	2‐7	6.08 (0.58)	5‐7	0.20 (−0.08 to 0.44)	15.4	73.1	26.9
Average Fidelity^g^	5.14 (0.77)	3.17‐6.67	5.93 (0.57)	4.58‐6.92	—^h^	—	—	—

a AI Ratings: shamiriAI large language model–generated scores.

b Human composite: mean of 2 independent human supervisor scores.

c ICC: intraclass correlation coefficient (2-way random-effects, single-measure, absolute-agreement model).

d Exact: sessions rated identically (difference=0).

e Cumulative agreement: ratings within 1 scale point (|difference|≤1), inclusive of exact agreement.

f Discrepant: ratings differing by >1 scale point (|difference|>1).

g Average Fidelity: mean of all 6 dimension scores per session.

h Not applicable.

At the composite level, AI Average Fidelity (mean 5.14, SD 0.77) was lower than the human composite (mean 5.93, SD 0.57), with a mean difference of −0.79 (95% CI −1.04 to −0.53; *t*_51_=−6.22; *P*<.001; d=−1.16) (Panel B in Figure 2).

#### Intraclass Correlation and Agreement

**Figure 3.**
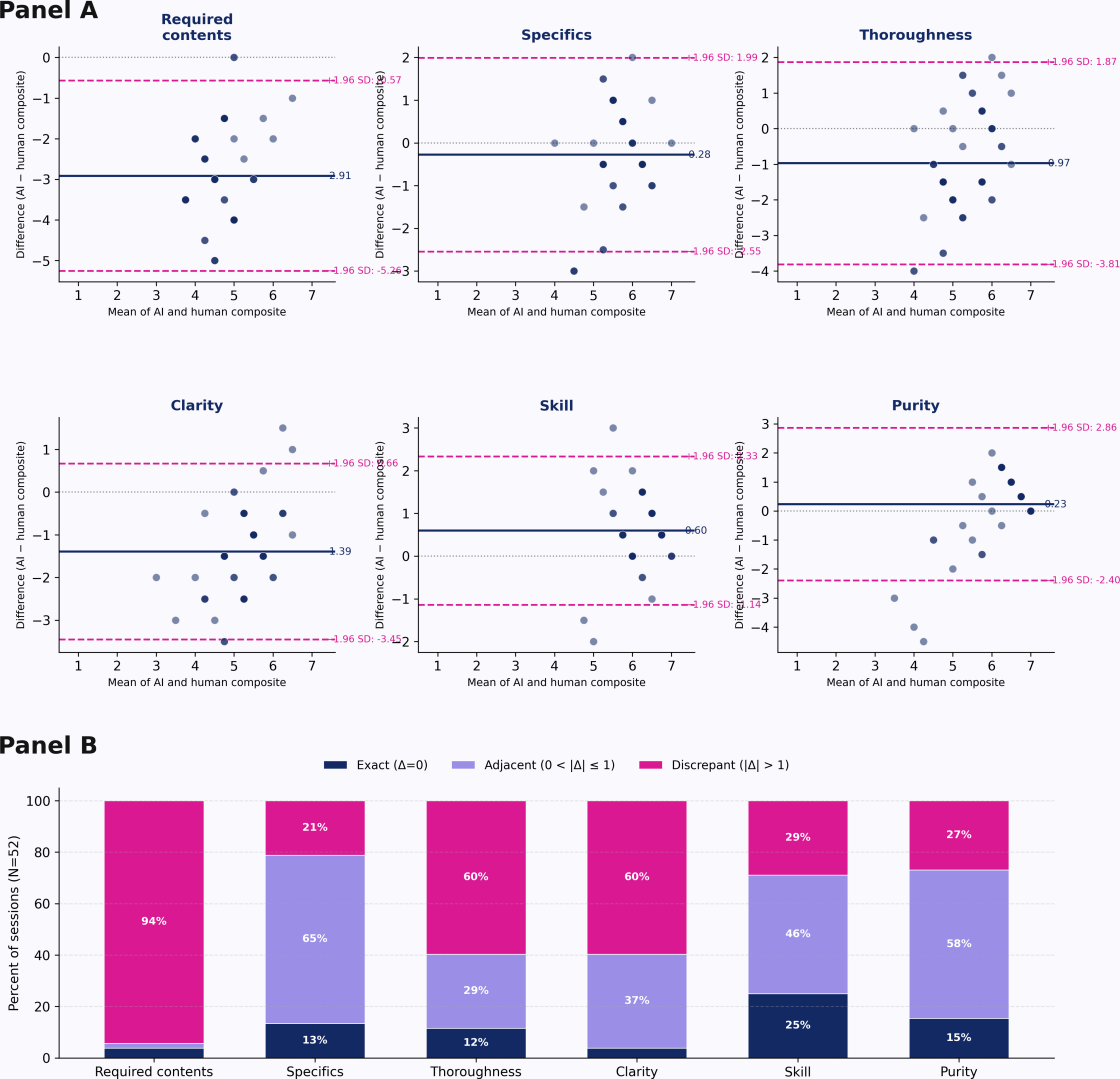
Bland-Altman plots and agreement-category distribution for shamiriAI vs human-composite fidelity ratings (N=52 sessions). (A) Bland-Altman plots for each of the 6 rubric dimensions, plotting the difference (AI minus human composite) against the mean of the 2 rating sources; the solid line indicates mean systematic bias, and dashed lines the 95% limits of agreement (SD 1.96). (B) stacked bar chart showing the percentage of sessions classified as Exact (difference=0), Adjacent (within 1 scale point but not exact), and Discrepant (>1 scale point apart) for each dimension. AI: shamiriAI large language model–generated scores; Human composite: mean of 2 independent human supervisor scores.

ICC estimates for AI vs human composite ratings (Table 3) ranged from −0.06 (Thoroughness; 95% CI −0.23 to 0.15) to 0.20 (Purity; 95% CI −0.08 to 0.44). CIs for all 6 dimensions included 0, and all values fell below 0.50. Bland-Altman plots for all 6 dimensions are provided in Figure 3A. Limits of agreement (SD 1.96) ranged from approximately 2.4 scale points (Skill) to 5.0 scale points (Required Contents).

Adjacent agreement rates (|difference|≤1) varied by dimension: Specifics 78.8%, Purity 73.1%, Skill 71.2%, Thoroughness 40.4%, Clarity 40.4%, and Required Contents 5.8% (Table 3; Panel B in Figure 3). Exact agreement rates ranged from 3.8% (Required Contents and Clarity) to 25% (Skill). Discrepant ratings (|difference|>1) ranged from 21.2% (Specifics) to 94.2% (Required Contents).

#### Systematic Bias: Dimension-Level Analysis

**Figure 4.**
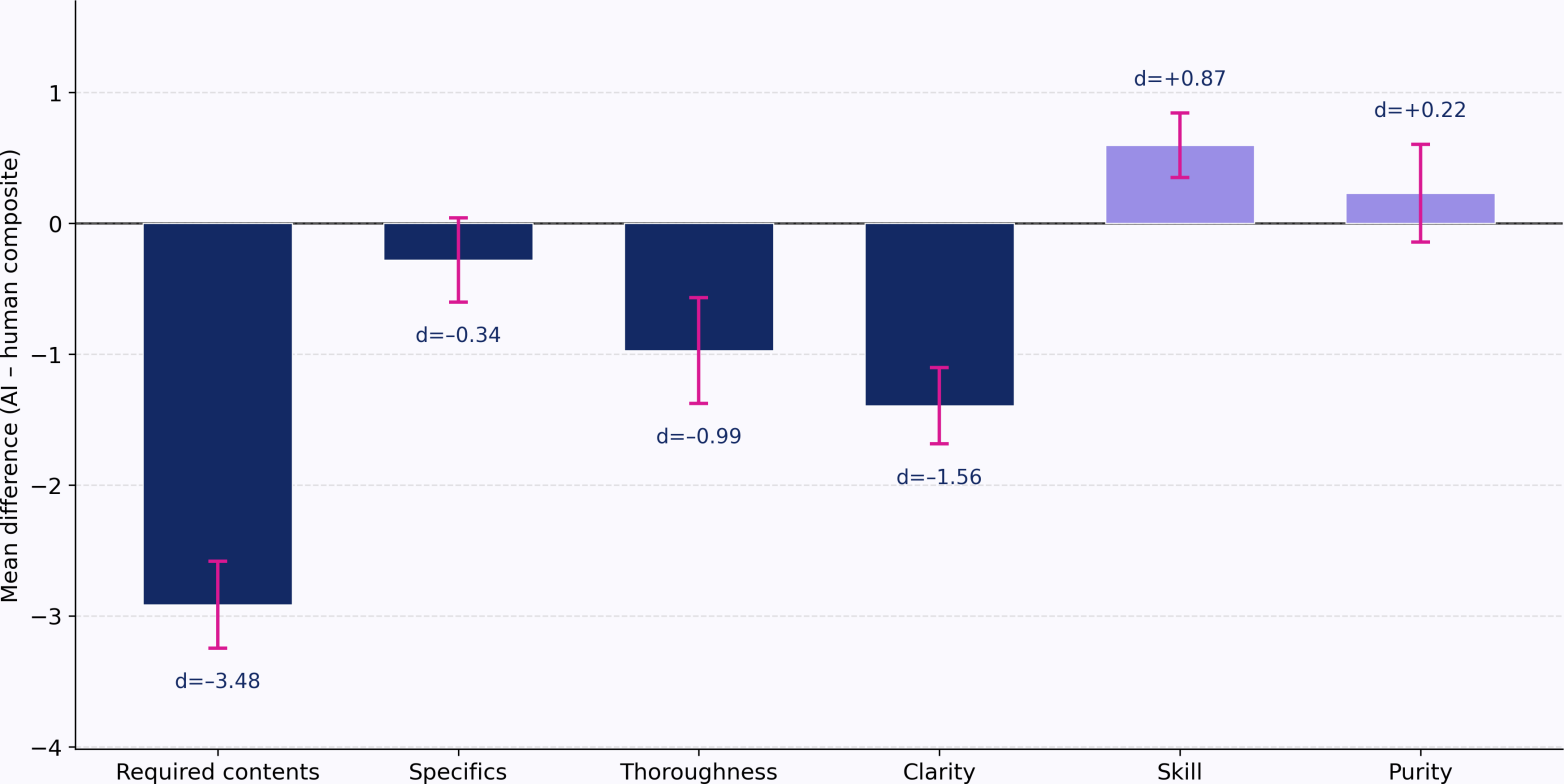
Dimension-level systematic bias between shamiriAI and the human composite (N=52 sessions). The chart plots the mean difference (AI minus human composite) per rubric dimension with 95% CIs, alongside Cohen *d* effect sizes. Negative values indicate AI rated lower than the human composite; positive values indicate that AI rated higher. Effect sizes were computed using the pooled SD of the 2 rating methods. Asterisks indicate dimensions for which systematic bias was statistically significant after Holm-Bonferroni correction across 7 comparisons.

Paired *t* tests comparing AI ratings against the human composite, with Holm-Bonferroni correction across 7 simultaneous comparisons, showed statistically significant systematic differences for 5 of 7 comparisons (Table 4; Figure 4).

**Table 4.** Systematic bias analysis: paired *t* tests comparing AI and human composite fidelity ratings with Holm-Bonferroni correction (N=52). Effect size benchmarks: small |d| 0.20-0.49, medium 0.50-0.79, large ≥0.80. ***P_adj<.001. Mean differences (ΔM) are computed from unrounded session-level scores and equal the mean of the paired (AI − human composite) differences; they may differ from the difference of the rounded means shown by up to 0.01*.*

Dimension	AI, mean (SD)	Human, mean (SD)	ΔM^a^ (95% CI)	*t* (*df*)	*P*_adj^b^	d^c^	Direction
Required Contents	3.23 (0.92)	6.14 (0.74)	−2.91 (−3.25 to −2.58)	−17.58 (51)	<.001	−3.48	Underrates^d^
Specifics	5^d^.54 (0.94)	5.82 (0.71)	−0.28 (−0.60 to 0.04)	−1.74 (51)	.18	−0.34	—^e^
Thoroughness	4.81 (1.21)	5.78 (0.70)	−0.97 (−1.38 to 0.57)	−4.83 (51)	<.001	−0.99	Underrates^d^
Clarity	4.50 (1.09)	5.89 (0.64)	−1.39 (−1.69 to 1.10)	−9.58 (51)	<.001	−1.56	Underrates^d^
Skill	6.46 (0.70)	5.87 (0.67)	0.60 (0.35-0.84)	4.85 (51)	<.001	0.87	Overrates^d^
Purity	6.31 (1.38)	6.08 (0.58)	0.23 (0.14-0.60)	1.24 (51)	.22	0.22	—
Average Fidelity	5.14 (0.77)	5.93 (0.57)	−0.79 (−1.04 to 0.53)	−6.22 (51)	<.001	−1.16	Underrates^d^

a ΔM: AI mean minus human composite mean (positive= AI rated higher; negative=AI rated lower).

b *P*_adj: Holm-Bonferroni-corrected *P *value across seven simultaneous comparisons (6 dimensions + Average Fidelity composite).

c *d*: Cohen *d* (pooled SD method).

d shamiriAI either overrates or underrates human rates.

e Nonsignificant.

AI underrated Required Contents (mean 3.23, SD 0.92 vs mean 6.14, SD 0.74; ΔM=−2.91, 95% CI −3.25 to −2.58; *t*_51_=−17.58; *P*<.001; d=−3.48), with 94.2% of sessions rated discrepantly (|difference|>1). AI also underrated Clarity (ΔM=−1.39, 95% CI −1.69 to −1.10; *t*_51_=−9.58; *P*<.001; d=−1.56) and Thoroughness (ΔM=−0.97, 95% CI −1.38 to −0.57; *t*_51_=−4.83; *P*<.001; d=−0.99). AI overrated Skill (ΔM=+0.60, 95% CI 0.35-0.84; *t*_51_=4.85; *P*<.001; d=+0.87). No statistically significant bias was observed for Specifics (ΔM=−0.28; *P*_adj=.18; d=−0.34) or Purity (ΔM=+0.23; *P*_adj=.22; d=+0.22); these dimensions had the highest adjacent agreement rates (78.8% and 73.1%, respectively).

For the Average Fidelity composite, AI ratings (mean 5.14, SD 0.77) were significantly lower than the human composite (mean 5.93, SD 0.57), with a mean difference of −0.79 (95% CI −1.04 to −0.53; *t*_51_=−6.22; *P*<.001; d=−1.16) (Figure 4).

### Secondary and Sensitivity Analyses

#### Human-Human Agreement

Human-human AC2 values were computed for all 5 rater pairs across 52 sessions. Human-human AC2 ranged from 0.42 (Purity) to 0.60 (Clarity), with all 6 dimensions in the moderate range (0.41‐0.60).

#### AI-Human Agreement

Three formulations of AI-human agreement are reported (refer to Sensitivity Analyses in the Methods section). ICCs between AI ratings and the continuous human composite (Formulation B, primary) ranged from −0.058 (Thoroughness) to 0.196 (Purity), with all 6 dimensions classified as “poor” according to Koo and Li benchmarks [87^81^] (Table 3). Specifics and Purity reached 78.8% and 73.1% adjacent agreement, respectively (Table 3).

Gwet AC2 against each individual human rater (Formulation A, sensitivity) ranged from −0.628 (Required Contents) to 0.764 (Specifics) for Rater 1 and from −0.482 (Required Contents) to 0.645 (Skill) for Rater 2. Gwet AC2 against the rounded human composite (Formulation C, sensitivity) was −0.667 (Required Contents), 0.687 (Specifics), 0.487 (Thoroughness), 0.376 (Clarity), 0.741 (Skill), and 0.762 (Purity). Full results across all 3 formulations are reported in Multimedia Appendix 1: Supplement E2.

AI-human AC2 differed across individual rater pairs for several dimensions. For Thoroughness, AC2 was 0.626 against Rater 1 and 0.360 against Rater 2; for Specifics, the corresponding values were 0.764 and 0.438.

#### Demographic Subgroup Analyses

We computed AI-minus-human composite mean differences and Gwet AC2 stratified by Fellow sex (female: n=39 sessions and male: n=13 sessions) and fellow age band (median split at 20 years; <20 years: n=23 sessions; ≥20 years: n=29 sessions) for each of the 6 dimensions. Welch 2-sample *t* tests on AI-human differences yielded no statistically significant sex effect on any dimension (all *P*>.25) and no statistically significant age-band effects on any dimension (all *P*>.10). Per-stratum AC2 values are reported in Multimedia Appendix 1: Supplement E3 (Tables E3.1 and E3.2).

#### Per-Arm Robustness Check

We compared AI rating means, human composite means, and per-arm Gwet AC2 between the AI-augmented (n=38 sessions) and standard supervision (n=14 sessions) arms for each of the 6 dimensions. AI rating means did not differ significantly between arms on any dimension (all Welch *t* test *P*>.29), and human composite means also did not differ (all *P*>.55). Per-arm AC2 followed the same dimension-dependent pattern as the overall analysis for 5 of 6 dimensions. Clarity AC2 was 0.394 in the AI-augmented arm and 0.034 in the standard arm, while AI rating means and human composite means for Clarity did not differ between arms (all *P*>.61). Full per-arm distributions and AC2 estimates are reported in Multimedia Appendix 1: Supplement E5.

## Discussion

### Principal Findings

This pilot pursued 2 foundational aims for shamiriAI—an automated fidelity-monitoring system for lay-delivered group mental health interventions in multilingual, low-resource settings.

The first aim established that the fine-tuned Whisper pipeline produced transcripts of sufficient semantic quality for downstream fidelity evaluation in naturalistic, multispeaker, code-switched English-Kiswahili-Sheng audio. A WER of 0.34 looks high against English-only benchmarks, but zero-shot Whisper on Kiswahili commonly exceeds 0.50 on read speech [82,83]. After being trained on about 8 hours of domain data and operating on spontaneous group speech, our model represents a meaningful gain at minimal cost. The metrics most appropriate for this context—CER (0.19) and cosine semantic similarity (0.77)—indicate that session meaning is preserved even where individual tokens are misrecognized. The output is semantically coherent enough to support LLM-based inference.

The second aim produced a more complex picture. Across all 6 dimensions, shamiriAI rated sessions an average of 0.79 points lower than the human panel on average—a large aggregate effect (d=−1.16) that remained significant after Holm-Bonferroni correction. The model scored more critically than human supervisors. That headline obscures 3 distinct patterns with different implications for development.

### Pattern 1: Limitations of Holistic Interpretive Dimensions

The largest discrepancies were observed for Required Contents (d=−3.48) and Clarity (d= −1.56). The AI differed from the panel by more than one scale point in 94% of Required Contents sessions, and Clarity adjacent agreement was 40%. Both dimensions require holistic, integrative judgment about whether a session was complete and clear “in spirit,” not whether scripted elements appeared verbatim. Human supervisors credit spirit-compliant delivery—paraphrase, local idiom, and language mixing—as meeting the standard, whereas the AI appears to apply a more literal rubric, penalizing missing scripted phrases [84,85]. Required Contents may have a further diagnosable cause: ASR errors in group audio destroy the short transitional utterances that signal element completion, and required elements are often spread across turns and speakers rather than delivered in one block. These are architectural, not prompt-tuning, problems and point to specific version 2 fixes, including required-element checklists in the prompt, sequential per-element evaluation before an overall rating, behavioral anchors from calibrated annotations, and few-shot examples spanning acceptable delivery styles.

### Pattern 2: Bidirectional Bias on Facilitation Dimensions

Two medium effects ran in opposite directions: the AI underrated Thoroughness (d=−0.99) and overrated Skill (d=+0.87). The opposing signs suggest that the model reads surface markers of facilitation—open questions, reflective language, validation, and restatement—as skill [85], but does not assess whether they were used at adequate depth, at the right moments, and with time for student reflection [86]. A provider producing dense facilitation language while rushing content earns high Skill but low Thoroughness; a supervisor seeing both together rates them more consistently. Prompt redesign should partly correct this by incorporating Thoroughness anchors distinguishing depth from technique frequency and Skill anchors weighting contextual appropriateness alongside behavioral presence, without new training data.

### Pattern 3: Operationally Viable Performance on Structured Detection Dimensions

This is a positive finding. No significant bias was detected for Specifics (d=−0.34; *P*=.18) or Purity (d=+0.22; *P*=.22), and adjacent agreement reached 78.8% and 73.1%, approaching the human–human AC2 ceiling of 0.42‐0.60. Both are detection tasks—whether prescribed content is present and prohibited content was absent—and on these shamiriAI already performs at a level that could support supervisory decisions. This matters because Specifics and Purity are the dimensions most tied to protocol adherence and clinical safety, the quality floor that supervision exists to enforce. An AI system that flags off-protocol sessions and confirms coverage already gives supervisors actionable signals on what matters most for preventing drift [86,87].

### Interpreting the Reliability Indices

The reliability indices must be interpreted relative to 2 intrinsic properties of the human reference standard. First, human composite ratings clustered near the ceiling (mean 5.78‐6.14, SD 0.58‐0.74), restricting variance and mechanically suppressing ICCs regardless of true rank alignment. This explains why Specifics and Purity were classified as “poor” by ICC benchmarks [81,88] yet exceeded 70% adjacent agreement. Gwet AC2, with prevalence- and bias-adjusted chance correction, partly corrects this and confirms the dimension ordering. Second, human-human AC2 ranged from 0.42 to 0.60, representing the realistic upper bound any model could achieve against this particular composite [89]. AI-human AC2 on Specifics, Skill, and Purity approaches that ceiling rather than falling short of an unattainable maximum.

The 52-session sample is small for stable AC2 estimation, and CIs are wide (Tables 3 and 4). Accordingly, these estimates require replication in a planned larger cohort (target N=400). The direction is more secure: the dimension ordering—highest for Specifics, Skill, and Purity; lowest for Required Contents; and intermediate for Thoroughness and Clarity—held across the ICC analysis, both AC2 formulations, and the sex, age, and arm subgroups, suggesting that it is not an artifact of the reference treatment or demographic or arm-level confounding. These subgroup analyses were exploratory and underpowered, excluding only large effects.

### Comparison With Prior Work

Possibly comparable existing systems achieve higher reliability but under far more favorable conditions. Lyssn (Lyssn.io), a commercial platform trained on about 2500 labeled sessions of individual CBT by professional therapists in English, reaches 100% of human reliability on the overall quality score and exceeds 80% on 10 of 11 items [36]. A recent LLM system scored patient engagement across 1131 individual CBT sessions in German with strong reliability and outcome-linked validity [40]. shamiriAI currently achieves lower reliability, but the conditions differ fundamentally: individual vs group, professional vs lay providers, 1 language vs 3 including code-switched Sheng, large labeled corpora vs 8 hours, controlled vs open-environment audio. The relevant benchmark is the gap: on Specifics and Purity, shamiriAI already approaches the range of systems built for easier conditions, and its Required Contents and Clarity limitations are interpretable and addressable rather than fundamental. Procedural prompting has been shown to substantially improve LLM assessment reliability [90], directly applicable to the priorities here.

### Limitations

This pilot has several limitations that constrain inference and set priorities for planned follow-up studies (target N=400) and future development.

First, the 52-sessions sample is small for stable agreement estimation in a ceiling-affected distribution; AC2 CIs are wide, and a larger sample is needed.

Second, the human reference showed only moderate interrater reliability (AC2 0.42‐0.60) and a restricted range (mean 5.78‐6.14, SD 0.58‐0.74), capping achievable agreement. Future iterations should incorporate structured rater calibration training and behavioral-anchored formats.

Third, the validation set was sampled without stratification by arm, producing a 38:14 imbalance. Per-arm comparisons detected no systematic bias, but validation with arm-balanced samples is needed.

Fourth, prompt-development provenance rests on author attestation rather than incremental timestamps, because artifacts were committed together at handoff; all 3 iterations are archived on Open Science Framework. Future development should adopt incremental version control.

Fifth, the PII redaction pipeline was not formally evaluated for precision and recall. Person-name redaction is qualitatively acceptable, but location and organization redaction is plausibly weaker for Sheng and Kiswahili. Accordingly, we treat NER as a supplementary layer behind secure storage and consent.

Sixth, audio recordings spanned 3 native sampling rates without resampling, so spectral features, MFCCs, and pitch outputs are not strictly comparable across sessions. Cross-rate normalization should be added in a v2.

Seventh, the zero-shot ASR baseline is drawn from published benchmarks [54,57,58] rather than a within-corpus run, so the fine-tuning gain cannot be precisely quantified; ASR metrics were also computed on a separate held-out test set, not the 52 fidelity validation sessions.

Eighth, diarization DER was not measured on the Shamiri corpus; we report published benchmark DER for pyannote [59,60] as a proxy, and actual DER in adolescent group sessions may be worse.

Ninth, a prosodic-feature ablation was deferred to the larger cohort, where it can be adequately powered rather than interpreted post hoc.

Tenth, all sessions came from a single hub in Kajiado County; generalizability to other hubs, regions, languages of code-switching, or non-Shamiri interventions must be tested in multisite replication.

Eleventh, the trial was registered with PACTR after enrollment commenced; we acknowledge this departure from prospective registration and note that all activities fell within the DUISERC and KUERC approval period.

Twelfth, raters were calibrated before coding but did not recalibrate during the rating window and could not see one another’s scores, so within-trial drift cannot be fully excluded.

Thirteenth, Session 4 was underrepresented (6/52, 11.5%), so its reliability should be reestimated in the larger cohort.

Fourteenth, scores come from a generative model sampled at temperature 1.0, so identical inputs can vary slightly across runs; test-retest agreement across 3 runs was high, and deterministic scoring would require temperature 0 or aggregation across runs.

Finally, this study evaluates technical performance only. Whether AI-augmented supervision improves provider skill, fidelity at scale, or student outcomes remains for the planned cluster-randomized noninferiority trial.

### Future Directions: shamiriAI in the Broader Vision

The goal of shamiriAI is not fidelity monitoring for its own sake but a quality-assurance infrastructure that can support lay-provider supervision at population scale—the precondition for task-shifted care to reach the millions of young people who receive no help [18,33,35,91,92]. This pilot establishes the 2 foundations required: a transcription pipeline that works in multilingual, code-switched, naturalistic group speech, and a fidelity-rating system whose failures are diagnosable rather than diffuse.

The immediate priority is shamiriAI (version 2)—improved ASR through expanded fine-tuning, prompt redesign for Required Contents, Clarity, and Thoroughness, and integration into shamiriOS [53] to deliver automated fidelity reports for every session at every active site, replacing the 10%‐15% of sessions that now receive any human review [26,93]. Once cross-dimension reliability reaches an adequate threshold, the next study is a fully powered cluster-randomized noninferiority trial comparing AI-augmented with standard supervision on youth depression and anxiety outcomes, provider skill, and cost-effectiveness. Beyond supervision, the session-level data shamiriAI generates can support causal mediation analyses, precision matching of students to providers, and identification of active ingredients [94]—a learning-system capacity that distinguishes it from a stand-alone monitoring tool and positions it as the backbone of an AI-native stepped-care model.

The work’s importance rests on the supervision bottleneck. Kenya has about 2 specialized mental health workers per 100,000 people; sub-Saharan Africa has 1.4‐3.8 [8,9]. Task-shifting is the primary response to this gap and its effectiveness is well established [18,30,91], but supervision is the binding constraint on quality and scale [18,95,96]. AI fidelity monitoring does not replace supervisors; it gives them the reach to supervise hundreds of providers across thousands of sessions. This pilot is the first evidence that such a system is technically buildable where it is most needed.

### Recommendations

The pilot specifies a concrete version 2 agenda: prompt redesign for Required Contents and Clarity with session-specific checklists and behavioral anchors, sequential per-element evaluation, Thoroughness and Skill anchors separating technique frequency from depth and timing, cross-rate feature normalization, and within-corpus evaluation of the zero-shot ASR baseline, diarization error, PII recall, and the prosodic ablation, all on the larger cohort. In parallel, future development should adopt incremental prompt version control with a dated changelog, prespecify arm-balanced sampling, audit PII redaction recall, and add calibration training for human raters. Beyond version 2, multihub replication and extension to non-Shamiri interventions and other low- and middle-income country contexts should inform version 3.

### Conclusions

Within a 52-session pilot, this study establishes 2 foundational results for shamiriAI. The fine-tuned multilingual Whisper pipeline achieved CER of 0.19 and cosine semantic similarity of 0.77 on naturalistic, multispeaker, code-switched English-Kiswahili-Sheng sessions, sufficient to support downstream LLM-based fidelity inference. The fidelity-rating system produced a dimension-dependent reliability profile: low ICCs against the continuous human composite (−0.06 to 0.20), attributable to restriction of range, with AC2 sensitivity analyses corroborating substantial agreement on Specifics, Skill, and Purity (0.69‐0.76, approaching the human-human ceiling of 0.42‐0.60) and systematic underrating on Required Contents and Clarity. The results characterize current technical performance only; whether AI-augmented supervision improves provider skill, fidelity at scale, or student outcomes will be tested in the planned cluster-randomized noninferiority trial.

## Supplementary material

10.2196/95063 Multimedia Appendix 1 Supplementary materials.

10.2196/95063 Checklist 1 TRIPOD+LLM checklist.

10.2196/95063 Checklist 2 CONSORT-eHEALTH checklist (V 1.6.1).

## Abbreviations

ASR: automatic speech recognition
BERT: Bidirectional Encoder Representations from Transformers
CBT: cognitive behavioral therapy
CER: character error rate
DER: diarization error rate
DUISERC: Daystar University Institutional Scientific and Ethical Review Committee
HIC: high-income country
ICC: intraclass correlation coefficient
KUERC: Kenyatta University Ethics Review Committee
LaBSE: Language-agnostic BERT Sentence Embedding
LLM: large language model
MFCC: mel-frequency cepstral coefficient
NACOSTI: National Commission for Science and Technology
NER: named entity recognition
PACTR: Pan African Clinical Trials Registry
PII: personally identifiable information
VAD: voice activity detection
WER: word error rate
