# Supplementary material for: Automated Fidelity Monitoring of Lay-Delivered Mental Health Interventions Using Large Language Models: Development and Pilot Validation of shamiriAI in Kenya
Source: JMIR AI. 2026 Jul 23;5:e95063. doi: 10.2196/95063 (PMC13396917; doi:10.2196/95063)
Supplement: Multimedia Appendix 1 [file ai-v5-e95063-s001.pdf]

shamiri

Multimedia Appendix 1: Supplementary Materials

shamiriAI: Automated Fidelity Monitoring of Lay-Delivered Mental Health Interventions Using Large Language Models

Authors: Shadrack Lilan[1*], Brandon Mochama[1], Tom L. Osborn[1], Wendy Mmbone[1], Rachael Kilonzo[1], Faith Kamau[1], Rahim Daya[1], and Christine Wasanga[1,2]

[1]Shamiri Institute, Nairobi, Kenya

[2]Department of Psychology, Kenyatta University

***Corresponding Author:** Shadrack Lilan (shadrack.lilan@shamiri.institute; 13th Floor, CMS Africa, Chania Avenue, Nairobi, Kenya)

Note. Supplementary materials including datasets and analysis code are publicly available on the Open Science Framework: https://doi.org/10.17605/OSF.IO/NU9TZ

Table of Multimedia Appendices

- **Multimedia Appendix 1. Supplement A** — The Shamiri intervention: theoretical background, the four-week group-format structure, and session-by-session content of the school-based adolescent mental health program evaluated in this pilot (six secondary schools, Ngong Hub, Kajiado County, Kenya, May–September 2025).
- **Multimedia Appendix 2. Supplement B** — Lay-provider recruitment, pre-intervention training, and ongoing supervision, together with the training and calibration procedures for the human fidelity raters whose ratings served as the reference standard.
- **Multimedia Appendix 3. Supplement C** — The shamiriAI processing pipeline: the fidelity rating instrument, audio ingestion and preprocessing, multilingual automatic speech recognition, speaker diarization, prosodic and conversational feature extraction, PII scrubbing, large language model inference hyperparameters and output schema, the production system prompt, and the test–retest stability analysis.
- **Multimedia Appendix 4. Supplement D** — Descriptive results: participant flow, baseline lay-provider characteristics, and the distribution of the 52 validation sessions by session type and supervision arm.
- **Multimedia Appendix 5. Supplement E** — Interrater-reliability detail: the Gwet's AC2 ordinal weighting convention, human–human agreement across the six fidelity dimensions, discrete-vs-continuous sensitivity analyses, demographic subgroup and per-arm robustness checks, and ceiling-effect characterization of the human reference distribution.

Supplement A. Overview of the Shamiri Intervention

A1. Background and Theoretical Framework

The Shamiri intervention [1–3] is a four-session, group-based psychological wellness program for Kenyan secondary school adolescents, grounded in the "wise intervention" framework [4–6]. It targets three core psychological mechanisms shown in randomized trials to improve mental health: growth mindset [7,8], gratitude [9,10], and values affirmation [11,12]. Sessions are delivered by lay providers ("Shamiri Fellows") in groups of 7–15 students during school hours (60 minutes per session), with weekly between-session homework [2,13].

A2. Intervention Structure

Property	Value
Sessions	4 weekly meetings × 60 minutes
Homework	Weekly between-session assignments
Group size	7–15 students
Language	English and Kiswahili (code-switched per group needs)
Delivery	Lay providers with 10-hour pre-intervention training and weekly supervision

A3. Session-by-Session Content

- **Session 1 — Growth Mindset I: Understanding Growth.** Students are introduced to the concept of neuroplasticity and personal growth [7,8]. Components: didactic introduction to neuroplasticity (the brain's capacity to change with effort); article and discussion on growth mindset concepts; testimonials from well-known figures and the lay provider; written activity in which students write their own personal growth story; homework noticing a challenge and writing about how growth could apply [2].
- **Session 2 — Growth Mindset II: Strategies for Growth.** Building on Session 1 with practical strategies for overcoming challenges. Components: review of Session 1 homework; brainstorming effective strategies for applying growth mindset; introduction of a structured problem-solving framework; written letter to a friend explaining what they learned plus written application of the framework to a current challenge; homework applying the framework to a specific real challenge [2].
- **Session 3 — Gratitude.** Introduces gratitude as a documented mechanism of well-being [9,10]. Components: discussion of the importance of gratitude and supporting research; written gratitude letter to someone the student wishes to thank; introduction of the "Three Good Things" daily activity; homework practicing Three Good Things daily for one week [2].
- **Session 4 — Values (Virtues) Affirmation.** Final session focuses on identifying and enacting personal values [11,12]. Components: stories of culturally pertinent role models and discussion of how their values contributed to meaningful lives; lay provider shares their own personal values and story; written activity in which students select values from a list, write about a time they demonstrated a value, and plan a specific future action; homework taking one concrete action aligned with a personal value [2].

A4. Theoretical Foundations

Growth mindset. Targets the implicit belief that personality and intelligence are fixed, promoting malleability and resilience [7,7,14].

Gratitude. Teaches individuals to notice and appreciate positive experiences, improving well-being and social relationships [9,10].

Values affirmation. Encourages reflection on self-defining values, reaffirming personal worth and mobilizing internal resources [11,12].

Components were selected and culturally adapted for Kenyan secondary school adolescents through iterative review, expert consultation, and feedback from recent Kenyan graduates [2,3].

Supplement B. Lay-Provider and Clinical Supervisor Training and Supervision

B1. Lay-Provider Recruitment and Selection

Lay providers ("Shamiri Fellows") were recruited openly through WhatsApp groups, university forums, and online job-posting boards. Eligibility criteria: at least 18 years old; completed secondary school in Kenya with English as the language of instruction; able to read intervention protocols in English; available for all scheduled group sessions. Interested candidates completed online applications and participated in structured 30-minute interviews assessing interest, relevant experience, personal characteristics conducive to group leadership, and responses to hypothetical implementation scenarios [2,13].

From the applicant pool, 64 candidates were assigned to the Ngong Hub and were thus eligible for this study; 47 participated in the parallel A/B test (34 in the AI-augmented arm; 13 in the standard supervision arm).

B2. Pre-Intervention Training

- **Total duration.** 10 hours across 2 training days.
- **Trainers.** Undergraduate study team members supervised by clinical staff, consistent with the established Shamiri evidence-based training model (Venturo-Conerly et al., 2021; Osborn et al., 2020).
- **Structure.** Emphasis on role play over lecture; all Fellows trained in both intervention and control conditions.

Component	Description
Didactics (~1 hour)	Logistical details and compensation; privacy and confidentiality of student participants; scientific nature of the study; strict protocol adherence and not mixing condition content; plans for weekly supervision; data security procedures; emergency procedures for suicidality or serious concerns
Role plays (core of training)	Each Fellow completed approximately one 30-minute role play of intervention content as the lay provider and participated as a "group member" in role plays of all session content. More time was allocated to wellness-intervention role plays. Fellows received feedback from trainers and peers after each role play.
Core skills (reinforced throughout)	Stick to the protocol; ask questions and let participants speak; respect confidentiality; reach out to your supervisor in any emergency.
Session materials	Detailed session outlines for all conditions, in English; discussions could be conducted in English or Kiswahili.

B3. Ongoing Supervision Protocol

Weekly supervision meetings. Frequency: twice weekly, 30 minutes per meeting. Content: review of session content; structured time for Fellows to raise concerns and questions; discussion of group dynamics, protocol challenges, or student welfare concerns; preparation for the upcoming session. Additional communication: study team used WhatsApp for important reminders and next-day preparation notes [2,13].

Daily on-site supervision. Each Fellow was assigned a daily supervising team member at their school on each session day for the following functions: distributing session materials before sessions and collecting them after; providing at least one time warning per session; remaining available in the general area (not near groups) for Fellows to approach; and discreetly intervening in clear protocol deviations (e.g., providing a time reminder to a significantly behind group) [2,13,15].

B4. Rater Training for Fidelity Coding

Independent human fidelity raters underwent a standardized training procedure: didactic training on the Shamiri intervention protocol and the six-domain fidelity rubric; mock coding of two randomly selected session recordings; calibration and feedback session with a study team member; and inter-rater reliability check before independent coding proceeded [1–3].

Per the Methods (Fidelity Rating subsection), all raters who scored the 52 validation sessions were drawn exclusively from Shamiri hubs other than Ngong Hub; none had been assigned to the AI-augmented supervision arm or had received or reviewed any shamiriAI-generated feedback reports prior to or during the rating process.

B5. AI-Augmented Supervision Condition (shamiriAI Arm)

In the AI-augmented condition, supervisors received structured shamiriAI-generated session feedback reports (see Supplement C12) prior to weekly supervision meetings [15]. Reports were delivered as PDF files via secure email or shared folder. Supervisors retained full discretion over how AI outputs were interpreted, weighted, and communicated to Fellows during supervision. The intent of AI augmentation was to extend supervisors' observational reach across sessions that would otherwise go unreviewed, not to replace human clinical judgement.

B6. Standard Supervision Condition

In the standard supervision condition, structure, frequency, and format of human supervision meetings were identical to the AI-augmented condition. Supervisors did not receive AI-generated feedback reports; supervision drew on direct observation, Fellow self-report, and the supervisor's own clinical judgement.

Supplement C. Fidelity Rating Instrument and shamiriAI Pipeline

C1. Fidelity Rating Instrument

Session fidelity is assessed using a six-domain rubric adapted from the instrument validated across prior Shamiri randomized trials. Ratings are made on a 7-point Likert scale (1 = worst, 7 = best) [1–3]. Raters were trained on both the intervention protocol and the rubric before independent coding.

Domain	Construct	Description
Required Contents	Protocol Adherence — Content	Extent to which the lay provider delivered all required session content. High = all core content covered; low = significant omissions.
Specifics	Protocol Adherence — Procedural	Adherence to specified procedural requirements (e.g., distributing worksheets, completing written activities, following the specified session structure).
Thoroughness	Depth of Coverage	Degree to which content was delivered with sufficient depth and completeness rather than superficially. Reflects quality of engagement with each content element.
Clarity	Communication Quality	How clearly and accessibly the lay provider communicated session content. High = easy to understand; low = confused, unclear, or inaccessible.
Skill	Facilitation Competence	Overall skillfulness in facilitating the group: engaging participants, managing group dynamics, using counselling techniques (validation, open questions), adapting responsively to the group.
Purity	Protocol Integrity	Absence of off-protocol content. High = only required content delivered; low = introduction of extraneous or cross-condition material.

Note. All six domains rated 1–7. Average fidelity is the mean across all six domains. Interrater reliability assessed using Gwet's AC2 with ordinal weights[16,17]; see Supplement E1.

C2. shamiriAI Pipeline Architecture

shamiriAI is a multimodal AI pipeline that processes raw session audio to generate structured fidelity feedback for lay providers and supervisors, designed for multilingual code-switched speech (English, Kiswahili, Sheng).

Stage	Component	Description
1	Audio ingestion	Raw audio files (.mp3 / .wav) uploaded to secure cloud storage by hub coordinators. A scheduled server-based pipeline retrieves new files for processing.
2	Preprocessing	Audio denoising and voice activity detection (VAD) to mitigate background noise from open school environments.

3a	Multilingual ASR	Transcription using a fine-tuned Whisper-small model (~242M parameters), with speaker diarization (Supplements C3–C5).
3b	Prosodic feature extraction	Paralinguistic features extracted using Librosa across six categories (Supplement C6).
4	PII scrubbing	Personally identifiable information removed from transcripts before LLM inference (Supplement C7).
5	LLM inference	Cleaned transcripts and prosodic feature summaries passed to Gemini 2.5 Pro via a structured system prompt (Supplements C8–C11).
6	Report delivery	Feedback reports converted to PDF and distributed to supervisors for use in weekly supervision meetings (Supplement C12).

C3. Audio Ingestion and Preprocessing

Hub Coordinators uploaded session audio files to secure cloud storage following each session. A scheduled server-based pipeline retrieved new files for processing. Preprocessing included three steps: (a) format normalization, converting all files to mono for downstream model input; (b) denoising and audio enhancement via spectral gating and noise reduction to mitigate background noise from open school environments; and (c) voice activity detection to segment audio into speech and non-speech regions, capturing conversational structure.

Sampling rate distribution. Sampling rates were not stored during pipeline processing and were recovered post-hoc from the raw WAV files using `ffmpeg` (`ffprobe -v error -select_streams a:0 -show_entries stream=sample_rate`). Across the validation corpus, three sampling rates were observed:

Sampling rate	Sessions	Percent
22050 Hz	22	42.3%
44100 Hz	12	23.1%
48000 Hz	18	34.6%
Total	52	100%

All audio was loaded for prosodic feature extraction using Librosa with `sr=None`, preserving the native sampling rate throughout the signal-processing pipeline. Consequently, spectral features (centroid, bandwidth, contrast), MFCCs, and pitch-tracking outputs are computed at the native rate per file and are not directly comparable across sessions recorded at different rates without normalisation. Feature normalisation across rates is identified as a v2 priority (Limitations, §6). The diarization pipeline (Supplement C5) ingests at 16 kHz internally with automatic downmixing/resampling, so the diarization stage is not affected by source-rate variation.

C4. Multilingual Automatic Speech Recognition (ASR)

Model selection and architecture. We used the open-source Whisper-small checkpoint (~242M parameters) as the backbone of the transcription pipeline. Whisper was selected for three reasons: its strong reported performance on multiple languages including Kiswahili; its open-source licensing;

and its capacity for on-premise deployment, which allowed us to avoid transmitting sensitive school-based recordings to third-party servers.

Fine-tuning. The base model was fine-tuned on an 8-hour training split of the Shamiri corpus (2023–2024 historical recordings) to improve performance on code-switched Kenyan adolescent speech. Fine-tuning hyperparameters: learning rate 1×10^{-5}; batch size 16 per device; 5,000 training steps; standard data augmentation (time masking and additive noise) for robustness to recording-quality variation. The Shamiri corpus was split into training (8 hours), validation (1 hour), and held-out test (1 hour) sets; performance metrics reported in the main manuscript Results section are based exclusively on the held-out test set.

Zero-shot baseline (literature-based). To contextualize the performance of the fine-tuned model, we report zero-shot baseline figures from the published literature rather than running a separate within-corpus zero-shot evaluation. Zero-shot Whisper-medium performance on Kiswahili is not directly reported in the original Whisper release paper [18] but scales predictably with per-language pre-training volume. Published fine-tuning work on Swahili using Mozilla Common Voice reports zero-shot Whisper-medium WER in the range of 0.51–0.60 on read speech before domain adaptation [19]. For code-switched speech — which introduces intra-sentence language alternation not present in standard benchmarks — zero-shot WER is expected to be substantially higher, as Whisper was not trained explicitly on code-switched data and exhibits poor zero-shot generalization to novel code-switching patterns [20]. Our fine-tuned model achieved WER 0.34 on the held-out test set, an improvement consistent with the benefit of domain adaptation on 8 hours of in-domain code-switched audio. The reliance on literature benchmarks rather than a within-corpus zero-shot baseline is acknowledged as a limitation and committed for empirical evaluation in a follow-up validation study.

Transcription performance metrics. Because sessions involve code-switching and agglutinative morphology (particularly in Kiswahili), we evaluated ASR performance using a combination of metrics including Word Error Rate (WER) and Character Error Rate (CER), as well as semantic similarity via cosine similarity between sentence embeddings from a multilingual model (LaBSE). On the held-out test set: WER 0.34; CER 0.19; cosine semantic similarity 0.77; ROUGE-L 0.60. CER and cosine semantic similarity are treated as primary indicators of model quality given the linguistic context, with WER reported for comparability with other published ASR systems.

C5. Speaker Diarization

Speaker diarization was performed using `pyannote/speaker-diarization-3.1` [21,22], a fully automatic neural pipeline requiring no manual voice activity detection, no manual speaker count, and no dataset-specific fine-tuning. The pipeline ingests mono audio at 16 kHz, automatically downmixing and resampling as needed. No domain adaptation or hyperparameter tuning was applied for the Shamiri corpus.

Diarization Error Rate (benchmark figures). In the absence of manually annotated reference diarizations for the 52 validation sessions, session-level DER could not be computed on the Shamiri corpus directly. We report published benchmark DER from the official model card as an indication of expected performance under controlled conditions. Benchmark DER is reported under the least forgiving evaluation setup: no forgiveness collar; overlapping speech included.

Table C5.1. Published benchmark DER for *pyannote/speaker-diarization-3.1*

Benchmark	DER%	False Alarm%	Missed Speech%	Speaker Confusion%
AISHELL-4	12.2	3.8	4.4	4.0

AliMeeting (channel 1)	24.4	4.4	10.0	10.0
AMI (headset mix, only_words)	18.8	3.6	9.5	5.7
AMI (array1, channel 1, only_words)	22.4	3.8	11.2	7.5
AVA-AVD	50.0	10.8	15.7	23.4
DIHARD 3 (Full)	21.7	6.2	8.1	7.3
MSDWild	25.3	5.8	8.0	11.5
REPERE (phase 2)	7.8	1.8	2.6	3.5
VoxConverse (v0.3)	11.3	4.1	3.4	3.8

Note. All figures from the official model card (huggingface.co/pyannote/speaker-diarization-3.1). DER = diarization error rate. Multi-speaker meeting comparators (AMI, DIHARD 3) suggest a plausible expected DER range of 18–22% for our setting, though the code-switched multilingual context and overlapping adolescent speech could push error rates higher. The actual DER on the Shamiri corpus is unknown; corpus-specific DER estimation is committed for a follow-up validation study.

C6. Prosodic and Conversational Feature Extraction

In parallel with ASR, we extracted a set of prosodic and conversational features using deterministic signal-processing techniques implemented in *librosa* and related audio libraries. These features were designed to capture dimensions of group dynamics and facilitator behaviour that are not reflected in transcribed text alone, and were included as supplementary inputs to the LLM feedback inference stage (Supplement C11).

Feature category	Features and rationale
Spectral features	Spectral centroid (frequency "centre of mass," associated with vocal brightness and energy); spectral bandwidth (spread of the frequency distribution, related to clarity of articulation); spectral contrast (differences between spectral peaks and valleys, linked to expressiveness and vocal variation).
Speech segment dynamics	Number of speech segments (conversational turns); mean and SD of segment durations; mean and SD of inter-speaker gaps; quick exchange ratio (proportion of gaps below a short-gap threshold). Proxies for interaction quality, equitable participation, pacing, and conversational flow.
Voice diversity metrics	Pitch diversity (variation in fundamental frequency across speakers and time); energy diversity (variation in vocal intensity). Higher diversity may indicate more distributed participation rather than facilitator-dominated delivery.
Conversation flow indicators	Speech density (proportion of speech vs. silence within sliding temporal windows); mel-frequency cepstral coefficients (MFCCs, summarising timbral characteristics); rhythm features (tempo and periodicity).

Temporal dynamics	Trajectories of pitch and energy across the session; shifts in turn-taking patterns over time. Captures the therapeutic arc of a session and notable moments of change in group energy.
Voice quality indicators	Zero crossing rate; harmonic-to-noise ratio; approximations of jitter and shimmer. Reflects vocal tension or emotional states that may signal group discomfort or facilitator stress.

All features were computed at both the segment level and the session level. The format in which features were passed to the LLM is described in Supplement C11.

C7. PII Scrubbing — NER-Based Redaction

Before passing transcripts to the LLM, we applied a privacy-protection step. PII scrubbing was performed using **NuNerZero** (`numind/NuNerZero`), a zero-shot named-entity recognition model based on the GLiNER architecture [23]. NuNerZero is a compact bidirectional token classifier (not a generative LLM), trained on the NuNER v2.0 dataset (a combination of Pile and C4 subsets annotated via LLMs). It was selected for its zero-shot capability — requiring no labelled training data for the target domain — and its support for arbitrary entity-label prompting at inference time without fine-tuning.

Entity types and placeholders. Three entity types were targeted: person, location, and organization. Detected entities were replaced in-place with typed placeholders: [REDACTED_PERSON], [REDACTED_LOCATION], [REDACTED_ORGANIZATION]. Redaction was applied to ASR output transcripts before any content was passed to the LLM inference stage; audio files and unredacted transcripts were retained on secure servers with restricted access. An English BERT-based NER model (dslim/bert-base-NER) was implemented as a fallback but was not used in production.

Limitations and mitigations. Formal precision and recall metrics for the redaction pipeline were not computed in this pilot. Systematic evaluation of NER performance on code-switched Kiswahili–English text is an active research challenge: standard NER benchmarks are predominantly monolingual English, and no published benchmark exists for Kenyan adolescent code-switched speech. In qualitative review, person-name redaction performed acceptably, given the alignment between Kenyan firstname-middlename-familyname conventions and the model's English-corpus pretraining. The primary under-redaction risk was concentrated in location and organization entities — particularly school names expressed in Kiswahili or local languages, location references embedded mid-utterance in code-switched speech, and informal references using Sheng terms. For the present pilot, the combination of secure server storage for unredacted files, restricted access controls, and participant consent procedures (Methods, Ethical Considerations) constitutes the primary privacy protection mechanism; NER-based redaction is treated as a supplementary layer rather than the sole safeguard. A stratified manual audit of redacted transcripts by bilingual annotators familiar with Kenyan names and locations is committed as a primary deliverable for a follow-up validation study.

C8. LLM Inference — Hyperparameters and Output Schema

The fidelity scoring component of the shamiriAI pipeline used Google Gemini 2.5 Pro (gemini-2.5-pro) to evaluate each session against the Shamiri Intervention Protocol rubric. The model was invoked once per session, receiving the diarized transcript and session-level audio features as structured JSON input. Generation hyperparameters were fixed identically across all 52 validation sessions.

C8.1. LLM generation hyperparameters used in production scoring

**Parameter**	**Value**	**Notes**
Model	`gemini-2.5-pro`	Google DeepMind, 2025
Temperature	1.0	Stochastic generation
Top-p	0.95	Nucleus sampling threshold
Top-k	40	Vocabulary restriction per decoding step
Max output tokens	65,536	Upper bound on response length
Thinking budget	−1 (dynamic)	Model-controlled extended reasoning; no fixed token cap
Output MIME type	`application/json`	Enforced structured output
Response schema	Pydantic `FidelityAnalysisResponse`	Schema enforces integer 1–7 scores; see C8.2 below
Seed	None set	Generation is stochastic; score structure enforced by schema

C8.2 Structured output schema.

The model returned a JSON object with four top-level sections: fidelity_scores (one integer score and free-text justification per rubric dimension, plus overall score and assessment), qualitative_feedback (session summary, strengths, areas for improvement, session-flow assessment), safety_flags (structured objects with type, severity, temporality, and confidence), and recommendations (actionable improvement suggestions). The complete schema is reproduced below:

```
{
  "fidelity_scores": {
    "question_1_protocol_adherence":     { "score": 1-7, "justification": "..."
},
    "question_2_content_specifications": { "score": 1-7, "justification": "..."
},
    "question_3_thoroughness":           { "score": 1-7, "justification": "..."
},
    "question_4_skillful_delivery":      { "score": 1-7, "justification": "..."
},
    "question_5_clarity_accessibility":  { "score": 1-7, "justification": "..."
},
    "question_6_protocol_boundaries":    { "score": 1-7, "justification": "..."
},
    "overall_score": "mean of above scores",
    "overall_assessment": "..."
  },
  "qualitative_feedback": {
```

```
    "session_summary": "...",
    "strengths": ["..."],
    "areas_for_improvement": ["..."],
    "session_flow_and_engagement": "..."
  },
  "safety_flags": [
    {
      "type": "suicidality | self_harm | substance_abuse | other",
      "description": "...",
      "timestamp_reference": "relative description",
      "severity": "low | medium | high",
      "immediate_action_needed": true | false,
      "context_analysis": "...",
      "current_vs_past": "current | past | educational | unclear",
      "confidence_level": "high | medium | low",
      "requires_follow_up": true | false
    }
  ],
  "recommendations": ["..."]
}
```

No post-processing or regex extraction was applied to model outputs; scores were consumed directly from the validated JSON object. Because no random seed was fixed, generation is stochastic; score-level variance across repeated calls is bounded by the discrete 1–7 output schema, and was empirically characterised through the test-retest reliability analysis reported in Supplement C8.3.

C8.3. Test-Retest Stability Analysis

To empirically characterise score-level variance arising from stochastic generation at the production temperature (1.0, with no fixed seed), we conducted a test-retest reliability analysis on all 52 validation sessions. The locked production prompt (iteration_three.md; Supplement C9) was run three times per session at the production hyperparameter settings (Supplement C8.1). For each rubric dimension and for the overall composite, we computed agreement across the three independent runs using Gwet's AC2 with ordinal weights—the same coefficient, weighting, and irrCAC implementation used for the human inter-rater and AI–human agreement analyses elsewhere in this study—so that all agreement coefficients are reported on a common footing. For continuity with the prior submission we also report the intraclass correlation coefficient ICC(3,1) (two-way mixed-effects, single-measures, consistency). The overall composite was rounded to the nearest integer before the AC2 computation, parallel to the rounded-composite convention used in the AI–human analysis.

Table C8.3.1. Test-retest agreement (Gwet's AC2, ordinal weights) and ICC(3,1) for shamiriAI fidelity ratings across three independent runs at production settings (temperature = 1.0)

Dimension	Gwet's AC2 [95% CI]	ICC(3,1) [95% CI]	Interpretation (AC2)
Q1: Protocol Adherence (Required Contents)	0.871 [0.836, 0.906]	0.607 [0.460, 0.734]	Almost Perfect
Q2: Content Specifications (Specifics)	0.954 [0.936, 0.972]	0.730 [0.613, 0.824]	Almost Perfect

Q3: Thoroughness	0.878 [0.848, 0.908]	0.600 [0.452, 0.729]	Almost Perfect
Q4: Skillful Delivery (Skill)	0.866 [0.808, 0.923]	0.502 [0.340, 0.653]	Almost Perfect
Q5: Clarity & Accessibility	0.936 [0.903, 0.968]	0.735 [0.619, 0.827]	Almost Perfect
Q6: Protocol Boundaries (Purity)	0.865 [0.784, 0.946]	0.437 [0.269, 0.599]	Almost Perfect
**Overall composite**	**0.930 [0.905, 0.954]**	**0.708 [0.584, 0.808]**	**Almost Perfect**

Note. N = 52 sessions, 3 runs per session. AC2 = Gwet's AC2 computed with ordinal weights via the irrCAC package, matching the coefficient used for the human inter-rater and AI–human analyses. AC2 interpreted by Landis & Koch (1977) benchmarks (0.61–0.80 = Substantial, 0.81–1.00 = Almost Perfect); ICC(3,1) interpreted by Koo & Li (2016) benchmarks (< 0.50 = Poor, 0.50–0.75 = Moderate, 0.75–0.90 = Good, > 0.90 = Excellent). All ICC F-tests were significant at p < .001.

Interpretation. Test-retest agreement at the production temperature is high and uniform across the rubric: Gwet's AC2 ranges from 0.87 (Protocol Boundaries) to 0.95 (Content Specifications), with an overall composite of 0.93—all in the Almost Perfect range—and the three runs agree on 92–98% of cases. The corresponding ICC(3,1) values are substantially lower (0.44–0.74), reaching only the Moderate range. This divergence reflects the same restriction-of-range and ceiling effects that suppress variance-based coefficients throughout this study (Supplement E4): ICC penalises the concentrated score distribution, whereas AC2 does not. The two coefficients nonetheless rank the dimensions concordantly, with the lowest stability on Protocol Boundaries (Purity) and Skillful Delivery (Skill) and the highest on Content Specifications (Specifics) and Clarity.

Because this is an intra-rater (test-retest) coefficient, it is expected to exceed the inter-rater agreement observed among the human raters (Gwet's AC2 0.42–0.60; Supplement E1) and should not be interpreted as evidence that the model matches a human-agreement benchmark—the two are not parallel comparisons. We therefore read the high test-retest AC2 as indicating that stochastic generation at temperature 1.0 introduces only modest run-to-run variability, not as a claim of equivalence to human reliability. This residual variability is an inherent property of using a generative model for a discriminative scoring task and is noted as a limitation in the main text; deployments requiring fully deterministic scores would use a temperature of 0 or aggregate scores across repeated runs.

The full per-session per-run score matrix, the AC2 and ICC computation scripts, and a session-level summary CSV are archived on the Open Science Framework (DOI: 10.17605/OSF.IO/NU9TZ) for full reproducibility.

C9. Production System Prompt (iteration_three.md)

The system prompt used for all 52 validation sessions is reproduced verbatim below.

```
You are an expert psychologist with extensive experience in:

  1. Providing psychological interventions to high school youth in low-middle-i
     ncome countries

  2. Facilitating and supervising group therapy sessions with adolescents
```

3. Training and evaluating lay providers who lead peer counseling interventions

4. Assessing intervention fidelity using structured protocols

Your Task. You will evaluate the fidelity of a group therapy session based on the Shamiri Intervention Protocol. You will receive two JSON files.

JSON Input Structure.

Transcript JSON (Array Format). Each array element represents a speaker utterance with: speaker_id (numeric identifier), text (spoken words, may include [inaudible]), start/stop (timestamp boundaries in seconds), is_session_lead (boolean identifying the facilitator), duration (seconds), pitch_mean/pitch_range (voice pitch characteristics), energy_mean/energy_std (voice energy/volume levels), speaking_rate (words per second).

Audio Features JSON (Object Format). Session-level acoustic analysis including: spectral_centroid_mean/std (voice clarity and brightness), tempo (session pacing), mfcc_* (voice timbre characteristics), speech_segments_count (total speaking turns), speech_duration_mean/std (average contribution length), speech_gap_mean/std (average silence between speakers), quick_exchange_ratio (proportion of rapid exchanges), pitch_diversity (emotional expressiveness), energy_diversity (speaking intensity variation), speech_density_mean/std (proportion of session with active speech).

Evaluation Framework — Core Protocol Elements to Assess.

1. **Explaining Confidentiality (MANDATORY).** Must emphasise confidentiality and explain the one scenario for breaking it (life at risk), done jovially with an example.

2. **Validating (CRITICAL).** Must make participants feel their feelings are valid using empathetic language such as “It sounds like you’re feeling [emotion]. That makes sense given what you’re going through.”

3. **Open-Ended Questions (REQUIRED).** Must use elaboration prompts and prefer open-ended over closed-ended questions.

4. **Rephrasing (MANDATORY).** Must rephrase student contributions using the student’s own words and summarise for the group.

5. **Verbal Nodding.** Must use verbal acknowledgments (“mhm,” “yeah”) to show active listening.

6. **Juxtapositions.** When students express conflicting perspectives, must highlight and explore the contrast.

7. **Keeping Topics on Track.** Must cover all required material and use specific transitional phrases when redirecting.

8. **Connecting Between Students (REQUIRED).** Must explicitly connect themes between different students to build community.

9. **Reflecting (MANDATORY 3-STEP).** At end of each segment: (1) summarise the lesson, (2) reference specific student contributions, (3) transition to next topic.

10. **Silence.** Must use silence strategically as a therapeutic tool.

11. **Relationship Management.** Must maintain appropriate boundaries while creating a warm, safe environment.

Grading Rubric.

Rate each dimension on a 1-7 scale (1–3 low adherence; 4 neutral; 5–7 high adherence).

- Question 1 — Protocol Adherence. Completion of all main protocol sections; all 11 elements present in some form.
- Question 2 — Content Specifications. Coverage of all important discussion points and required materials.
- Question 3 — Thoroughness. Thorough explanations; adequate time per section; participant opportunities to contribute.
- Question 4 — Skillful Delivery. Smooth transitions, appropriate humour, engaging style, therapeutic skill beyond content delivery.
- Question 5 — Clarity/Accessibility. Clear, age-appropriate, culturally sensitive language.
- Question 6 — Protocol Boundaries. No self-created content outside the protocol.

Safety Assessment Framework.

Before flagging any concern, assess: Is this a current issue or past experience? Who is speaking — facilitator appropriate self-disclosure or participant seeking help? Is the purpose educational or a personal disclosure? Only flag as high priority if there are clear indicators of current, ongoing risk.

Audio Feature Interpretation Guide.

- quick_exchange_ratio → "conversation flow and turn-taking patterns"
- pitch_diversity → "emotional expressiveness and vocal variation"
- energy_diversity → "speaking intensity and engagement levels"
- speech_density → "how much of the session involved active discussion"
- speech_gap_mean → "pacing and use of silence"
- tempo → "overall session rhythm"

Output Format. Return ONLY a valid JSON object matching the response schema. No markdown, no code blocks, no additional text. Use relative temporal references (e.g., "early in the session," "about halfway through"). Treat each session as a fresh, independent evaluation.

C10. Prompt Development Timeline

The shamiriAI fidelity scoring prompt was developed in three iterations prior to the validation study. All three versions are preserved in the project repository (prompt_folder/iteration_one.md,

iteration_two.md, iteration_three.md) and on the Open Science Framework repository (DOI: 10.17605/OSF.IO/NU9TZ).

Iteration one established the evaluator persona (expert psychologist with adolescent and LMIC experience), defined the four output components (rubric scores, qualitative feedback, timestamp references, safety flags), and set the instruction to avoid technical jargon from the JSON inputs.

Iteration two introduced a structured multi-step evaluation approach: explicit protocol review before scoring; separate analysis of the transcript and audio feature inputs; rubric application with evidence-based justification; and a final synthesis step. The output format remained narrative rather than schema-enforced.

Iteration three (production) substantially expanded the evaluation framework to embed the complete Shamiri Intervention Protocol rubric: all 11 peer-counselling and group-leadership techniques with specific behavioural indicators; dimension-level grading criteria aligned to the 1–7 scale; a contextual safety-assessment decision tree distinguishing current from past disclosures; and an audio feature interpretation guide. Output was converted to a strictly typed JSON schema enforced at the API level via Pydantic validation (Supplement C8.2).

Provenance and data leakage check. All prompt iterations were developed and evaluated against earlier Shamiri Hub recordings that were separate from and did not overlap with the 52-session validation dataset. The production prompt (iteration_three.md) was locked before the validation sessions were assembled or scored. No validation sessions were reviewed, scored, or otherwise consulted during prompt development. The 52 sessions therefore constituted a clean held-out evaluation of the locked prompt, with no leakage between the prompt-development and validation phases. We acknowledge openly that prompt-development artefacts were committed to version control together at project handoff rather than incrementally; file-level timestamps therefore do not independently corroborate the chronological ordering, and the provenance account rests on author attestation rather than automated artefact evidence (Limitations). Future development will adopt incremental version control with a dated changelog linking each prompt version to the dataset on which it was evaluated.

C11. Prosodic Feature Representation in the Prompt

Session-level audio features were passed to the model as a raw JSON object with original numeric values (e.g., "spectral_centroid_mean": 2847.3, "speech_gap_mean": 1.42). No bucketing, z-scoring, or label substitution was applied before prompt construction. The system prompt provided an interpretation guide (reproduced in Supplement C9) mapping each technical field name to a plain-language description (e.g., quick_exchange_ratio → "conversation flow and turn-taking patterns"; pitch_diversity → "emotional expressiveness and vocal variation"), instructing the model to use these descriptions rather than raw field names in its output. Per-segment prosodic features were embedded directly within each transcript turn object (pitch_mean, pitch_range, energy_mean, energy_std, speaking_rate), keeping the acoustic signal co-located with the corresponding speech text in the model's context window.

Worked example (truncated for brevity). An excerpt of the input passed to the LLM for one validation session:


```
{
  "transcript": [
    {"speaker_id": 1, "text": "Habari za asubuhi...", "start": 0.5, "stop": 4.2,
     "is_session_lead": true, "duration": 3.7, "pitch_mean": 178.4,
     "pitch_range": 92.1, "energy_mean": 0.034, "energy_std": 0.012,
     "speaking_rate": 2.8},
    ...
```

```
  ],
  "audio_features": {
    "spectral_centroid_mean": 2847.3,
    "spectral_centroid_std": 412.8,
    "speech_segments_count": 287,
    "speech_duration_mean": 4.6,
    "speech_gap_mean": 1.42,
    "quick_exchange_ratio": 0.31,
    "pitch_diversity": 0.58,
    ...
  }
}
```

This format was chosen to (i) maximise interpretability of features in the model's reasoning trace, (ii) keep prompt length within the model's context window without aggressive summarisation, and (iii) preserve numerical precision for the model's pattern-recognition over feature values.

C12. Report Generation and Supervisor Delivery

For each processed session, shamiriAI generated a PDF report containing: basic session metadata (Fellow ID, school, session number, date, duration); a quantitative summary of key prosodic indicators (e.g., talk-time distribution, speech density, quick-exchange ratio); numerical ratings for each fidelity domain; and a narrative feedback section structured around observed strengths, areas for improvement, and suggested focus areas. Reports were delivered to supervisors via secure email or shared folder and reviewed during weekly supervision meetings. Supervisors were encouraged to interpret AI-generated ratings and feedback alongside their own direct knowledge of Lay providers and clinical judgement.

Supplement D. Participants and Sampling

D1. Participant Flow

The CONSORT-style participant flow diagram for lay providers is reproduced as Figure D1 (high-resolution PNG supplied as a separate supplementary file). Key counts: 64 fellows assigned to Ngong Hub were eligible; 47 participated in the parallel A/B test (34 randomized to AI-augmented supervision; 13 to standard supervision); 52 recorded sessions across these fellows were included in the fidelity validation dataset (38 from the AI-augmented arm; 14 from the standard arm). Sample sizes by session type are reported in Supplement D3.

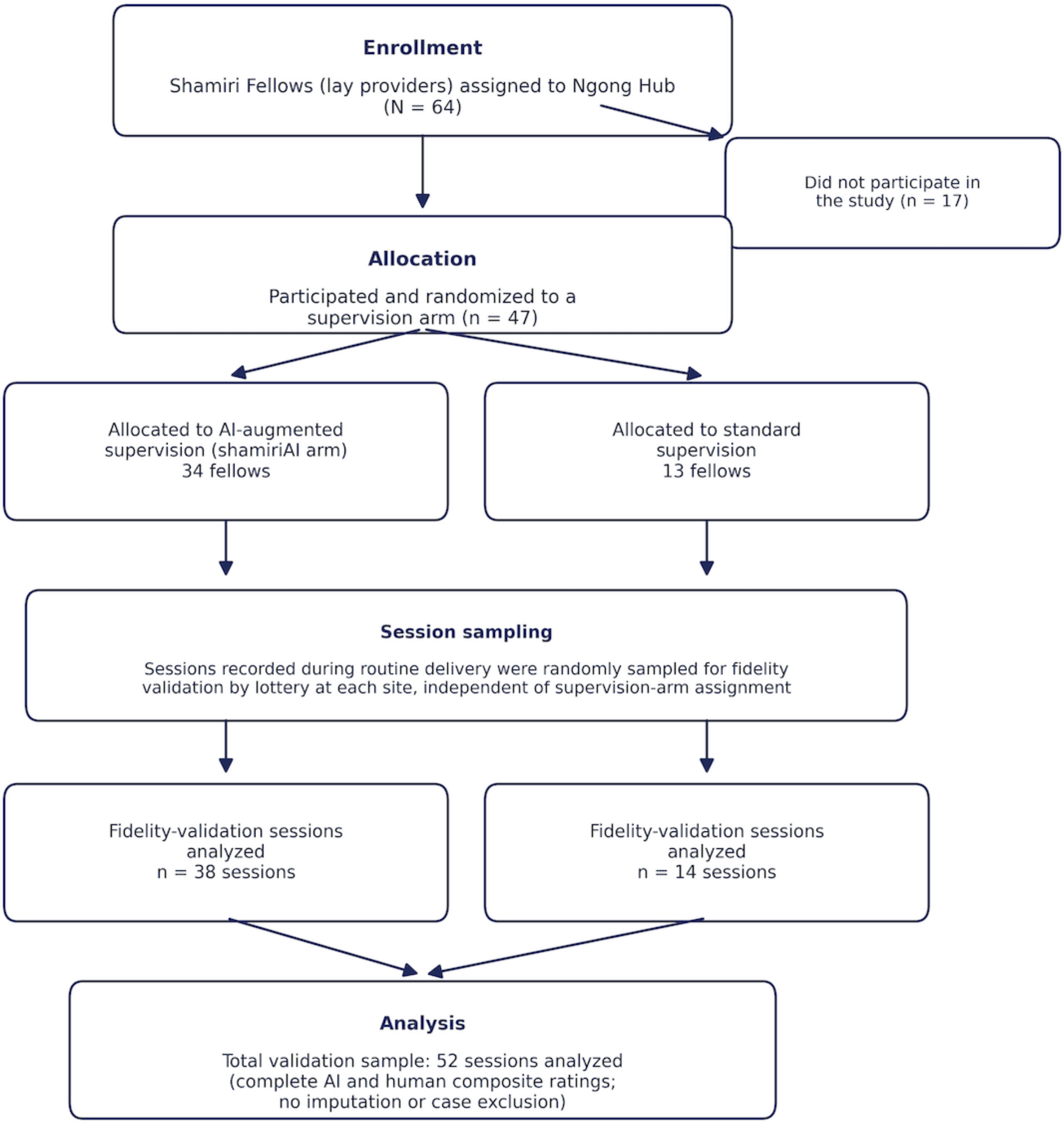

Figure D1. Consort Flow Diagram

D2. Baseline Lay-Provider Characteristics

Table D2.1. Baseline lay-provider demographics by supervision condition (N = 47 fellows; 52 sessions)

Characteristic	Overall	AI-Augmented	Standard
Fellows (n)	47	34	13
Sessions in validation set (n)	52	38	14
Age, M (SD)	19.83 (1.65)	—	—
Age range	18–23	—	—
Sex (Female), n (%)	36 (76.6%)	—	—
Sex (Male), n (%)	11 (23.4%)	—	—

Note. Demographics reported at the fellow level. Per-arm demographic breakdowns omitted from the table to preserve provider anonymity at small cell sizes. Demographic subgroup analyses by sex and age band are reported in Supplement E3.

D3. Validation Session Distribution by Session Type and Arm

Table D3.1. Distribution of the 52 validation sessions across Shamiri session types and supervision arms

Shamiri session type	AI-Augmented (n)	Standard (n)	Total (n)	% of 52
Session 1 — Growth Mindset I	14	5	19	36.5%
Session 2 — Growth Mindset II	11	4	15	28.8%
Session 3 — Gratitude	9	3	12	23.1%
Session 4 — Values Affirmation	4	2	6	11.5%
**Total**	**38**	**14**	**52**	**100%**

Note. The 52 validation sessions cover all four Shamiri session types. Session 4 (Values Affirmation) is under-represented (6 of 52 sessions, 11.5%); AC2 estimates apply most reliably to Sessions 1–3. The under-representation of Session 4 is acknowledged in the Limitations section of the main manuscript.

Supplement E. Statistical Analyses

E1. Gwet's AC2 Weighting Convention and Computation

All Gwet's AC2 coefficients reported in this paper, supplements, and figures use ordinal weights (computed via the irrCAC package in R / Python equivalent). Ordinal weighting was selected because the six fidelity dimensions are rated on an ordinal 1–7 Likert scale and adjacent disagreements (e.g., a 5 vs a 6) carry less informational weight than distant disagreements (e.g., a 2

vs a 7). Ordinal weights penalise distant disagreements more than adjacent ones and are appropriate for this data structure.

Computation note. AC2 is computed with categories inferred from the observed values rather than the full 1–7 scale (R irrCAC package default). This convention is preserved across all analyses — including those reported in Supplements E2–E5 — so that all AC2 values in the manuscript are mutually comparable. The OSF repository (DOI: 10.17605/OSF.IO/NU9TZ) contains the R script and Python equivalent reflecting this convention.

Table E1.1. Human–human inter-rater agreement (Gwet's AC2, ordinal weights) across the six fidelity dimensions, computed across all five rater pairs over the 52 validation sessions

Dimension	Human–human AC2
Required Contents (Q1)	0.59
Specifics (Q2)	0.50
Thoroughness (Q3)	0.59
Clarity (Q5)	0.60
Skill (Q4)	0.56
Purity (Q6)	0.42

Note. N = 52 sessions. AC2 = Gwet's AC2 with ordinal weights (see E1, above), computed over all five human rater pairs. Values range from 0.42 (Purity) to 0.60 (Clarity), all within the Moderate range [24], establishing the ceiling on the AI–human agreement achievable against this reference standard. Dimension labels follow the rubric numbering (Q1–Q6); see Supplement C1.

E2. Sensitivity Analyses: Discrete-vs-Continuous Formulations of the Human Reference

The primary AI–human agreement analysis is reported in the main manuscript Results (Interrater Reliability subsection) and Table 3, using ICC against the continuous human composite (Formulation B). Two sensitivity analyses are reported below to address the asymmetry between the AI's discrete integer outputs (1–7) and the continuous reference: Formulation A, Gwet's AC2 (ordinal weights) of AI integer scores against each individual human rater's integer scores; and Formulation C, Gwet's AC2 (ordinal weights) of AI integer scores against the human composite rounded to the nearest integer. Substantive dimension-level conclusions are robust across the primary analysis and both sensitivity analyses.

Table E2.1. Three-formulation Gwet's AC2 (ordinal weights) and ICC, AI vs human raters and human composite

Dimension	AC2 H1×H2	AC2 AI×H1 (Form A)	AC2 AI×H2 (Form A)	AC2 AI×Rounded (Form C)	ICC H1×H2	ICC AI×Continuous (Form B)	ICC AI×Rounded (Form C)
Required Contents	0.589	−0.628	−0.482	−0.667	−0.006	−0.003	−0.008

Specifics	0.498	0.764	0.438	0.687	−0.101	0.031	0.029
Thoroughness	0.588	0.626	0.360	0.487	0.014	−0.058	−0.049
Clarity	0.599	0.523	0.440	0.376	−0.080	0.142	0.107
Skill	0.557	0.539	0.645	0.741	−0.075	0.115	0.188
Purity	0.419	0.745	0.639	0.762	−0.105	0.196	0.214

Table E2.2. ***Formulation A — AI vs each individual human rater (95% confidence intervals)***

Dimension	AC2 AI×R1 [95% CI]	AC2 AI×R2 [95% CI]
Required Contents	−0.628 [−0.865, −0.391]	−0.482 [−0.776, −0.188]
Specifics	0.764 [0.653, 0.875]	0.438 [0.227, 0.649]
Thoroughness	0.626 [0.485, 0.766]	0.360 [0.167, 0.552]
Clarity	0.523 [0.389, 0.657]	0.440 [0.233, 0.647]
Skill	0.539 [0.356, 0.722]	0.645 [0.511, 0.779]
Purity	0.745 [0.636, 0.853]	0.639 [0.477, 0.801]

Note. ICC = intraclass correlation coefficient (two-way random-effects, single-measures, absolute agreement). AC2 computed with ordinal weights. H1 = Human Rater 1; H2 = Human Rater 2; AI×Rounded = AI integer scores against the human composite rounded to the nearest integer.

Interpretation. Substantive conclusions are robust across formulations: dimensions reaching substantial agreement (Specifics, Skill, Purity; AC2 0.69–0.76 in Formulation C) and dimensions showing systematic AI under-rating (Required Contents) appear consistently regardless of whether the human reference is treated as discrete or continuous. ICCs collapse near zero across all formulations because of restriction-of-range / ceiling effects in the human reference distribution (see Supplement E4); AC2 with ordinal weights provides a more interpretable agreement metric for ceiling-clipped data.

E3. Demographic Subgroup Analyses

To address the editor's request that the AI system be tested for systematic demographic bias, AI minus human composite mean differences and Gwet's AC2 (ordinal weights, against the rounded human composite) were stratified by lay-provider sex (Female n = 39 sessions; Male n = 13 sessions) and fellow age band (median split at 20 years; under 20 n = 23 sessions; 20 or older n = 29 sessions). Statistical tests of differential bias used Welch's two-sample t-tests on the AI minus human composite difference per session.

Table E3.1. AI minus Human composite difference, stratified by lay-provider sex

Dimension	Mean diff Male (n=13)	Mean diff Female (n=39)	t (Welch)	p	AC2 Male	AC2 Female

**Required Contents**	−2.81	−2.95	0.40	.690	−0.213	**−0.580**
**Specifics**	−0.15	−0.32	0.47	.641	0.796	**0.804**
**Thoroughness**	−0.85	−1.01	0.39	.702	0.294	**0.628**
**Clarity**	−1.27	−1.44	0.63	.536	0.598	**0.589**
**Skill**	0.81	0.53	1.16	.258	0.617	**0.611**
**Purity**	**0.15**	**0.26**	**−0.19**	**.852**	**0.608**	**0.767**

Table E3.2. *AI minus Human composite difference, stratified by lay-provider age band (median split at 20)*

**Dimension**	**Mean diff <20 (n=23)**	**Mean diff ≥20 (n=29)**	**t (Welch)**	**p**	**AC2 <20**	**AC2 ≥20**
Required Contents	−2.61	−3.16	1.65	.105	−0.248	−0.672
Specifics	−0.15	−0.38	0.70	.486	0.792	0.813
Thoroughness	−0.63	−1.24	1.54	.130	0.600	0.599
Clarity	−1.15	−1.59	1.49	.143	0.470	0.578
Skill	0.67	0.53	0.56	.575	0.739	0.691
Purity	0.43	0.07	1.01	.318	0.595	0.651

Note. Mean diff = AI rating minus human composite (negative = AI rated lower; positive = AI rated higher). AC2 computed with ordinal weights against the rounded human composite. t = Welch's two-sample t-test on the AI–human difference.

Interpretation. No statistically significant sex effect on AI–human bias was observed on any of the six dimensions (all $p > .25$). No statistically significant age-band effect was observed on any of the six dimensions (all $p > .10$). Required Contents shows a marginal trend toward larger AI-under-human gap for older fellows ($\Delta = -0.55$, $p = .105$) but does not reach statistical significance. Dimension-level AC2 was qualitatively similar across strata.

E4. Ceiling-Effect Characterization of the Human Reference Standard

Human composite ratings clustered tightly near the ceiling, restricting variance and mechanically suppressing ICC values. Together with the human–human Gwet's AC2 of 0.42–0.60, this establishes the realistic upper bound on the agreement any model could achieve against this composite.

Table E4.1. Distribution of the human composite by fidelity dimension (N = 52 sessions)

**Dimension**	**Mean**	**SD**	**Min**	**Max**	**% rated ≥ 6**	**% rated ≥ 7**	**IQR**

Required Contents	6.14	0.74	5	7	59.6%	34.6%	[5.5, 7.0]
Specifics	5.82	0.71	4	7	51.9%	7.7%	[5.5, 6.5]
Thoroughness	5.78	0.70	4	7	51.9%	5.8%	[5.5, 6.5]
Clarity	5.89	0.64	4	7	55.8%	5.8%	[5.5, 6.5]
Skill	5.87	0.67	4	7	59.6%	7.7%	[5.5, 6.5]
Purity	6.08	0.58	5	7	73.1%	13.5%	[5.5, 6.5]

Note. Human composite = mean of two independent human raters per session. Restricted range (Min ≥ 4 across all dimensions; Min ≥ 5 for Required Contents and Purity) and high % at ceiling (51.9–73.1% rated ≥ 6) indicate substantial ceiling concentration. ICC, which assumes adequate variance in the reference, is mechanically suppressed in this distribution; AC2 with ordinal weights (and chance-correction adjusted for prevalence and bias) is the more interpretable agreement metric for ceiling-clipped data and is reported as the primary agreement statistic.

E5. Per-Arm Robustness Check

To examine whether the imbalance between the AI-augmented (n = 38 sessions) and standard supervision (n = 14 sessions) arms in the validation set introduced systematic bias, we compared AI rating means, human composite means, and per-arm Gwet's AC2 between the two arms for each of the six fidelity dimensions.

Table E5.1. ***Per-arm AI ratings, human composite ratings, and Welch's t-tests***

Dimension	AI: AI-arm M (SD)	AI: Std-arm M (SD)	t / p (AI)	Human: AI-arm M (SD)	Human: Std-arm M (SD)	t / p (Human)
Required Contents	3.18 (0.93)	3.36 (0.93)	−0.60 / .557	6.11 (0.74)	6.25 (0.78)	−0.60 / .552
Specifics	5.50 (0.92)	5.64 (1.01)	−0.46 / .648	5.79 (0.76)	5.89 (0.56)	−0.53 / .598
Thoroughness	4.68 (1.09)	5.14 (1.46)	−1.07 / .298	5.78 (0.73)	5.79 (0.61)	−0.05 / .963
Clarity	4.45 (1.03)	4.64 (1.28)	−0.51 / .613	5.88 (0.67)	5.93 (0.55)	−0.26 / .799
Skill	6.45 (0.65)	6.50 (0.85)	−0.21 / .836	5.86 (0.69)	5.89 (0.63)	−0.19 / .853
Purity	6.34 (1.34)	6.21 (1.53)	0.28 / .785	6.07 (0.62)	6.11 (0.49)	−0.25 / .803

Table E5.2. *Per-arm Gwet's AC2 (ordinal weights) against the rounded human composite*

Dimension	AC2 AI-arm (n = 38)	AC2 Std-arm (n = 14)
Required Contents	−0.679	−0.596
Specifics	0.666	0.753
Thoroughness	0.483	0.535
Clarity	0.394	0.034
Skill	0.748	0.728
Purity	0.788	0.688

Note. AI = shamiriAI ratings. Human = human composite (mean of two raters). t = Welch's two-sample t-test comparing arms.

Interpretation. AI rating means did not differ significantly between arms on any of the six dimensions (all $p > .29$), and human composite means similarly did not differ (all $p > .55$). Per-arm AC2 followed the same dimension-dependent pattern as the overall analysis for five of six dimensions. Clarity AC2 was lower in the standard arm (0.034 vs 0.394 in the AI-augmented arm), an asymmetry attributable to the small standard-arm sample (n = 14) rather than a substantive arm-level difference, given the absence of mean-level differences for Clarity (all $p > .61$). The arm imbalance does not appear to introduce systematic bias in the overall AI–human agreement findings. The need for arm-balanced sampling in future validation work is acknowledged in the Limitations section of the main manuscript.

References (Supplements)

1. Osborn TL, Venturo-Conerly KE, Arango G. S, Roe E, Rodriguez M, Alemu RG, Gan J, Wasil AR, Otieno BH, Rusch T, Ndetei DM, Wasanga C, Schleider JL, Weisz JR. Effect of Shamiri Layperson-Provided Intervention vs Study Skills Control Intervention for Depression and Anxiety Symptoms in Adolescents in Kenya: A Randomized Clinical Trial. JAMA Psychiatry 2021 June 9; doi: 10.1001/jamapsychiatry.2021.1129

2. Osborn TL, Venturo-Conerly KE, Wasil AR, Rodriguez M, Roe E, Alemu R, Arango G. S, Gan J, Wasanga C, Schleider JL, Weisz JR. The Shamiri group intervention for adolescent anxiety and depression: study protocol for a randomized controlled trial of a lay-provider-delivered, school-based intervention in Kenya. Trials 2020 Nov 23;21(1):938. doi: 10.1186/s13063-020-04732-1

3. Osborn TL, Wasil AR, Venturo-Conerly KE, Schleider JL, Weisz JR. Group Intervention for Adolescent Anxiety and Depression: Outcomes of a Randomized Trial with Adolescents in Kenya. Behav Ther 2020 July 1;51(4):601–615. doi: 10.1016/j.beth.2019.09.005

4. Schleider JL, Mullarkey MC, Chacko A. Harnessing Wise Interventions to Advance the Potency and Reach of Youth Mental Health Services. Clin Child Fam Psychol Rev 2020 Mar;23(1):70–101. doi: 10.1007/s10567-019-00301-4

5. Walton GM. The New Science of Wise Psychological Interventions. Curr Dir Psychol Sci 2014 Feb 1;23(1):73–82. doi: 10.1177/0963721413512856

6. Walton GM, Wilson TD. Wise interventions: Psychological remedies for social and personal problems. Psychol Rev 2018 Oct;125(5):617–655. doi: 10.1037/rev0000115

7. Yeager DS, Dweck CS. Mindsets That Promote Resilience: When Students Believe That Personal Characteristics Can Be Developed. Educ Psychol 2012 Oct 1;47(4):302–314. doi: 10.1080/00461520.2012.722805

8. Dweck CS. Mindset: The new psychology of success. Random House Digital, Inc.; 2008.

9. Froh JJ, Sefick WJ, Emmons RA. Counting blessings in early adolescents: An experimental study of gratitude and subjective well-being. J Sch Psychol 2008 Apr;46(2):213–233. doi: 10.1016/j.jsp.2007.03.005

10. Froh JJ, Kashdan TB, Ozimkowski KM, Miller N. Who benefits the most from a gratitude intervention in children and adolescents? Examining positive affect as a moderator. J Posit Psychol 2009 Sept;4(5):408–422. doi: 10.1080/17439760902992464

11. Cohen GL, Garcia J, Purdie-Vaughns V, Apfel N, Brzustoski P. Recursive Processes in Self-Affirmation: Intervening to Close the Minority Achievement Gap. Science 2009 Apr 17;324(5925):400–403. doi: 10.1126/science.1170769

12. Miyake A, Kost-Smith LE, Finkelstein ND, Pollock SJ, Cohen GL, Ito TA. Reducing the Gender Achievement Gap in College Science: A Classroom Study of Values Affirmation. Science 2010 Nov 26;330(6008):1234–1237. doi: 10.1126/science.1195996

13. Venturo-Conerly KE, Roe E, Wasil A, Osborn T, Ndetei D, Musyimi C, Mutiso V, Wasanga C, Weisz JR. Training and Supervising Lay Providers in Kenya: Strategies and Mixed-Methods Outcomes☆☆☆. Cogn Behav Pract 2021 May 5; doi: 10.1016/j.cbpra.2021.03.004

14. Schleider JL, Weisz JR. A single-session growth mindset intervention for adolescent anxiety and depression: 9-month outcomes of a randomized trial. J Child Psychol Psychiatry 2018;59(2):160–170. doi: 10.1111/jcpp.12811

15. Kahi S, Memba L, Syan A, Ngatia V, Venturo-Conerly K, Wasanga C, Osborn TL. Implementation of a school-based risk management protocol within a task-shifted mental healthcare model. Camb Prisms Glob Ment Health 2025 Jan;12:e127. doi: 10.1017/gmh.2025.10073

16. Gwet KL. Handbook of inter-rater reliability: The definitive guide to measuring the extent of agreement among raters. Advanced Analytics, LLC; 2014.

17. Gwet KL. Computing inter-rater reliability and its variance in the presence of high agreement. Br J Math Stat Psychol 2008 May;61(1):29–48. doi: 10.1348/000711006X126600

18. Radford A, Kim JW, Xu T, Brockman G, Mcleavey C, Sutskever I. Robust Speech Recognition via Large-Scale Weak Supervision. Proc 40th Int Conf Mach Learn PMLR; 2023. p. 28492–28518. Available from: https://proceedings.mlr.press/v202/radford23a.html [accessed May 12, 2026]

19. Rouditchenko A, Khurana S, Thomas S, Feris R, Karlinsky L, Kuehne H, Harwath D, Kingsbury B, Glass J. Comparison of Multilingual Self-Supervised and Weakly-Supervised Speech Pre-Training for Adaptation to Unseen Languages. arXiv; 2023. doi: 10.48550/arXiv.2305.12606

20. Peng P, Yan B, Watanabe S, Harwath D. Prompting the Hidden Talent of Web-Scale Speech Models for Zero-Shot Task Generalization. arXiv; 2023. doi: 10.48550/arXiv.2305.11095

21. Bredin H. pyannote.audio 2.1 speaker diarization pipeline: principle, benchmark, and recipe. INTERSPEECH 2023 ISCA; 2023. p. 1983–1987. doi: 10.21437/Interspeech.2023-105

22. Plaquet A, Bredin H. Powerset multi-class cross entropy loss for neural speaker diarization. INTERSPEECH 2023 ISCA; 2023. p. 3222–3226. doi: 10.21437/Interspeech.2023-205

23. Zaratiana U, Tomeh N, Holat P, Charnois T. GLiNER: Generalist Model for Named Entity Recognition using Bidirectional Transformer. arXiv; 2023. doi: 10.48550/arXiv.2311.08526

24. Landis JR, Koch GG. The Measurement of Observer Agreement for Categorical Data. Biometrics 1977 Mar;33(1):159. doi: 10.2307/2529310
