# Supplementary material for: Automated Fidelity Monitoring of Lay-Delivered Mental Health Interventions Using Large Language Models: Development and Pilot Validation of shamiriAI in Kenya
Source: JMIR AI. 2026 Jul 23;5:e95063. doi: 10.2196/95063 (PMC13396917; doi:10.2196/95063)
Supplement: Checklist 1 [file ai-v5-e95063-s002.pdf]

TRIPOD+LLM Checklist

Section / Topic	Item	Checklist Item	Research Design	LLM Task	Reported on Page
**Abstract**					
Title	2a	Identify the study as developing, fine-tuning, and/or evaluating the performance of an LLM, specifying the task, the target population, and the outcome to be predicted.	All	All	Title (p.1)
Abstract	2b	Provide a brief explanation of the healthcare context, use case and rationale for developing or evaluating the performance of an LLM.	E,H	All	Abstract (p.1)
Objectives	2c	Specify the study objectives, including whether the study describes LLMs development, tuning, and/or evaluation.	All	All	Abstract (p.1)
Methods	2d	Describe the key elements of the study setting.	All	All	Abstract (p.1)
	2e	Detail all data used in the study, specify data splits and any selective use of data.	M,D,E	All	Not Required
	2f	Specify the name and version of LLM used.	All	All	Abstract (p.1)
	2g	Briefly summarize the LLM-building steps, including any fine-tuning, reward modeling, RLHF, etc.	M,D	All	Not Required
	2h	Describe the specific tasks performed by the LLMs (e.g., QA, summarization, extraction), highlighting key inputs and outputs.	All	All	Abstract (p.1)
	2i	Specify the evaluation datasets/populations used, including the endpoint evaluated, whether held out during training/tuning, and what measure(s) were used.	All	All	Abstract (p.1)
Results	2j	Give an overall report and interpretation of the main results.	All	All	Abstract (p.1)
Discussion	2k	Explicitly state any broader implications or concerns arising from these results.	All	All	Abstract (p.1)
Other	2l	Give the registration number and name of the registry or repository (if relevant).	H	All	Abstract (p.1)
**Introduction**					
Background	3a	Explain the healthcare context / use case and rationale for developing or evaluating the LLM, including references to existing approaches and models.	All	All	3-4
	3b	Describe the target population and the intended use of the LLM in the care pathway, including its intended users.	E,H	All	3-4
Objectives	4	Specify the study objectives, including whether the study describes initial development, fine-tuning, or validation of an LLM.	All	All	4-5
**Methods**					
Data	5a	Describe the sources of data separately for training, tuning, and/or evaluation datasets and the rationale for using these data.	All	All	5-7
	5b	Describe the relevant data points and provide a quantitative and qualitative description of their distribution and descriptors (source, languages, countries).	All	All	5-7
	5c	Specifically state the date of the oldest and newest item of text used in development and in the evaluation datasets.	M,D,E,H	All	5-7
	5d	Describe any data pre-processing and quality checking, including whether similar across corpora, institutions, and sociodemographic groups.	All	All	7-11
	5e	Describe how missing and imbalanced data were handled and provide reasons for omitting any data.	M,D,E	All	Not Required
Analytical Methods	6a	Report the LLM name, version, and last date of training or use during inference.	All	All	12-14
	6b	Specify the LLM architecture and building steps, including hyperparameter tuning and inference settings (seed, temperature, max token length).	M,D,E	All	Not Required
	6c	Report details of the LLM development process from input to outcome generation (training, fine-tuning, alignment strategy and goals).	M,D	All	9-11
	6d	Specify the initial and post-processed output of the LLM (e.g., probabilities, classification, unstructured text).	All	All	9-11
	6e	Provide details and rationale for any classification and how probabilities/thresholds were determined.	All	C,OF	12-14
	6f	Include metrics that capture the quality of generative outputs (consistency, relevance, accuracy) vs gold	All	QA,IR,DG,SS,MT	9-15

		standards.			
	6g	Report the outcome metrics' relevance to the downstream task and correlation to human evaluation for intended use.	E,H	All	9-15
LLM Output	7a	Clearly define the outcome, how LLM predictions were calculated (formula, code, object, API), and evaluation metrics.	E,H	All	12-15
	7b	If outcome assessment requires subjective interpretation, describe assessor qualifications, instructions, demographics, and inter-assessor agreement.	All	All	NA
	7c	Specify how performance was compared to other LLMs, humans, and other benchmarks or standards.	All	All	22-24
Annotation	8a	If annotation was done, report how text was labeled, including specific annotation guidelines with examples.	All	All	NA
	8b	If annotation was done, report how many annotators labeled the dataset(s) and the proportion double-annotated.	All	All	NA
	8c	If annotation was done, provide annotator background/experience and inter-annotator agreement.	All	All	NA
Prompting	9a	If prompting LLMs, provide details on prompt design, curation, and selection.	All	All	12-14
	9b	If prompting LLMs, report what data were used to develop the prompts.	All	All	12-14
Summarization	10	Describe any preprocessing of the data before summarization.	All	SS	Not Required
Instruction Tuning / Alignment	11	If instruction tuning/alignment strategies were used, what were the instructions and interface for evaluation, and the populations doing evaluation?	M,D	All	Not Required
Compute	12	Report compute, or proxies thereof (time/cost on what and how many machines, inference time, FLOPs), required to carry out methods.	M,D,E	All	**9-11**
Ethics Approval	13	Name the IRB/ethics committee that approved the study and describe participant informed consent or the waiver.	All	All	5
**Open Science**					
Open Science	14a	Give the source of funding and the role of the funders for the present study.	All	All	27
	14b	Declare any conflicts of interest and financial disclosures for all authors.	All	All	27
	14c	Indicate where the study protocol can be accessed or state that a protocol was not prepared.	H	All	2
	14d	Provide registration information (register name and number) or state that the study was not registered.	H	All	5
	14e	Provide details of the availability of the study data.	All	All	14
	14f	Provide details of the availability of the code to reproduce the study results.	All	All	14
Public Involvement	15	Provide details of any patient and public involvement, or state no involvement.	H	All	NA
**Results**					
Participants	16a	When using patient/EHR data, describe the flow of data through the study, including counts with/without outcome and follow-up time.	E,H	All	NA
	16b	When using patient/EHR data, report characteristics overall and per source/setting and dev/eval splits, including key dates, predictors, sample size.	E,H	All	NA
	16c	For LLM evaluation, show a comparison of the distribution of important predictors between development and evaluation data.	E,H	All	NA
	16d	When using patient/EHR data, specify the number of participants and outcome events in each analysis.	E,H	All	NA
Performance	17	Report LLM performance according to pre-specified metrics (item 7a) and/or human evaluation (item 7d).	All	All	15-22
LLM Updating	18	If applicable, report results from any LLM updating, including the updated LLM and subsequent performance.	All	All	NA
**Discussion**					
Interpretation	19a	Give an overall interpretation of the main results, including issues of fairness in the context of the objectives and previous studies.	All	All	22-24
Limitations	19b	Discuss any limitations of the study and their effects on biases, statistical uncertainty, and generalizability.	All	All	24-25
Usability of the LLM in context	19c	Describe known challenges in using data for the task/domain with reference to representation, missingness, harmonization, and bias.	E,H	All	22-25
	19d	Define the intended use for the implementation under evaluation, including intended input, end-user, level of autonomy/human oversight.	E,H	All	22-25

	19e	If applicable, describe how poor quality or unavailable input data should be assessed and handled when implementing the LLM.	E,H	All	NA
	19f	If applicable, specify whether users interact in handling input data or use of the LLM, and the expertise required.	E,H	All	NA
	19g	Discuss next steps for future research, with a view to applicability and generalizability of the LLM.	All	All	25
