# Supplementary material for: Automated Fidelity Monitoring of Lay-Delivered Mental Health Interventions Using Large Language Models: Development and Pilot Validation of shamiriAI in Kenya
Source: JMIR AI. 2026 Jul 23;5:e95063. doi: 10.2196/95063 (PMC13396917; doi:10.2196/95063)
Supplement: Checklist 2 [file ai-v5-e95063-s003.pdf]

CONSORT-EHEALTH (V 1.6.1) - Submission/Publication Form

The CONSORT-EHEALTH checklist is intended for authors of randomized trials evaluating web-based and Internet-based applications/interventions, including mobile interventions, electronic games (incl multiplayer games), social media, certain telehealth applications, and other interactive and/or networked electronic applications. Some of the items (e.g. all subitems under item 5 - description of the intervention) may also be applicable for other study designs.

The goal of the CONSORT EHEALTH checklist and guideline is to be
a) a guide for reporting for authors of RCTs,
b) to form a basis for appraisal of an ehealth trial (in terms of validity)

CONSORT-EHEALTH items/subitems are MANDATORY reporting items for studies published in the Journal of Medical Internet Research and other journals / scientific societies endorsing the checklist.

Items numbered 1., 2., 3., 4a., 4b etc are original CONSORT or CONSORT-NPT (non-pharmacologic treatment) items.
Items with Roman numerals (i., ii, iii, iv etc.) are CONSORT-EHEALTH extensions/clarifications.

As the CONSORT-EHEALTH checklist is still considered in a formative stage, we would ask that you also RATE ON A SCALE OF 1-5 how important/useful you feel each item is FOR THE PURPOSE OF THE CHECKLIST and reporting guideline (optional).

Mandatory reporting items are marked with a red *.
In the textboxes, either copy & paste the relevant sections from your manuscript into this form - please include any quotes from your manuscript in QUOTATION MARKS,
or answer directly by providing additional information not in the manuscript, or elaborating on why the item was not relevant for this study.

YOUR ANSWERS WILL BE PUBLISHED AS A SUPPLEMENTARY FILE TO YOUR PUBLICATION IN JMIR AND ARE CONSIDERED PART OF YOUR PUBLICATION (IF ACCEPTED).
Please fill in these questions diligently. Information will not be copyedited, so please use proper spelling and grammar, use correct capitalization, and avoid abbreviations.

DO NOT FORGET TO SAVE AS PDF _AND_ CLICK THE SUBMIT BUTTON SO YOUR ANSWERS ARE IN OUR DATABASE !!!

Citation Suggestion (if you append the pdf as Appendix we suggest to cite this paper in the caption):
Eysenbach G, CONSORT-EHEALTH Group
CONSORT-EHEALTH: Improving and Standardizing Evaluation Reports of Web-based and Mobile Health Interventions
J Med Internet Res 2011;13(4):e126

URL: http://www.jmir.org/2011/4/e126/
doi: 10.2196/jmir.1923
PMID: 22209829

rachael.kilonzo@shamiri.institute Switch account

Draft not saved

Not shared

* Indicates required question

Your name *

First Last

Tom Osborn

Primary Affiliation (short), City, Country *

University of Toronto, Toronto, Canada

Shamiri, Nairobi, Kenya

Your e-mail address *

abc@gmail.com

osborn@shamiri.institute

Title of your manuscript *

Provide the (draft) title of your manuscript.

Automated Fidelity Monitoring of Lay-Delivered Mental Health Interventions Using Large Language Models: Development and Pilot Validation of shamiriAI

Name of your App/Software/Intervention *

If there is a short and a long/alternate name, write the short name first and add the long name in brackets.

shamiriAI

Evaluated Version (if any)

e.g. "V1", "Release 2017-03-01", "Version 2.0.27913"

Your answer

Language(s) *

What language is the intervention/app in? If multiple languages are available, separate by comma (e.g. "English, French")

English

URL of your Intervention Website or App

e.g. a direct link to the mobile app on app in appstore (itunes, Google Play), or URL of the website. If the intervention is a DVD or hardware, you can also link to an Amazon page.

https://huggingface.co/shamiri-ai/models

URL of an image/screenshot (optional)

Your answer

Accessibility *

Can an enduser access the intervention presently?

- ○ access is free and open
- ◉ access only for special usergroups, not open
- ○ access is open to everyone, but requires payment/subscription/in-app purchases
- ○ app/intervention no longer accessible
- ○ Other:

Primary Medical Indication/Disease/Condition *

e.g. "Stress", "Diabetes", or define the target group in brackets after the condition, e.g. "Autism (Parents of children with)", "Alzheimers (Informal Caregivers of)"

Adolescent mental health (depression, anxiety,

Primary Outcomes measured in trial *

comma-separated list of primary outcomes reported in the trial

ASR performance (Character Error Rate, Word

Secondary/other outcomes

Are there any other outcomes the intervention is expected to affect?

The intervention is expected to affect provider skill, intervention fidelity at scale, and potentially student mental health outcomes in future effectiveness trials, though these were not primary outcomes measured in this pilot validation. The pilot also assessed systematic bias in AI ratings and performed demographic subgroup and per-arm robustness checks.

Recommended "Dose" *

What do the instructions for users say on how often the app should be used?

- ( ) Approximately Daily
- (x) Approximately Weekly
- ( ) Approximately Monthly
- ( ) Approximately Yearly
- ( ) "as needed"
- ( ) Other:

Approx. Percentage of Users (starters) still using the app as recommended after 3 months *

- ( ) unknown / not evaluated
- ( ) 0-10%
- ( ) 11-20%
- ( ) 21-30%
- ( ) 31-40%
- ( ) 41-50%
- ( ) 51-60%
- ( ) 61-70%
- ( ) 71%-80%
- ( ) 81-90%
- ( ) 91-100%
- (x) Other: None

Overall, was the app/intervention effective? *

- ○ yes: all primary outcomes were significantly better in intervention group vs control
- ○ partly: SOME primary outcomes were significantly better in intervention group vs control
- ○ no statistically significant difference between control and intervention
- ○ potentially harmful: control was significantly better than intervention in one or more outcomes
- ○ inconclusive: more research is needed
- ◉ Other: This study is a development and pilot validation of an AI-based tool, n

Article Preparation Status/Stage *

At which stage in your article preparation are you currently (at the time you fill in this form)

- ○ not submitted yet - in early draft status
- ○ not submitted yet - in late draft status, just before submission
- ○ submitted to a journal but not reviewed yet
- ◉ submitted to a journal and after receiving initial reviewer comments
- ○ submitted to a journal and accepted, but not published yet
- ○ published
- ○ Other:

Journal *

If you already know where you will submit this paper (or if it is already submitted), please provide the journal name (if it is not JMIR, provide the journal name under "other")

- ○ not submitted yet / unclear where I will submit this
- ○ Journal of Medical Internet Research (JMIR)
- ○ JMIR mHealth and UHealth
- ○ JMIR Serious Games
- ○ JMIR Mental Health
- ○ JMIR Public Health
- ○ JMIR Formative Research
- ○ Other JMIR sister journal
- ◉ Other: JMIR AI

Is this a full powered effectiveness trial or a pilot/feasibility trial? *

- ◉ Pilot/feasibility
- ○ Fully powered

Manuscript tracking number *

If this is a JMIR submission, please provide the manuscript tracking number under "other" (The ms tracking number can be found in the submission acknowledgement email, or when you login as author in JMIR. If the paper is already published in JMIR, then the ms tracking number is the four-digit number at the end of the DOI, to be found at the bottom of each published article in JMIR)

- ○ no ms number (yet) / not (yet) submitted to / published in JMIR
- ◉ Other: JAI ms#95063

TITLE AND ABSTRACT

1a) TITLE: Identification as a randomized trial in the title

1a) Does your paper address CONSORT item 1a? *

I.e does the title contain the phrase "Randomized Controlled Trial"? (if not, explain the reason under "other")

- ○ yes
- ◉ Other: The manuscript title is "Automated Fidelity Monitoring of Lay-Delivere

1a-i) Identify the mode of delivery in the title

Identify the mode of delivery. Preferably use “web-based” and/or “mobile” and/or “electronic game” in the title. Avoid ambiguous terms like “online”, “virtual”, “interactive”. Use “Internet-based” only if Intervention includes non-web-based Internet components (e.g. email), use “computer-based” or “electronic” only if offline products are used. Use “virtual” only in the context of “virtual reality” (3-D worlds). Use “online” only in the context of “online support groups”. Complement or substitute product names with broader terms for the class of products (such as “mobile” or “smart phone” instead of “iphone”), especially if the application runs on different platforms.

	1	2	3	4	5	
subitem not at all important	○	○	○	○	◉	essential

Clear selection

Does your paper address subitem 1a-i? *

Copy and paste relevant sections from manuscript title (include quotes in quotation marks "like this" to indicate direct quotes from your manuscript), or elaborate on this item by providing additional information not in the ms, or briefly explain why the item is not applicable/relevant for your study

The title explicitly mentions "Automated Fidelity Monitoring" and "Large Language Models," indicating the AI/electronic nature of the intervention. While "web-based" or "mobile" are not used, the description clearly points to an electronic/AI-based mode of delivery.

1a-ii) Non-web-based components or important co-interventions in title

Mention non-web-based components or important co-interventions in title, if any (e.g., “with telephone support”).

	1	2	3	4	5	
subitem not at all important	○	○	◉	○	○	essential

Clear selection

Does your paper address subitem 1a-ii?

Copy and paste relevant sections from manuscript title (include quotes in quotation marks "like this" to indicate direct quotes from your manuscript), or elaborate on this item by providing additional information not in the ms, or briefly explain why the item is not applicable/relevant for your study

Not explicitly mentioned in the title. The manuscript describes shamiriAI as providing "structured feedback reports" to "clinical supervisors" who then "guide discussions with lay providers." This implies human co-intervention as part of the supervision process, but it is not in the title.

1a-iii) Primary condition or target group in the title

Mention primary condition or target group in the title, if any (e.g., “for children with Type I Diabetes”) Example: A Web-based and Mobile Intervention with Telephone Support for Children with Type I Diabetes: Randomized Controlled Trial

	1	2	3	4	5	
subitem not at all important	○	○	○	○	◉	essential

Clear selection

Does your paper address subitem 1a-iii? *

Copy and paste relevant sections from manuscript title (include quotes in quotation marks "like this" to indicate direct quotes from your manuscript), or elaborate on this item by providing additional information not in the ms, or briefly explain why the item is not applicable/relevant for your study

"Lay-Delivered Mental Health Interventions," which implies the target group receiving the intervention (adolescents) and the broader field of mental health. The abstract further clarifies "adolescent mental health" as the primary target.

1b) ABSTRACT: Structured summary of trial design, methods, results, and conclusions

NPT extension: Description of experimental treatment, comparator, care providers, centers, and blinding status.

1b-i) Key features/functionalities/components of the intervention and comparator in the METHODS section of the ABSTRACT

Mention key features/functionalities/components of the intervention and comparator in the abstract. If possible, also mention theories and principles used for designing the site. Keep in mind the needs of systematic reviewers and indexers by including important synonyms. (Note: Only report in the abstract what the main paper is reporting. If this information is missing from the main body of text, consider adding it)

	1	2	3	4	5	
subitem not at all important	○	○	○	○	◉	essential

Clear selection

Does your paper address subitem 1b-i? *

Copy and paste relevant sections from the manuscript abstract (include quotes in quotation marks "like this" to indicate direct quotes from your manuscript), or elaborate on this item by providing additional information not in the ms, or briefly explain why the item is not applicable/relevant for your study

"shamiriAI processed session audio through a five-stage pipeline: ingestion, multilingual automatic speech recognition (ASR) with prosodic feature extraction, personally identifiable information (PII) scrubbing, large language model (LLM)-based fidelity inference, and structured supervision reports."

Describes the core functionalities of shamiriAI and its pipeline. It also implicitly defines the comparator as "independent human supervisor ratings" against which shamiriAI's ratings are compared for interrater reliability.

1b-ii) Level of human involvement in the METHODS section of the ABSTRACT

Clarify the level of human involvement in the abstract, e.g., use phrases like “fully automated” vs. “therapist/nurse/care provider/physician-assisted” (mention number and expertise of providers involved, if any). (Note: Only report in the abstract what the main paper is reporting. If this information is missing from the main body of text, consider adding it)

	1	2	3	4	5	
subitem not at all important	○	○	○	○	◉	essential

Clear selection

Does your paper address subitem 1b-ii?

Copy and paste relevant sections from the manuscript abstract (include quotes in quotation marks "like this" to indicate direct quotes from your manuscript), or elaborate on this item by providing additional information not in the ms, or briefly explain why the item is not applicable/relevant for your study

The abstract clarifies the role of human supervisors: "interrater reliability between shamiriAI-generated fidelity ratings and independent human supervisor ratings across 52 recorded sessions". It also mentions "lay-delivered mental health interventions", indicating the human delivery of the primary intervention being monitored. For shamiriAI specifically, it's an "automated fidelity monitoring tool".

1b-iii) Open vs. closed, web-based (self-assessment) vs. face-to-face assessments in the METHODS section of the ABSTRACT

Mention how participants were recruited (online vs. offline), e.g., from an open access website or from a clinic or a closed online user group (closed usergroup trial), and clarify if this was a purely web-based trial, or there were face-to-face components (as part of the intervention or for assessment). Clearly say if outcomes were self-assessed through questionnaires (as common in web-based trials). Note: In traditional offline trials, an open trial (open-label trial) is a type of clinical trial in which both the researchers and participants know which treatment is being administered. To avoid confusion, use “blinded” or “unblinded” to indicated the level of blinding instead of “open”, as “open” in web-based trials usually refers to “open access” (i.e. participants can self-enrol). (Note: Only report in the abstract what the main paper is reporting. If this information is missing from the main body of text, consider adding it)

	1	2	3	4	5	
subitem not at all important	○	○	○	○	◉	essential

Clear selection

Does your paper address subitem 1b-iii?

Copy and paste relevant sections from the manuscript abstract (include quotes in quotation marks "like this" to indicate direct quotes from your manuscript), or elaborate on this item by providing additional information not in the ms, or briefly explain why the item is not applicable/relevant for your study

"Across six secondary schools in Ngong Hub, Kajiado County, Kenya" and "lay-delivered mental health interventions" which implies face-to-face sessions being recorded and then processed by the AI. The study itself involved human supervisors rating sessions, and the AI ratings were compared to these human ratings. This was not a purely web-based trial for participants, it involved real-world school settings.

1b-iv) RESULTS section in abstract must contain use data

Report number of participants enrolled/assessed in each group, the use/uptake of the intervention (e.g., attrition/adherence metrics, use over time, number of logins etc.), in addition to primary/secondary outcomes. (Note: Only report in the abstract what the main paper is reporting. If this information is missing from the main body of text, consider adding it)

	1	2	3	4	5	
subitem not at all important	○	○	○	○	◉	essential

Clear selection

Does your paper address subitem 1b-iv?

Copy and paste relevant sections from the manuscript abstract (include quotes in quotation marks "like this" to indicate direct quotes from your manuscript), or elaborate on this item by providing additional information not in the ms, or briefly explain why the item is not applicable/relevant for your study

"Across 52 recorded sessions (38 AI-augmented arm; 14 standard arm), spanning six domains..." and "Within this 52-session pilot, shamiriAI demonstrates technically feasible multilingual ASR and a coherent, dimension-dependent reliability profile." The abstract does not explicitly report attrition/adherence metrics for shamiriAI itself as it is atool for supervisors, but it clearly states the number of sessions processed and evaluated.

1b-v) CONCLUSIONS/DISCUSSION in abstract for negative trials

Conclusions/Discussions in abstract for negative trials: Discuss the primary outcome - if the trial is negative (primary outcome not changed), and the intervention was not used, discuss whether negative results are attributable to lack of uptake and discuss reasons. (Note: Only report in the abstract what the main paper is reporting. If this information is missing from the main body of text, consider adding it)

	1	2	3	4	5	
subitem not at all important	○	○	○	○	◉	essential

Clear selection

Does your paper address subitem 1b-v?

Copy and paste relevant sections from the manuscript abstract (include quotes in quotation marks "like this" to indicate direct quotes from your manuscript), or elaborate on this item by providing additional information not in the ms, or briefly explain why the item is not applicable/relevant for your study

"underperformance on holistic dimensions (Required Contents, Clarity) reflects diagnosable misalignments in rubric interpretation and prompt design." This addresses limitations and reasons for underperformance of results on specific dimensions.

INTRODUCTION

2a) In INTRODUCTION: Scientific background and explanation of rationale

2a-i) Problem and the type of system/solution

Describe the problem and the type of system/solution that is object of the study: intended as stand-alone intervention vs. incorporated in broader health care program? Intended for a particular patient population? Goals of the intervention, e.g., being more cost-effective to other interventions, replace or complement other solutions? (Note: Details about the intervention are provided in “Methods” under 5)

	1	2	3	4	5	
subitem not at all important	○	○	○	○	◉	essential

Clear selection

Does your paper address subitem 2a-i? *

Copy and paste relevant sections from the manuscript (include quotes in quotation marks "like this" to indicate direct quotes from your manuscript), or elaborate on this item by providing additional information not in the ms, or briefly explain why the item is not applicable/relevant for your study

"Background: Task-shifting—the delivery of evidence-based mental health interventions by trained nonprofessionals—has shown promise in closing the treatment gap in low- and middleincome countries. But its effectiveness depends critically on ongoing supervision—which is difficult to scale. Artificial intelligence (AI) tools that automatically process session recordings and generate structured fidelity feedback could offer a scalable alternative. To our knowledge, no such system has been developed or validated for lay-delivered, multilingual, group-format interventions in low-resource settings."

2a-ii) Scientific background, rationale: What is known about the (type of) system

Scientific background, rationale: What is known about the (type of) system that is the object of the study (be sure to discuss the use of similar systems for other conditions/diagnoses, if appropiate), motivation for the study, i.e. what are the reasons for and what is the context for this specific study, from which stakeholder viewpoint is the study performed, potential impact of findings [2]. Briefly justify the choice of the comparator.

	1	2	3	4	5	
subitem not at all important	○	○	○	○	◉	essential

Clear selection

Does your paper address subitem 2a-ii? *

Copy and paste relevant sections from the manuscript (include quotes in quotation marks "like this" to indicate direct quotes from your manuscript), or elaborate on this item by providing additional information not in the ms, or briefly explain why the item is not applicable/relevant for your study

"The second is the emergence of AI tools capable of automating the observational and feedback functions of supervision. Advances in automatic speech recognition (ASR), natural language processing, and large language models (LLMs) have made it technically feasible to process session audio, estimate provider behaviors, score fidelity to clinical protocols, and generate structured feedback reports — possibly replacing the manual review bottleneck with systematic, scalable monitoring. Research teams in high-income countries have developed AI systems that achieve human-level reliability on session quality ratings for individual cognitive behavioral therapy in Englis, and recent work demonstrates that LLMs can score therapeutic constructs from transcribed sessions with strong psychometric properties and valid associations with outcomes. The trajectory suggests AI-assisted fidelity monitoring is becoming viable but viable under a specific set of conditions: individual therapy, adult populations, professional providers, and English-language settings in high-income countries."

2b) In INTRODUCTION: Specific objectives or hypotheses

Does your paper address CONSORT subitem 2b? *

Copy and paste relevant sections from the manuscript (include quotes in quotation marks "like this" to indicate direct quotes from your manuscript), or elaborate on this item by providing additional information not in the ms, or briefly explain why the item is not applicable/relevant for your study

"Objective: We developed and pilot-validated shamiriAI, an automated fidelity monitoring tool for lay-delivered mental health interventions, embedded within the Shamiri school-based program in Kenya."
"We pursued two pilot aims. The first was to evaluate the performance of shamiriAI's multilingual ASR pipeline on a held-out test set of manually transcribed sessions... The second was to assess interrater reliability between shamiriAI-generated fidelity ratings and independent human supervisor ratings across six fidelity dimensions..."

METHODS

3a) Description of trial design (such as parallel, factorial) including allocation ratio

Does your paper address CONSORT subitem 3a? *

Copy and paste relevant sections from the manuscript (include quotes in quotation marks "like this" to indicate direct quotes from your manuscript), or elaborate on this item by providing additional information not in the ms, or briefly explain why the item is not applicable/relevant for your study

"We conducted a pilot validation study of shamiriAI, an AI-based supervision support tool, embedded within routine delivery of the Shamiri intervention across six secondary schools in Ngong Hub, Kajiado County, Kenya, between May and September 2025. The study evaluated two pilot aims: (1) the technical performance of shamiriAI's automatic speech recognition (ASR) pipeline on a held-out test set of manually transcribed sessions, and (2) interrater reliability between shamiriAI-generated fidelity ratings and independent human supervisor ratings across 52 recorded sessions. These aims concern the technical properties of the system rather than between-arm comparisons."

3b) Important changes to methods after trial commencement (such as eligibility criteria), with reasons

Does your paper address CONSORT subitem 3b? *

Copy and paste relevant sections from the manuscript (include quotes in quotation marks "like this" to indicate direct quotes from your manuscript), or elaborate on this item by providing additional information not in the ms, or briefly explain why the item is not applicable/relevant for your study

"Prompt development artefacts were committed to version control together at project handoff rather than incrementally, so file-level timestamps do not individually reflect chronological order."
Prompt refinements across versions were made prior to the validation study, ensuring a "clean held-out evaluation."
"The final prompt (iteration_three) was locked prior to the assembly and scoring of the 52-session validation dataset." This indicates that changes to the core AI system's prompts were completed before the commencement of the specific validation reported in this page

3b-i) Bug fixes, Downtimes, Content Changes

Bug fixes, Downtimes, Content Changes: ehealth systems are often dynamic systems. A description of changes to methods therefore also includes important changes made on the intervention or comparator during the trial (e.g., major bug fixes or changes in the functionality or content) (5-iii) and other “unexpected events” that may have influenced study design such as staff changes, system failures/downtimes, etc. [2].

	1	2	3	4	5	
subitem not at all important	○	○	◉	○	○	essential

Clear selection

Does your paper address subitem 3b-i?

Copy and paste relevant sections from the manuscript (include quotes in quotation marks "like this" to indicate direct quotes from your manuscript), or elaborate on this item by providing additional information not in the ms, or briefly explain why the item is not applicable/relevant for your study

The final prompt (iteration_three) was "locked prior to the assembly and scoring of the 52-session validation dataset," implying that major content or functionality changes to the AI system itself were not made during the reported pilot validation phase. "Prompt development artefacts were committed to version control together at project handoff rather than incrementally, so file-level timestamps do not individually reflect chronological order." This suggests development history but no dynamic changes during the reported trial period.

4a) Eligibility criteria for participants

Does your paper address CONSORT subitem 4a? *

Copy and paste relevant sections from the manuscript (include quotes in quotation marks "like this" to indicate direct quotes from your manuscript), or elaborate on this item by providing additional information not in the ms, or briefly explain why the item is not applicable/relevant for your study

"Lay-providers met the following criteria: (a) at least 18 years old, (b) completed secondary school in Kenya with English as the language of instruction, (c) able to read intervention protocols in English, and (d) available for all scheduled group sessions [19,22]. We recruited candidates openly through WhatsApp groups, university forums, and online job posting boards."

4a-i) Computer / Internet literacy

Computer / Internet literacy is often an implicit “de facto” eligibility criterion - this should be explicitly clarified.

	1	2	3	4	5	
subitem not at all important	○	○	○	◉	○	essential

Clear selection

Does your paper address subitem 4a-i?

Copy and paste relevant sections from the manuscript (include quotes in quotation marks "like this" to indicate direct quotes from your manuscript), or elaborate on this item by providing additional information not in the ms, or briefly explain why the item is not applicable/relevant for your study

Lay providers must be "able to read intervention protocols in English". While not explicitly stating computer/internet literacy as an eligibility criterion, the recruitment through "WhatsApp groups, university forums, and online job posting boards" implicitly suggests that participants would need a certain level of digital literacy. Hub coordinators also uploaded audio files, implying technical roles that would require such literacy.

4a-ii) Open vs. closed, web-based vs. face-to-face assessments:

Open vs. closed, web-based vs. face-to-face assessments: Mention how participants were recruited (online vs. offline), e.g., from an open access website or from a clinic, and clarify if this was a purely web-based trial, or there were face-to-face components (as part of the intervention or for assessment), i.e., to what degree got the study team to know the participant. In online-only trials, clarify if participants were quasi-anonymous and whether having multiple identities was possible or whether technical or logistical measures (e.g., cookies, email confirmation, phone calls) were used to detect/prevent these.

	1	2	3	4	5	
subitem not at all important	○	○	○	○	◉	essential

Clear selection

Does your paper address subitem 4a-ii? *

Copy and paste relevant sections from the manuscript (include quotes in quotation marks "like this" to indicate direct quotes from your manuscript), or elaborate on this item by providing additional information not in the ms, or briefly explain why the item is not applicable/relevant for your study

"The study was conducted at Shamiri Institute's Ngong Hub in Kajiado County, Kenya – one of Shamiri Institute's operational hubs serving schools within a 20-kilometer radius."

"Lay providers audio-recorded group sessions using digital voice recorders supplied by the study team. Recorders captured wide-angle audio in open or semi-open school spaces (e.g., classrooms, halls, outdoor areas), resulting in variable levels of background noise."

"Written informed consent was obtained from all adult lay providers and adult student participants prior to their involvement in the study. For minors, written assent was obtained from the student alongside parental or guardian consent, secured through school administration..."

4a-iii) Information giving during recruitment

Information given during recruitment. Specify how participants were briefed for recruitment and in the informed consent procedures (e.g., publish the informed consent documentation as appendix, see also item X26), as this information may have an effect on user self-selection, user expectation and may also bias results.

	1	2	3	4	5	
subitem not at all important	○	○	○	◉	○	essential

Clear selection

Does your paper address subitem 4a-iii?

Copy and paste relevant sections from the manuscript (include quotes in quotation marks "like this" to indicate direct quotes from your manuscript), or elaborate on this item by providing additional information not in the ms, or briefly explain why the item is not applicable/relevant for your study

"Written informed consent was obtained from all adult lay providers and adult student participants prior to their involvement in the study. For minors, written assent was obtained from the student alongside parental or guardian consent, secured through school administration, in accordance with DUISERC and NACOSTI protocols. Participants were informed of the voluntary nature of their involvement and their right to withdraw at any time without consequence."
"All 64 Shamiri Fellows assigned to Ngong Hub were eligible; sex and age data were collected via self-report." No specific information is given about whether participants were told about the AI-augmented supervision study during recruitment for their role as lay providers. However, for the fidelity validation procedure, "Lay providers were not directly exposed to the AI system; all AI-generated feedback was delivered to supervisors, who relayed it during supervision meetings." This suggests the lay providers were not informed about being specifically rated by an AI.

4b) Settings and locations where the data were collected

Does your paper address CONSORT subitem 4b? *

Copy and paste relevant sections from the manuscript (include quotes in quotation marks "like this" to indicate direct quotes from your manuscript), or elaborate on this item by providing additional information not in the ms, or briefly explain why the item is not applicable/relevant for your study

"Across six secondary schools in Ngong Hub, Kajiado County, Kenya (May-September 2025)..."
"The study was conducted at Shamiri Institute's Ngong Hub in Kajiado County, Kenya – one of Shamiri Institute's operational hubs serving schools within a 20-kilometer radius."
"Lay providers audio-recorded group sessions using digital voice recorders supplied by the study team. Recorders captured wide-angle audio in open or semi-open school spaces (e.g., classrooms, halls, outdoor areas)..."

4b-i) Report if outcomes were (self-)assessed through online questionnaires

Clearly report if outcomes were (self-)assessed through online questionnaires (as common in web-based trials) or otherwise.

	1	2	3	4	5	
subitem not at all important	○	○	○	○	◉	essential

Clear selection

Does your paper address subitem 4b-i? *

Copy and paste relevant sections from the manuscript (include quotes in quotation marks "like this" to indicate direct quotes from your manuscript), or elaborate on this item by providing additional information not in the ms, or briefly explain why the item is not applicable/relevant for your study

"shamiriAI generated automated scores across all six domains using its LLM-based inference pipeline (Stage 4). Separately, two independent human raters rated each session."

4b-ii) Report how institutional affiliations are displayed

Report how institutional affiliations are displayed to potential participants [on ehealth media], as affiliations with prestigious hospitals or universities may affect volunteer rates, use, and reactions with regards to an intervention.(Not a required item – describe only if this may bias results)

	1	2	3	4	5	
subitem not at all important	(•)	( )	( )	( )	( )	essential

Clear selection

Does your paper address subitem 4b-ii?

Copy and paste relevant sections from the manuscript (include quotes in quotation marks "like this" to indicate direct quotes from your manuscript), or elaborate on this item by providing additional information not in the ms, or briefly explain why the item is not applicable/relevant for your study

The paper does not explicitly report how institutional affiliations were displayed during participant recruitment. The recruitment was for lay providers for the Shamiri program, and students for the intervention, both within established school settings and through online job boards/WhatsApp groups, where institutional affiliations (Shamiri Institute) would likely be clear. This item is not directly applicable to the AI tool's validation itself.

5) The interventions for each group with sufficient details to allow replication, including how and when they were actually administered

5-i) Mention names, credential, affiliations of the developers, sponsors, and owners

Mention names, credential, affiliations of the developers, sponsors, and owners [6] (if authors/evaluators are owners or developer of the software, this needs to be declared in a “Conflict of interest” section or mentioned elsewhere in the manuscript).

	1	2	3	4	5	
subitem not at all important	( )	( )	( )	( )	(•)	essential

Clear selection

Does your paper address subitem 5-i?

Copy and paste relevant sections from the manuscript (include quotes in quotation marks "like this" to indicate direct quotes from your manuscript), or elaborate on this item by providing additional information not in the ms, or briefly explain why the item is not applicable/relevant for your study

"SL, BM, TO, WM, RK, FK, and RD are employees of Shamiri Institute, a non-profit organization based in the Republic of Kenya. CW is a member of the Board of Directors of Shamiri Institute. The authors declare no other competing interests."
They dentified with their affiliations (Shamiri Institute, Kenyatta University) and their roles as employees/board members of Shamiri Institute, which is the developer and owner of shamiriAI.

5-ii) Describe the history/development process

Describe the history/development process of the application and previous formative evaluations (e.g., focus groups, usability testing), as these will have an impact on adoption/use rates and help with interpreting results.

	1	2	3	4	5	
subitem not at all important	○	○	○	○	◉	essential

Clear selection

Does your paper address subitem 5-ii?

Copy and paste relevant sections from the manuscript (include quotes in quotation marks "like this" to indicate direct quotes from your manuscript), or elaborate on this item by providing additional information not in the ms, or briefly explain why the item is not applicable/relevant for your study

Does your paper address subitem 5-ii?
"The system prompt was developed iteratively across three versions using a corpus of group session recordings collected prior to the 2025 validation study. This development corpus comprised earlier Shamiri Hub session recordings that were distinct from and did not overlap with the 52 sessions used in the validation dataset. Prompt refinements across versions were driven by qualitative review of model outputs on this development corpus and by progressively closer alignment with the Shamiri Intervention Protocol rubric criteria. The final prompt (iteration_three) was locked prior to the assembly and scoring of the 52-session validation dataset."

5-iii) Revisions and updating

Revisions and updating. Clearly mention the date and/or version number of the application/intervention (and comparator, if applicable) evaluated, or describe whether the intervention underwent major changes during the evaluation process, or whether the development and/or content was “frozen” during the trial. Describe dynamic components such as news feeds or changing content which may have an impact on the replicability of the intervention (for unexpected events see item 3b).

	1	2	3	4	5	
subitem not at all important	○	○	○	○	◉	essential

Clear selection

Does your paper address subitem 5-iii?

Copy and paste relevant sections from the manuscript (include quotes in quotation marks "like this" to indicate direct quotes from your manuscript), or elaborate on this item by providing additional information not in the ms, or briefly explain why the item is not applicable/relevant for your study

"The final prompt (iteration_three) was locked prior to the assembly and scoring of the 52-session validation dataset. No validation sessions were reviewed, scored, or otherwise consulted during prompt development. The 52 sessions therefore constituted a clean held-out evaluation of the locked prompt, with no leakage between the prompt-development and validation phases."

5-iv) Quality assurance methods

Provide information on quality assurance methods to ensure accuracy and quality of information provided [1], if applicable.

	1	2	3	4	5	
subitem not at all important	○	○	○	○	◉	essential

Clear selection

Does your paper address subitem 5-iv?

Copy and paste relevant sections from the manuscript (include quotes in quotation marks "like this" to indicate direct quotes from your manuscript), or elaborate on this item by providing additional information not in the ms, or briefly explain why the item is not applicable/relevant for your study

"Quality assurance methods to ensure accuracy and quality of information provided:"
The quality of the ASR component was assessed through "Character Error Rate (CER), Word Error Rate (WER), and semantic similarity via cosine similarity between sentence embeddings from a multilingual model".
"The model was configured to return a strictly typed JSON object validated against a Pydantic response schema, which enforced integer scores on the 1-7 scale for each rubric dimension and prevented continuous or out-of-range values. No post-processing or regex extraction was applied to model outputs; scores were consumed directly from the validated JSON object."
"Supervisors were encouraged to interpret AI-generated ratings and feedback alongside their own direct knowledge of Lay providers and clinical judgment."

5-v) Ensure replicability by publishing the source code, and/or providing screenshots/screen-capture video, and/or providing flowcharts of the algorithms used

Ensure replicability by publishing the source code, and/or providing screenshots/screen-capture video, and/or providing flowcharts of the algorithms used. Replicability (i.e., other researchers should in principle be able to replicate the study) is a hallmark of scientific reporting.

	1	2	3	4	5	
subitem not at all important	○	○	○	○	◉	essential

Clear selection

Does your paper address subitem 5-v?

Copy and paste relevant sections from the manuscript (include quotes in quotation marks "like this" to indicate direct quotes from your manuscript), or elaborate on this item by providing additional information not in the ms, or briefly explain why the item is not applicable/relevant for your study

"Data and analysis code is available on Open Science Framework (DOI: 10.17605/OSF.IO/NU9TZ)."
"Table 1 provides an overview of each stage." (This table serves as a flowchart/description of the pipeline).
"The structured system prompt (reproduced verbatim in Supplement C9) provided..."
"The primary inputs to shamiriAI are audio recordings of group sessions, typically in .wav or .mp3 format..."

5-vi) Digital preservation

Digital preservation: Provide the URL of the application, but as the intervention is likely to change or disappear over the course of the years; also make sure the intervention is archived (Internet Archive, webcitation.org, and/or publishing the source code or screenshots/videos alongside the article). As pages behind login screens cannot be archived, consider creating demo pages which are accessible without login.

	1	2	3	4	5	
subitem not at all important	○	○	○	○	◉	essential

Clear selection

Does your paper address subitem 5-vi?

Copy and paste relevant sections from the manuscript (include quotes in quotation marks "like this" to indicate direct quotes from your manuscript), or elaborate on this item by providing additional information not in the ms, or briefly explain why the item is not applicable/relevant for your study

"Data and analysis code is available on Open Science Framework (DOI: 10.17605/OSF.IO/NU9TZ)."
"URL of your Intervention Website or App: https://huggingface.co/shamiri-ai/models"

5-vii) Access

Access: Describe how participants accessed the application, in what setting/context, if they had to pay (or were paid) or not, whether they had to be a member of specific group. If known, describe how participants obtained “access to the platform and Internet” [1]. To ensure access for editors/reviewers/readers, consider to provide a “backdoor” login account or demo mode for reviewers/readers to explore the application (also important for archiving purposes, see vi).

	1	2	3	4	5	
subitem not at all important	○	○	○	○	◉	essential

Clear selection

Does your paper address subitem 5-vii? *

Copy and paste relevant sections from the manuscript (include quotes in quotation marks "like this" to indicate direct quotes from your manuscript), or elaborate on this item by providing additional information not in the ms, or briefly explain why the item is not applicable/relevant for your study

"Lay providers audio-recorded group sessions using digital voice recorders supplied by the study team... For the AI-augmented supervision arm, hub coordinators uploaded audio files to secure cloud storage after each session... Reports were delivered to supervisors via secure email or shared folder and reviewed during weekly supervision meetings."
"Lay providers (Shamiri Fellows) were compensated at the standard Shamiri Institute rate of KES 1,500 (approximately USD 12) per one-hour session delivered, consistent with their employment terms. Student participants received no financial compensation."

5-viii) Mode of delivery, features/functionalities/components of the intervention and comparator, and the theoretical framework

Describe mode of delivery, features/functionalities/components of the intervention and comparator, and the theoretical framework [6] used to design them (instructional strategy [1], behaviour change techniques, persuasive features, etc., see e.g., [7, 8] for terminology). This includes an in-depth description of the content (including where it is coming from and who developed it) [1],” whether [and how] it is tailored to individual circumstances and allows users to track their progress and receive feedback” [6]. This also includes a description of communication delivery channels and – if computer-mediated communication is a component – whether communication was synchronous or asynchronous [6]. It also includes information on presentation strategies [1], including page design principles, average amount of text on pages, presence of hyperlinks to other resources, etc. [1].

	1	2	3	4	5	
subitem not at all important	○	○	○	○	◉	essential

Clear selection

Does your paper address subitem 5-viii? *

Copy and paste relevant sections from the manuscript (include quotes in quotation marks "like this" to indicate direct quotes from your manuscript), or elaborate on this item by providing additional information not in the ms, or briefly explain why the item is not applicable/relevant for your study

"shamiriAI is an AI-based tool designed to enhance fidelity monitoring and supervision of laydelivered group interventions. It processes raw session audio and produces structured feedback reports for clinical supervisors, covering both content-related and process-related aspects of delivery. The system operates in a code-switched multilingual environment—sessions occur in a mix of English, Kiswahili, and Sheng—characteristic of Kenyan secondary schools. shamiriAI follows a linear, five-stage pipeline: (1) ingestion and preprocessing, (2) core processing (ASR and prosodic feature extraction, running in parallel), (3) post-processing and personally identifiable information (PII) scrubbing, (4) large language model (LLM)-based feedback inference, and (5) delivery of feedback reports."
"The structured system prompt (reproduced verbatim in Supplement C9) provided: (a) background on the Shamiri intervention, its session-by-session structure, and supervision goals; (b) the six fidelity domains to be assessed (Required Contents, Specifics, Thoroughness, Clarity, Skill, and Purity), including scoring rubric and behavioral anchors; (c) the redacted transcript and a session-level audio features object; and (d) a request for structured feedback including numerical ratings per domain, specific strengths, areas requiring improvement with transcript evidence, and suggested focus areas for the upcoming supervision session."

5-ix) Describe use parameters

Describe use parameters (e.g., intended “doses” and optimal timing for use). Clarify what instructions or recommendations were given to the user, e.g., regarding timing, frequency, heaviness of use, if any, or was the intervention used ad libitum.

	1	2	3	4	5	
subitem not at all important	○	○	○	◉	○	essential

Clear selection

Does your paper address subitem 5-ix?

Copy and paste relevant sections from the manuscript (include quotes in quotation marks "like this" to indicate direct quotes from your manuscript), or elaborate on this item by providing additional information not in the ms, or briefly explain why the item is not applicable/relevant for your study

"shamiriAI processed session audio... across 52 recorded sessions"
"reports were delivered to supervisors via secure email or shared folder and reviewed during weekly supervision meetings."
This indicates that the AI tool is designed for weekly use, aligning with the "weekly group sessions" of the Shamiri intervention. No specific "instructions or recommendations" are given for the AI's use by supervisors, beyond interpreting feedback "alongside their own direct knowledge... and clinical judgment."

5-x) Clarify the level of human involvement

Clarify the level of human involvement (care providers or health professionals, also technical assistance) in the e-intervention or as co-intervention (detail number and expertise of professionals involved, if any, as well as “type of assistance offered, the timing and frequency of the support, how it is initiated, and the medium by which the assistance is delivered”. It may be necessary to distinguish between the level of human involvement required for the trial, and the level of human involvement required for a routine application outside of a RCT setting (discuss under item 21 – generalizability).

	1	2	3	4	5	
subitem not at all important	○	○	○	○	◉	essential

Clear selection

Does your paper address subitem 5-x?

Copy and paste relevant sections from the manuscript (include quotes in quotation marks "like this" to indicate direct quotes from your manuscript), or elaborate on this item by providing additional information not in the ms, or briefly explain why the item is not applicable/relevant for your study

"shamiriAI is designed as a clinician-facing decision-support tool: supervisors remain the primary decisionmakers, and AI-generated feedback is treated as one structured input alongside direct clinical knowledge."
"In the shamiriAI arm, supervisors continued all aspects of standard supervision but additionally received structured feedback reports generated by the shamiriAI system for recorded sessions. Supervisors reviewed these reports before or during supervision and used them to guide discussions with lay providers. Lay providers were not directly exposed to the AI system; all AI-generated feedback was delivered to supervisors, who relayed it during supervision meetings."

5-xi) Report any prompts/reminders used

Report any prompts/reminders used: Clarify if there were prompts (letters, emails, phone calls, SMS) to use the application, what triggered them, frequency etc. It may be necessary to distinguish between the level of prompts/reminders required for the trial, and the level of prompts/reminders for a routine application outside of a RCT setting (discuss under item 21 – generalizability).

	1	2	3	4	5	
subitem not at all important	○	○	●	○	○	essential

Clear selection

Does your paper address subitem 5-xi? *

Copy and paste relevant sections from the manuscript (include quotes in quotation marks "like this" to indicate direct quotes from your manuscript), or elaborate on this item by providing additional information not in the ms, or briefly explain why the item is not applicable/relevant for your study

The paper describes prompts used within the AI model for generating feedback (the "structured system prompt" for the LLM), but not prompts or reminders to users (supervisors or lay providers) to use the shamiriAI system. The delivery of reports to supervisors was via "secure email or shared folder," which could implicitly act as a reminder, but this is not explicitly stated as a "prompt."

5-xii) Describe any co-interventions (incl. training/support)

Describe any co-interventions (incl. training/support): Clearly state any interventions that are provided in addition to the targeted eHealth intervention, as ehealth intervention may not be designed as stand-alone intervention. This includes training sessions and support [1]. It may be necessary to distinguish between the level of training required for the trial, and the level of training for a routine application outside of a RCT setting (discuss under item 21 – generalizability.

	1	2	3	4	5	
subitem not at all important	○	○	○	○	●	essential

Clear selection

Does your paper address subitem 5-xii? *

Copy and paste relevant sections from the manuscript (include quotes in quotation marks "like this" to indicate direct quotes from your manuscript), or elaborate on this item by providing additional information not in the ms, or briefly explain why the item is not applicable/relevant for your study

"In the shamiriAI arm, supervisors continued all aspects of standard supervision but additionally received structured feedback reports generated by the shamiriAI system for recorded sessions. Supervisors reviewed these reports before or during supervision and used them to guide discussions with lay providers."

6a) Completely defined pre-specified primary and secondary outcome measures, including how and when they were assessed

Does your paper address CONSORT subitem 6a? *

Copy and paste relevant sections from the manuscript (include quotes in quotation marks "like this" to indicate direct quotes from your manuscript), or elaborate on this item by providing additional information not in the ms, or briefly explain why the item is not applicable/relevant for your study

"We pursued two pilot aims. The first was to evaluate the performance of shamiriAI's multilingual ASR pipeline on a held-out test set of manually transcribed sessions... The second was to assess interrater reliability between shamiriAI-generated fidelity ratings and independent human supervisor ratings across six fidelity dimensions..."
"Intervention fidelity, used during supervision, was rated using a structured six-domain instrument developed and used in prior trials of Shamiri intervention and for routine quality monitoring. The instrument covers the following domains; each rated on a 1–7 scale (1 = poor; 7 = excellent): Required Contents, Specifics, Thoroughness, Clarity, Skill, Purity."
"Transcription Performance...evaluated ASR performance on the held-out test set using the multi-metric approach... (CER, WER, semantic similarity)."
Elaboration: The primary outcomes (ASR performance and interrater reliability of fidelity ratings across six domains) are clearly defined, along with the 1-7 rating scale for fidelity and the specific metrics for ASR. They were assessed by comparing AI outputs to manually transcribed sessions (for ASR) and human supervisor ratings (for fidelity).

6a-i) Online questionnaires: describe if they were validated for online use and apply CHERRIES items to describe how the questionnaires were designed/deployed

If outcomes were obtained through online questionnaires, describe if they were validated for online use and apply CHERRIES items to describe how the questionnaires were designed/deployed [9].

	1	2	3	4	5	
subitem not at all important	○	○	◉	○	○	essential

Clear selection

Does your paper address subitem 6a-i?

Copy and paste relevant sections from manuscript text

The paper does not explicitly state that human supervisors used online questionnaires for their fidelity ratings, nor does it discuss their validation for online use or apply CHERRIES items. It mentions that "All lay providers were evaluated using a standardized rubric assessing content accuracy, timing, and subjective quality" during training, and supervisors were trained on "fidelity rating using the six-domain instrument". The AI itself generated structured JSON output, which was then validated against a Pydantic response schema.

6a-ii) Describe whether and how "use" (including intensity of use/dosage) was defined/measured/monitored

Describe whether and how "use" (including intensity of use/dosage) was defined/measured/monitored (logins, logfile analysis, etc.). Use/adoption metrics are important process outcomes that should be reported in any ehealth trial.

	1	2	3	4	5	
subitem not at all important	○	○	○	○	◉	essential

Clear selection

Does your paper address subitem 6a-ii?

Copy and paste relevant sections from manuscript text

"For the present analyses, we included recordings from all 52 delivered sessions for which recordings were available (100% of recorded sessions). For the AI-augmented supervision arm, hub coordinators uploaded audio files to secure cloud storage after each session."

6a-iii) Describe whether, how, and when qualitative feedback from participants was obtained

Describe whether, how, and when qualitative feedback from participants was obtained (e.g., through emails, feedback forms, interviews, focus groups).

	1	2	3	4	5	
subitem not at all important	(•)	○	○	○	○	essential

Clear selection

Does your paper address subitem 6a-iii?

Copy and paste relevant sections from manuscript text

Mentions that for each processed session, shamiriAI generated a PDF report containing a "narrative feedback section structured around observed strengths, areas for improvement, and suggested focus areas." This is AI-generated qualitative feedback. It does not explicitly state that qualitative feedback was obtained from participants (lay providers or supervisors) for the purpose of this pilot validation.

6b) Any changes to trial outcomes after the trial commenced, with reasons

Does your paper address CONSORT subitem 6b? *

Copy and paste relevant sections from the manuscript (include quotes in quotation marks "like this" to indicate direct quotes from your manuscript), or elaborate on this item by providing additional information not in the ms, or briefly explain why the item is not applicable/relevant for your study

"No validation sessions were reviewed, scored, or otherwise consulted during prompt development. The 52 sessions therefore constituted a clean held-out evaluation of the locked prompt, with no leakage between the prompt-development and validation phases." Elaboration: The manuscript indicates that outcomes were pre-specified and no changes were made after the trial commencement, as the system was "locked" for the validation.

7a) How sample size was determined

NPT: When applicable, details of whether and how the clustering by care provides or centers was addressed

7a-i) Describe whether and how expected attrition was taken into account when calculating the sample size

Describe whether and how expected attrition was taken into account when calculating the sample size.

	1	2	3	4	5	
subitem not at all important	○	○	◉	○	○	essential

Clear selection

Does your paper address subitem 7a-i?

Copy and paste relevant sections from manuscript title (include quotes in quotation marks "like this" to indicate direct quotes from your manuscript), or elaborate on this item by providing additional information not in the ms, or briefly explain why the item is not applicable/relevant for your study

The study is a pilot validation of a tool's performance on a fixed set of recorded sessions, not a clinical trial with participant attrition affecting sample size for outcomes. The sample size of 52 sessions was based on available data for validation, not a power calculation factoring in attrition.

7b) When applicable, explanation of any interim analyses and stopping guidelines

Does your paper address CONSORT subitem 7b? *

Copy and paste relevant sections from the manuscript (include quotes in quotation marks "like this" to indicate direct quotes from your manuscript), or elaborate on this item by providing additional information not in the ms, or briefly explain why the item is not applicable/relevant for your study

"Not applicable."

Elaboration: As a pilot validation study of an AI tool's technical performance and interrater reliability, no interim analyses or stopping guidelines were applicable. The data collection for this validation was a fixed set of 52 sessions.

8a) Method used to generate the random allocation sequence

NPT: When applicable, how care providers were allocated to each trial group

Does your paper address CONSORT subitem 8a? *

Copy and paste relevant sections from the manuscript (include quotes in quotation marks "like this" to indicate direct quotes from your manuscript), or elaborate on this item by providing additional information not in the ms, or briefly explain why the item is not applicable/relevant for your study

"The 47 fellows who participated in the parallel A/B test were randomized at project start to AI-augmented (n=34) or standard supervision (n=13) arms."
"Audio recording for fidelity validation was a separate procedure: at each school session, the Hub Coordinator wrote the names of all lay providers present on slips of paper and drew three without replacement for recording, with no stratification by supervision arm. This produced a validation set of 52 recorded sessions distributed unevenly across arms (38 AI-augmented; 14 standard) and across providers, reflecting natural variation in attendance and recording opportunities at study time rather than a pre-specified arm-balanced design."

For the fidelity validation, session selection for recording was done by drawing names from slips of paper by the Hub Coordinator, which is a method for random selection.

8b) Type of randomisation; details of any restriction (such as blocking and block size)

Does your paper address CONSORT subitem 8b? *

Copy and paste relevant sections from the manuscript (include quotes in quotation marks "like this" to indicate direct quotes from your manuscript), or elaborate on this item by providing additional information not in the ms, or briefly explain why the item is not applicable/relevant for your study

"Audio recording for fidelity validation was a separate procedure: at each school session, the Hub Coordinator wrote the names of all lay providers present on slips of paper and drew three without replacement for recording, with no stratification by supervision arm."

9) Mechanism used to implement the random allocation sequence (such as sequentially numbered containers), describing any steps taken to conceal the sequence until interventions were assigned

Does your paper address CONSORT subitem 9? *

Copy and paste relevant sections from the manuscript (include quotes in quotation marks "like this" to indicate direct quotes from your manuscript), or elaborate on this item by providing additional information not in the ms, or briefly explain why the item is not applicable/relevant for your study

"At each school session, the Hub Coordinator wrote the names of all lay providers present on slips of paper and drew three without replacement for recording"

The mechanism for selecting sessions for recording is described as drawing names from slips of paper. For the randomization of lay providers to intervention arms in the broader study, details are not provided in this manuscript.

10) Who generated the random allocation sequence, who enrolled participants, and who assigned participants to interventions

Does your paper address CONSORT subitem 10? *

Copy and paste relevant sections from the manuscript (include quotes in quotation marks "like this" to indicate direct quotes from your manuscript), or elaborate on this item by providing additional information not in the ms, or briefly explain why the item is not applicable/relevant for your study

"The Hub Coordinator wrote the names of all lay providers present on slips of paper and drew three without replacement for recording"

11a) If done, who was blinded after assignment to interventions (for example, participants, care providers, those assessing outcomes) and how

NPT: Whether or not administering co-interventions were blinded to group assignment

11a-i) Specify who was blinded, and who wasn’t

Specify who was blinded, and who wasn’t. Usually, in web-based trials it is not possible to blind the participants [1, 3] (this should be clearly acknowledged), but it may be possible to blind outcome assessors, those doing data analysis or those administering co-interventions (if any).

	1	2	3	4	5	
subitem not at all important	○	○	○	○	●	essential

Clear selection

Does your paper address subitem 11a-i? *

Copy and paste relevant sections from the manuscript (include quotes in quotation marks "like this" to indicate direct quotes from your manuscript), or elaborate on this item by providing additional information not in the ms, or briefly explain why the item is not applicable/relevant for your study

"Lay providers were not directly exposed to the AI system; all AI-generated feedback was delivered to supervisors, who relayed it during supervision meetings."
"Human raters were blind to AI-generated scores and to each other's ratings."

11a-ii) Discuss e.g., whether participants knew which intervention was the "intervention of interest" and which one was the "comparator"

Informed consent procedures (4a-ii) can create biases and certain expectations - discuss e.g., whether participants knew which intervention was the "intervention of interest" and which one was the "comparator".

	1	2	3	4	5	
subitem not at all important	○	○	○	◉	○	essential

Clear selection

Does your paper address subitem 11a-ii?

Copy and paste relevant sections from the manuscript (include quotes in quotation marks "like this" to indicate direct quotes from your manuscript), or elaborate on this item by providing additional information not in the ms, or briefly explain why the item is not applicable/relevant for your study

"Lay providers were not directly exposed to the AI system; all AI-generated feedback was delivered to supervisors, who relayed it during supervision meetings." This implies that the lay providers were not explicitly told about shamiriAI as an "intervention of interest" for their own practice.

11b) If relevant, description of the similarity of interventions

(this item is usually not relevant for ehealth trials as it refers to similarity of a placebo or sham intervention to a active medication/intervention)

Does your paper address CONSORT subitem 11b? *

Copy and paste relevant sections from the manuscript (include quotes in quotation marks "like this" to indicate direct quotes from your manuscript), or elaborate on this item by providing additional information not in the ms, or briefly explain why the item is not applicable/relevant for your study

"Not applicable."
Elaboration: This item is not relevant as the study is a pilot validation of an AI tool for fidelity monitoring, not a comparison of a placebo or sham intervention to an active medical intervention. The "comparator" for shamiriAI's output was human expert ratings, not another intervention.

12a) Statistical methods used to compare groups for primary and secondary outcomes

NPT: When applicable, details of whether and how the clustering by care providers or centers was addressed

Does your paper address CONSORT subitem 12a? *

Copy and paste relevant sections from the manuscript (include quotes in quotation marks "like this" to indicate direct quotes from your manuscript), or elaborate on this item by providing additional information not in the ms, or briefly explain why the item is not applicable/relevant for your study

"Primary reliability analyses compared AI ratings against the human composite. We computed the following indices for each of the six fidelity dimensions: Intraclass Correlation Coefficient (ICC)... Agreement Categories... Bland-Altman Analysis... Dimension-level Bias... paired-sample t-tests (two-tailed, α = .05) comparing AI against the human composite on each dimension and on Average Fidelity... Holm-Bonferroni correction across these seven simultaneous comparisons... Cohen's d effect sizes."
"Secondary Analysis: Gwet's AC2 with ordinal weights... (1) AI vs each individual human rater... (2) AI vs rounded human composite..."

12a-i) Imputation techniques to deal with attrition / missing values

Imputation techniques to deal with attrition / missing values: Not all participants will use the intervention/comparator as intended and attrition is typically high in ehealth trials. Specify how participants who did not use the application or dropped out from the trial were treated in the statistical analysis (a complete case analysis is strongly discouraged, and simple imputation techniques such as LOCF may also be problematic [4]).

	1	2	3	4	5	
subitem not at all important	○	○	○	○	◉	essential

Clear selection

Does your paper address subitem 12a-i? *

Copy and paste relevant sections from the manuscript (include quotes in quotation marks "like this" to indicate direct quotes from your manuscript), or elaborate on this item by providing additional information not in the ms, or briefly explain why the item is not applicable/relevant for your study

"There were no missing ratings: all 52 sessions had complete AI and human scores on all six fidelity dimensions."
No imputation techniques were needed as there were no missing data for the fidelity ratings in the 52 sessions analyzed.

12b) Methods for additional analyses, such as subgroup analyses and adjusted analyses

Does your paper address CONSORT subitem 12b? *

Copy and paste relevant sections from the manuscript (include quotes in quotation marks "like this" to indicate direct quotes from your manuscript), or elaborate on this item by providing additional information not in the ms, or briefly explain why the item is not applicable/relevant for your study

"Demographic Subgroup Analyses: To examine whether AI–human bias varied by lay-provider demographics, we stratified the per session AI minus human composite difference by lay-provider sex (Female / Male) and fellow age band (median split at 20 years). Welch's two-sample t-tests on the AI–human difference were conducted separately for each fidelity dimension; Gwet's AC2 (ordinal weights, against the rounded human composite) was computed within each stratum..."
"Per-Arm Robustness Check: ...compared AI rating means, human composite means, and per-arm Gwet's AC2 between the two arms for each of the six fidelity dimensions. Welch's two-sample t-tests assessed between-arm differences..."

X26) REB/IRB Approval and Ethical Considerations [recommended as subheading under "Methods"] (not a CONSORT item)

X26-i) Comment on ethics committee approval

	1	2	3	4	5	
subitem not at all important	○	○	○	○	●	essential

Clear selection

Does your paper address subitem X26-i?

Copy and paste relevant sections from the manuscript (include quotes in quotation marks "like this" to indicate direct quotes from your manuscript), or elaborate on this item by providing additional information not in the ms, or briefly explain why the item is not applicable/relevant for your study

"The study was approved by the Daystar University Institutional Scientific and Ethics Review Committee (DUISERC; approval number: DU-ISERC/10/06/2025/000015E; approval date: 10 June 2025) and licensed by the National Commission for Science, Technology and Innovation (NACOSTI; license number: NACOSTI/P/25/415077). The trial was registered with the Pan African Clinical Trials Registry on 1 August 2025 (PACTR202508900479778)."

x26-ii) Outline informed consent procedures

Outline informed consent procedures e.g., if consent was obtained offline or online (how? Checkbox, etc.?), and what information was provided (see 4a-ii). See [6] for some items to be included in informed consent documents.

	1	2	3	4	5	
subitem not at all important	○	○	○	○	●	essential

Clear selection

Does your paper address subitem X26-ii?

Copy and paste relevant sections from the manuscript (include quotes in quotation marks "like this" to indicate direct quotes from your manuscript), or elaborate on this item by providing additional information not in the ms, or briefly explain why the item is not applicable/relevant for your study

"Written informed consent was obtained from all adult lay providers and adult student participants prior to their involvement in the study. For minors, written assent was obtained from the student alongside parental or guardian consent, secured through school administration, in accordance with DUISERC and NACOSTI protocols. Participants were informed of the voluntary nature of their involvement and their right to withdraw at any time without consequence."

X26-iii) Safety and security procedures

Safety and security procedures, incl. privacy considerations, and any steps taken to reduce the likelihood or detection of harm (e.g., education and training, availability of a hotline)

	1	2	3	4	5	
subitem not at all important	○	○	○	○	●	essential

Clear selection

Does your paper address subitem X26-iii?

Copy and paste relevant sections from the manuscript (include quotes in quotation marks "like this" to indicate direct quotes from your manuscript), or elaborate on this item by providing additional information not in the ms, or briefly explain why the item is not applicable/relevant for your study

"Privacy and confidentiality. Audio recordings and unredacted transcripts were stored on secure servers with restricted access. Prior to any AI inference, personally identifiable information including names, school identifiers, and location references was detected and masked using a named entity recognition pipeline and replaced with typed placeholders ([REDACTED_PERSON], [REDACTED_LOCATION], [REDACTED_ORGANIZATION]). Only de-identified, redacted transcripts were used in LLM-based fidelity inference. Further details on PII handling are described in the shamiriAI pipeline section and Supplement C7."

RESULTS

13a) For each group, the numbers of participants who were randomly assigned, received intended treatment, and were analysed for the primary outcome
NPT: The number of care providers or centers performing the intervention in each group and the number of patients treated by each care provider in each center

Does your paper address CONSORT subitem 13a? *

Copy and paste relevant sections from the manuscript (include quotes in quotation marks "like this" to indicate direct quotes from your manuscript), or elaborate on this item by providing additional information not in the ms, or briefly explain why the item is not applicable/relevant for your study

"Of the 64 Shamiri Fellows assigned to Ngong Hub, 47 participated in the study. Thirty-four were randomized to the AI-augmented supervision arm and 13 to standard supervision (see Supplement D1 for participant flow). ... Across fellows, 52 recorded sessions were included in the fidelity analysis. Most fellows contributed one or two recordings, and most rated sessions were from the AI-augmented supervision arm (38/52, 73%)."

13b) For each group, losses and exclusions after randomisation, together with reasons

Does your paper address CONSORT subitem 13b? (NOTE: Preferably, this is shown in a CONSORT flow diagram) *

Copy and paste relevant sections from the manuscript (include quotes in quotation marks "like this" to indicate direct quotes from your manuscript), or elaborate on this item by providing additional information not in the ms, or briefly explain why the item is not applicable/relevant for your study

"Of the 64 Shamiri Fellows assigned to Ngong Hub, 47 participated in the study."
"Recording coverage varied across sessions due to logistical and contextual factors, including device failures, environmental noise, and school-specific restrictions. For the present analyses, we included recordings from all 52 delivered sessions for which recordings were available (100% of recorded sessions)."
A CONSORT flow diagram is referenced in Supplement D1.

13b-i) Attrition diagram

Strongly recommended: An attrition diagram (e.g., proportion of participants still logging in or using the intervention/comparator in each group plotted over time, similar to a survival curve) or other figures or tables demonstrating usage/dose/engagement.

	1	2	3	4	5	
subitem not at all important	○	○	○	○	◉	essential

Clear selection

Does your paper address subitem 13b-i?

Copy and paste relevant sections from the manuscript or cite the figure number if applicable (include quotes in quotation marks "like this" to indicate direct quotes from your manuscript), or elaborate on this item by providing additional information not in the ms, or briefly explain why the item is not applicable/relevant for your study

References to "Supplement D1 for participant flow," are made, which is where such a diagram would typically be found in a full CONSORT report. The analysis uses 100% of available recordings (52 sessions), so attrition of sessions processed is not a factor for the results presented.

14a) Dates defining the periods of recruitment and follow-up

Does your paper address CONSORT subitem 14a? *

Copy and paste relevant sections from the manuscript (include quotes in quotation marks "like this" to indicate direct quotes from your manuscript), or elaborate on this item by providing additional information not in the ms, or briefly explain why the item is not applicable/relevant for your study

"Across six secondary schools in Ngong Hub, Kajiado County, Kenya (May-September 2025)..."
"Audio recordings of Shamiri sessions at Ngong Hub began in May 2025..."

14a-i) Indicate if critical “secular events” fell into the study period

Indicate if critical “secular events” fell into the study period, e.g., significant changes in Internet resources available or “changes in computer hardware or Internet delivery resources”

	1	2	3	4	5	
subitem not at all important	○	○	●	○	○	essential

Clear selection

Does your paper address subitem 14a-i?

Copy and paste relevant sections from the manuscript (include quotes in quotation marks "like this" to indicate direct quotes from your manuscript), or elaborate on this item by providing additional information not in the ms, or briefly explain why the item is not applicable/relevant for your study

No mention of any critical "secular events" or changes in Internet/computer resources that might have impacted the study during the May-September 2025 period because non occured.

14b) Why the trial ended or was stopped (early)

Does your paper address CONSORT subitem 14b? *

Copy and paste relevant sections from the manuscript (include quotes in quotation marks "like this" to indicate direct quotes from your manuscript), or elaborate on this item by providing additional information not in the ms, or briefly explain why the item is not applicable/relevant for your study

Not applicable. The pilot validation study was completed as planned within the specified timeframe (May-September 2025) and was not stopped early.

15) A table showing baseline demographic and clinical characteristics for each group

NPT: When applicable, a description of care providers (case volume, qualification, expertise, etc.) and centers (volume) in each group

Does your paper address CONSORT subitem 15? *

Copy and paste relevant sections from the manuscript (include quotes in quotation marks "like this" to indicate direct quotes from your manuscript), or elaborate on this item by providing additional information not in the ms, or briefly explain why the item is not applicable/relevant for your study

"Most were female (36/47, 76.6%). Fellows ranged in age from 18 to 23 years (M = 19.83, SD = 1.65). Across fellows, 52 recorded sessions were included in the fidelity analysis. Most fellows contributed one or two recordings, and most rated sessions were from the AI-augmented supervision arm (38/52, 73%). Seven supervisors participated: most were female (5/7, 71%), ranging in age from 23 to 30 years (M = 25.57, SD = 2.23). Five supervisors (71%) were assigned to the AI-augmented arm and two (29%) to the supervision-asusual arm. Sample characteristics are reported in Supplement D2."

15-i) Report demographics associated with digital divide issues

In ehealth trials it is particularly important to report demographics associated with digital divide issues, such as age, education, gender, social-economic status, computer/Internet/ehealth literacy of the participants, if known.

	1	2	3	4	5	
subitem not at all important	○	○	○	○	●	essential

Clear selection

Does your paper address subitem 15-i? *

Copy and paste relevant sections from the manuscript (include quotes in quotation marks "like this" to indicate direct quotes from your manuscript), or elaborate on this item by providing additional information not in the ms, or briefly explain why the item is not applicable/relevant for your study

"Most were female (36/47, 76.6%). Fellows ranged in age from 18 to 23 years (M = 19.83, SD = 1.65)."
"Lay-providers met the following criteria: (a) at least 18 years old, (b) completed secondary school in Kenya with English as the language of instruction..."
Reports on age and gender for lay providers and are made with notes on the requirement for secondary school completion with English as the language of instruction, which are relevant demographics for understanding the context of technology use in Kenya. Implicitly assumes a degree of digital literacy due to recruitment methods and the nature of the AI tool.

16) For each group, number of participants (denominator) included in each analysis and whether the analysis was by original assigned groups

16-i) Report multiple “denominators” and provide definitions

Report multiple “denominators” and provide definitions: Report N’s (and effect sizes) “across a range of study participation [and use] thresholds” [1], e.g., N exposed, N consented, N used more than x times, N used more than y weeks, N participants “used” the intervention/comparator at specific pre-defined time points of interest (in absolute and relative numbers per group). Always clearly define “use” of the intervention.

	1	2	3	4	5	
subitem not at all important	○	○	○	○	◉	essential

Clear selection

Does your paper address subitem 16-i? *

Copy and paste relevant sections from the manuscript (include quotes in quotation marks "like this" to indicate direct quotes from your manuscript), or elaborate on this item by providing additional information not in the ms, or briefly explain why the item is not applicable/relevant for your study

"The 52 sessions used for fidelity validation comprised 19 Session 1 (Growth Mindset I), 15 Session 2 (Growth Mindset II), 12 Session 3 (Gratitude), and 6 Session 4 (Values Affirmation) recordings."
"The primary data source for ASR evaluation was a held-out test set of 10 sessions with manually prepared reference transcripts."

16-ii) Primary analysis should be intent-to-treat

Primary analysis should be intent-to-treat, secondary analyses could include comparing only “users”, with the appropriate caveats that this is no longer a randomized sample (see 18-i).

	1	2	3	4	5	
subitem not at all important	○	○	◉	○	○	essential

Clear selection

Does your paper address subitem 16-ii?

Copy and paste relevant sections from the manuscript (include quotes in quotation marks "like this" to indicate direct quotes from your manuscript), or elaborate on this item by providing additional information not in the ms, or briefly explain why the item is not applicable/relevant for your study

The study is a pilot validation of a tool, not an effectiveness trial with intervention and control groups. Therefore, the concept of "intent-to-treat" analysis in the traditional sense is not applicable. The analyses compare AI ratings to human ratings on all available sessions for which the AI produced an output.

17a) For each primary and secondary outcome, results for each group, and the estimated effect size and its precision (such as 95% confidence interval)

Does your paper address CONSORT subitem 17a? *

Copy and paste relevant sections from the manuscript (include quotes in quotation marks "like this" to indicate direct quotes from your manuscript), or elaborate on this item by providing additional information not in the ms, or briefly explain why the item is not applicable/relevant for your study

"The ASR model achieved Character Error Rate 0.19, Word Error Rate 0.34, and cosine semantic similarity 0.77..."
"AI fidelity scores were systematically lower than the human composite overall ($M = 5.14$ vs 5.93; $\Delta = -0.79$, $d = -1.16$, $p < .001$). The primary AI–human agreement analysis returned ICCs ranging from -0.06 to 0.20 across the six domains; sensitivity analyses using Gwet's AC2 against each individual human rater and against the rounded human composite (ordinal weights) corroborated the ICC-derived dimension-level ordering."

17a-i) Presentation of process outcomes such as metrics of use and intensity of use

In addition to primary/secondary (clinical) outcomes, the presentation of process outcomes such as metrics of use and intensity of use (dose, exposure) and their operational definitions is critical. This does not only refer to metrics of attrition (13-b) (often a binary variable), but also to more continuous exposure metrics such as “average session length”. These must be accompanied by a technical description how a metric like a “session” is defined (e.g., timeout after idle time) [1] (report under item 6a).

	1	2	3	4	5	
subitem not at all important	○	○	○	○	◉	essential

Clear selection

Does your paper address subitem 17a-i?

Copy and paste relevant sections from the manuscript (include quotes in quotation marks "like this" to indicate direct quotes from your manuscript), or elaborate on this item by providing additional information not in the ms, or briefly explain why the item is not applicable/relevant for your study

"We included recordings from all 52 delivered sessions for which recordings were available (100% of recorded sessions)."

17b) For binary outcomes, presentation of both absolute and relative effect sizes is recommended

Does your paper address CONSORT subitem 17b? *

Copy and paste relevant sections from the manuscript (include quotes in quotation marks "like this" to indicate direct quotes from your manuscript), or elaborate on this item by providing additional information not in the ms, or briefly explain why the item is not applicable/relevant for your study

"The AI underrated Required Contents (M_1 = 3.23, SD = 0.92 vs. M2 = 6.14, SD = 0.74; ΔM = -2.91, 95% CI [-3.25, -2.58], t(51) = -17.58, p < .001, d= -3.48)."
For continuous outcomes (fidelity ratings), mean differences and Cohen's d effect sizes are reported with 95% CIs. For agreement categories, percentages (absolute) are given.

18) Results of any other analyses performed, including subgroup analyses and adjusted analyses, distinguishing pre-specified from exploratory

Does your paper address CONSORT subitem 18? *

Copy and paste relevant sections from the manuscript (include quotes in quotation marks "like this" to indicate direct quotes from your manuscript), or elaborate on this item by providing additional information not in the ms, or briefly explain why the item is not applicable/relevant for your study

"Demographic Subgroup Analyses: These subgroup tests were exploratory and not multiplicitycorrected; they are reported descriptively rather than as confirmatory hypothesis tests."
"Per-Arm Robustness Check: As with the demographic subgroup analyses, these tests are exploratory and reported descriptively."

18-i) Subgroup analysis of comparing only users

A subgroup analysis of comparing only users is not uncommon in ehealth trials, but if done, it must be stressed that this is a self-selected sample and no longer an unbiased sample from a randomized trial (see 16-iii).

	1	2	3	4	5	
subitem not at all important	○	○	○	○	●	essential

Clear selection

Does your paper address subitem 18-i?

Copy and paste relevant sections from the manuscript (include quotes in quotation marks "like this" to indicate direct quotes from your manuscript), or elaborate on this item by providing additional information not in the ms, or briefly explain why the item is not applicable/relevant for your study

We conduct subgroup analyses (by lay-provider sex and age band) and per-arm robustness checks comparing AI and human ratings, but it does not specifically compare "only users" in the sense of a self-selected group within a clinical trial. The users of the AI system are supervisors, and the AI itself is the object of validation.

19) All important harms or unintended effects in each group
(for specific guidance see CONSORT for harms)

Does your paper address CONSORT subitem 19? *

Copy and paste relevant sections from the manuscript (include quotes in quotation marks "like this" to indicate direct quotes from your manuscript), or elaborate on this item by providing additional information not in the ms, or briefly explain why the item is not applicable/relevant for your study

"No statistically significant bias was observed for Specifics (ΔM = -0.28, padj = .176, d = -0.34) or Purity (ΔM = +0.23, padj = .220, d = +0.22); these two dimensions also showed the highest adjacent agreement rates (78.8% and 73.1%, respectively)."
"The AI underrated Required Contents (M_1 = 3.23, SD = 0.92 vs. M2 = 6.14, SD = 0.74; ΔM = -2.91, 95% CI [-3.25, -2.58], t(51) = -17.58, p < .001, d= -3.48)."
Systematic biases of the AI on certain fidelity dimensions are discussed as "limitations" and "diagnosable misalignments" in performance, which could be interpreted as "unintended effects" in terms of measurement accuracy. No direct "harms" to participants are reported from the use of the AI system itself.

19-i) Include privacy breaches, technical problems

Include privacy breaches, technical problems. This does not only include physical "harm" to participants, but also incidents such as perceived or real privacy breaches [1], technical problems, and other unexpected/unintended incidents. "Unintended effects" also includes unintended positive effects [2].

	1	2	3	4	5	
subitem not at all important	○	○	○	○	◉	essential

Clear selection

Does your paper address subitem 19-i?

Copy and paste relevant sections from the manuscript (include quotes in quotation marks "like this" to indicate direct quotes from your manuscript), or elaborate on this item by providing additional information not in the ms, or briefly explain why the item is not applicable/relevant for your study

"Privacy and confidentiality. Audio recordings and unredacted transcripts were stored on secure servers with restricted access. Prior to any AI inference, personally identifiable information including names, school identifiers, and location references was detected and masked using a named entity recognition pipeline and replaced with typed placeholders... Only de-identified, redacted transcripts were used in LLM-based fidelity inference."

19-ii) Include qualitative feedback from participants or observations from staff/researchers

Include qualitative feedback from participants or observations from staff/researchers, if available, on strengths and shortcomings of the application, especially if they point to unintended/unexpected effects or uses. This includes (if available) reasons for why people did or did not use the application as intended by the developers.

	1	2	3	4	5	
subitem not at all important	○	◉	○	○	○	essential

Clear selection

Does your paper address subitem 19-ii?

Copy and paste relevant sections from the manuscript (include quotes in quotation marks "like this" to indicate direct quotes from your manuscript), or elaborate on this item by providing additional information not in the ms, or briefly explain why the item is not applicable/relevant for your study

We discuss the AI's narrative feedback reports but do not report qualitative feedback from participants or staff/researchers about the AI system's strengths or shortcomings.

DISCUSSION

22) Interpretation consistent with results, balancing benefits and harms, and considering other relevant evidence

NPT: In addition, take into account the choice of the comparator, lack of or partial blinding, and unequal expertise of care providers or centers in each group

22-i) Restate study questions and summarize the answers suggested by the data, starting with primary outcomes and process outcomes (use)

Restate study questions and summarize the answers suggested by the data, starting with primary outcomes and process outcomes (use).

	1	2	3	4	5	
subitem not at all important	○	○	○	○	◉	essential

Clear selection

Does your paper address subitem 22-i? *

Copy and paste relevant sections from the manuscript (include quotes in quotation marks "like this" to indicate direct quotes from your manuscript), or elaborate on this item by providing additional information not in the ms, or briefly explain why the item is not applicable/relevant for your study

"This pilot study pursued two sequential foundational aims for shamiriAI — an automated fidelity monitoring system for lay-delivered group mental health interventions in multilingual, lowresource settings. The first aim established that the fine-tuned Whisper pipeline produces transcripts of sufficient semantic quality for downstream fidelity evaluation... The second aim produced a more complex picture. Across all six fidelity dimensions, shamiriAI rated sessions an average of 0.79 points lower than the human expert panel..."
The discussion begins by restating the two main aims and then summarizes the findings regarding ASR performance and AI-human agreement on fidelity ratings.

22-ii) Highlight unanswered new questions, suggest future research

Highlight unanswered new questions, suggest future research.

	1	2	3	4	5	
subitem not at all important	○	○	○	○	◉	essential

Clear selection

Does your paper address subitem 22-ii?

Copy and paste relevant sections from the manuscript (include quotes in quotation marks "like this" to indicate direct quotes from your manuscript), or elaborate on this item by providing additional information not in the ms, or briefly explain why the item is not applicable/relevant for your study

"Future Directions: shamiriAI in the Broader Vision" section and "The dimension-level patterns observed here specify a clear development agenda for shamiriAI v2: targeted prompt redesign for the dimensions where AI rated systematically lower than humans (Required Contents and Clarity); refined anchors for facilitation dimensions that distinguish technique frequency from contextual depth; sampling-rate normalization before prosodic feature extraction; and empirical within-corpus evaluation of zero-shot ASR, diarizations, PII redaction, and prosodic-feature ablation. Beyond v2, multi-hub replication and cross-cultural extension to non-Shamiri group interventions and other LMIC contexts should inform future work."
We include a dedicated "Future Directions" section that highlights specific areas for future research and development based on the pilot's findings.

20) Trial limitations, addressing sources of potential bias, imprecision, and, if relevant, multiplicity of analyses

20-i) Typical limitations in ehealth trials

Typical limitations in ehealth trials: Participants in ehealth trials are rarely blinded. Ehealth trials often look at a multiplicity of outcomes, increasing risk for a Type I error. Discuss biases due to non-use of the intervention/usability issues, biases through informed consent procedures, unexpected events.

	1	2	3	4	5	
subitem not at all important	○	○	○	○	◉	essential

Clear selection

Does your paper address subitem 20-i? *

Copy and paste relevant sections from the manuscript (include quotes in quotation marks "like this" to indicate direct quotes from your manuscript), or elaborate on this item by providing additional information not in the ms, or briefly explain why the item is not applicable/relevant for your study

"As a pilot validation, this study has limitations that constrain inference and shape priorities for ongoing development and future research. First, the 52-session validation sample is small for stable agreement estimation in a ceilingaffected distribution. Confidence intervals on AC2 are wide and the dimension-level patterns should be replicated with larger samples before drawing firm conclusions about reliability magnitudes. Second, the human reference itself shows only Moderate inter-rater reliability and substantial restriction of range, which together cap the AI–human agreement any model could achieve against this composite... Fifth, the PII redaction pipeline was not formally evaluated for precision and recall on the Shamiri corpus..."

21) Generalisability (external validity, applicability) of the trial findings

NPT: External validity of the trial findings according to the intervention, comparators, patients, and care providers or centers involved in the trial

21-i) Generalizability to other populations

Generalizability to other populations: In particular, discuss generalizability to a general Internet population, outside of a RCT setting, and general patient population, including applicability of the study results for other organizations

	1	2	3	4	5	
subitem not at all important	○	○	○	○	◉	essential

Clear selection

Does your paper address subitem 21-i?

Copy and paste relevant sections from the manuscript (include quotes in quotation marks "like this" to indicate direct quotes from your manuscript), or elaborate on this item by providing additional information not in the ms, or briefly explain why the item is not applicable/relevant for your study

"Ninth, all sessions were drawn from a single hub in Kajiado County. Demographic subgroup analyses detected no bias by lay-provider sex or age band within the present, but generalizability to other hubs, regions, languages, or non-Shamiri interventions should be tested in multi-site replication."

21-ii) Discuss if there were elements in the RCT that would be different in a routine application setting

Discuss if there were elements in the RCT that would be different in a routine application setting (e.g., prompts/reminders, more human involvement, training sessions or other co-interventions) and what impact the omission of these elements could have on use, adoption, or outcomes if the intervention is applied outside of a RCT setting.

	1	2	3	4	5	
subitem not at all important	○	○	○	○	◉	essential

Clear selection

Does your paper address subitem 21-ii?

Copy and paste relevant sections from the manuscript (include quotes in quotation marks "like this" to indicate direct quotes from your manuscript), or elaborate on this item by providing additional information not in the ms, or briefly explain why the item is not applicable/relevant for your study

"shamiriAI is designed as a clinician-facing decision-support tool: supervisors remain the primary decisionmakers, and AI-generated feedback is treated as one structured input alongside direct clinical knowledge."
"The immediate development priority is shamiriAI v2: ...integration into shamiriOS [37] — Shamiri's digital operational platform enabling automated fidelity reports for every session at every active delivery site as a routine quality assurance function, replacing the 10–15% of sessions currently receiving any human review [20,77]."

OTHER INFORMATION

23) Registration number and name of trial registry

Does your paper address CONSORT subitem 23? *

Copy and paste relevant sections from the manuscript (include quotes in quotation marks "like this" to indicate direct quotes from your manuscript), or elaborate on this item by providing additional information not in the ms, or briefly explain why the item is not applicable/relevant for your study

"The trial was registered with the Pan African Clinical Trials Registry on 1 August 2025 (PACTR202508900479778)."

24) Where the full trial protocol can be accessed, if available

Does your paper address CONSORT subitem 24? *

Cite a Multimedia Appendix, other reference, or copy and paste relevant sections from the manuscript (include quotes in quotation marks "like this" to indicate direct quotes from your manuscript), or elaborate on this item by providing additional information not in the ms, or briefly explain why the item is not applicable/relevant for your study

"Complete intervention protocols are published elsewhere [15,18,22]. See Supplement A for more information."
"See the supervision protocol in Supplement B."

25) Sources of funding and other support (such as supply of drugs), role of funders

Does your paper address CONSORT subitem 25? *

Copy and paste relevant sections from the manuscript (include quotes in quotation marks "like this" to indicate direct quotes from your manuscript), or elaborate on this item by providing additional information not in the ms, or briefly explain why the item is not applicable/relevant for your study

"This work was supported by (1) the Johnson & Johnson QuickFire Challenge... and (2) accelerator funding from the Wellcome Trust. The funders had no role in study design, data collection and analysis, decision to publish, or preparation of the manuscript."

X27) Conflicts of Interest (not a CONSORT item)

X27-i) State the relation of the study team towards the system being evaluated

In addition to the usual declaration of interests (financial or otherwise), also state the relation of the study team towards the system being evaluated, i.e., state if the authors/evaluators are distinct from or identical with the developers/sponsors of the intervention.

	1	2	3	4	5	
subitem not at all important	○	○	○	○	◉	essential

Clear selection

Does your paper address subitem X27-i?

Copy and paste relevant sections from the manuscript (include quotes in quotation marks "like this" to indicate direct quotes from your manuscript), or elaborate on this item by providing additional information not in the ms, or briefly explain why the item is not applicable/relevant for your study

"SL, BM, TO, WM, RK, FK, and RD are employees of Shamiri Institute, a non-profit organization based in the Republic of Kenya. CW is a member of the Board of Directors of Shamiri Institute. The authors declare no other competing interests."

About the CONSORT EHEALTH checklist

As a result of using this checklist, did you make changes in your manuscript? *

- ( ) yes, major changes
- ( ) yes, minor changes
- (x) no

What were the most important changes you made as a result of using this checklist?

Your answer

How much time did you spend on going through the checklist INCLUDING making changes in your manuscript *

Approximately three hours

As a result of using this checklist, do you think your manuscript has improved? *

- (x) yes
- ( ) no
- ( ) Other:

Would you like to become involved in the CONSORT EHEALTH group?

This would involve for example becoming involved in participating in a workshop and writing an "Explanation and Elaboration" document

- ( ) yes
- (x) no
- ( ) Other:

Clear selection

Any other comments or questions on CONSORT EHEALTH

Your answer

STOP - Save this form as PDF before you click submit

To generate a record that you filled in this form, we recommend to generate a PDF of this page (on a Mac, simply select "print" and then select "print as PDF") before you submit it.

When you submit your (revised) paper to JMIR, please upload the PDF as supplementary file.

Don't worry if some text in the textboxes is cut off, as we still have the complete information in our database. Thank you!

Final step: Click submit !

Click submit so we have your answers in our database!

Submit

Clear form

Never submit passwords through Google Forms.

This form was created outside of your domain. - Contact form owner - Terms of Service - Privacy Policy

Does this form look suspicious? Report

Google Forms
